# Universal Plant DNA Barcode Loci May Not Work in Complex Groups: A Case Study with Indian *Berberis* Species

**DOI:** 10.1371/journal.pone.0013674

**Published:** 2010-10-27

**Authors:** Sribash Roy, Antariksh Tyagi, Virendra Shukla, Anil Kumar, Uma M. Singh, Lal Babu Chaudhary, Bhaskar Datt, Sumit K. Bag, Pradhyumna K. Singh, Narayanan K. Nair, Tariq Husain, Rakesh Tuli

**Affiliations:** 1 Department of Plant Molecular Biology and Genetic Engineering, National Botanical Research Institute (Council of Scientific and Industrial Research), Lucknow, Uttar Pradesh, India; 2 Department of Plant Biodiversity and Conservation Biology, National Botanical Research Institute (Council of Scientific and Industrial Research), Lucknow, Uttar Pradesh, India; Montreal Botanical Garden, Canada

## Abstract

**Background:**

The concept of DNA barcoding for species identification has gained considerable momentum in animals because of fairly successful species identification using cytochrome oxidase I (*COI)*. In plants, *matK* and *rbcL* have been proposed as standard barcodes. However, barcoding in complex genera is a challenging task.

**Methodology and Principal Findings:**

We investigated the species discriminatory power of four reportedly most promising plant DNA barcoding loci (one from nuclear genome- ITS, and three from plastid genome*- trnH-psbA*, *rbcL* and *matK*) in species of Indian *Berberis* L. (Berberidaceae) and two other genera, *Ficus* L. (Moraceae) and *Gossypium* L. (Malvaceae). *Berberis* species were delineated using morphological characters. These characters resulted in a well resolved species tree. Applying both nucleotide distance and nucleotide character-based approaches, we found that none of the loci, either singly or in combinations, could discriminate the species of *Berberis*. ITS resolved all the tested species of *Ficus* and *Gossypium* and *trnH-psbA* resolved 82% of the tested species in *Ficus*. The highly regarded *matK* and *rbcL* could not resolve all the species. Finally, we employed amplified fragment length polymorphism test in species of *Berberis* to determine their relationships. Using ten primer pair combinations in AFLP, the data demonstrated incomplete species resolution. Further, AFLP analysis showed that there was a tendency of the *Berberis* accessions to cluster according to their geographic origin rather than species affiliation.

**Conclusions/Significance:**

We reconfirm the earlier reports that the concept of universal barcode in plants may not work in a number of genera. Our results also suggest that the *matK* and *rbcL*, recommended as universal barcode loci for plants, may not work in all the genera of land plants. Morphological, geographical and molecular data analyses of Indian species of *Berberis* suggest probable reticulate evolution and thus barcode markers may not work in this case.

## Introduction

DNA barcoding is the use of short DNA sequences for species identification. Since its inception as an approach for large scale species identification [1]–[3], several studies have reported the application of *COI* in a wide range of animal taxa [1], [4]–[6]. However, the attempts to identify a single locus for barcoding in plants have largely been unsuccessful [7], [8]. There are growing evidences which suggest the need of deploying more than one locus for barcoding in plants [9]–[13]. The following regions have been suggested for plant DNA barcoding: ITS (the internal transcribed spacer region of the nuclear ribosomal genes), *rbcL* and *psbA-trnH* [10]; ITS and *psbA-trnH* [14]; *rbcL* [15]; *rpoC1*, *rpoB* and *matK* or *rpoC1*, *matK* and *psbA-trnH* [9]; *matK*, *atpF-atpH* and *psbA-trnH* or *matK* and *psbK-psbI* [7], *psbA-trnH* and *rbcL* [11]; *psbA-trnH* [16]; *trnLUAA* [13]; *matK* and *psbA-trnH* [12], *matK* and *rbcL* [17] *matK*, *rbcL* and *trnH-psbA* [18] and ITS2 [19], [20].

In most of the recent plant barcoding studies, the coding regions of *matK* and *rbcL* and the non-coding plastid intergenic spacer of *trnH-psbA* have been suggested as prime candidates for barcoding [17], [18]. Following the first suggestion by Kress *et al*. (2005) [14], several subsequent reports projected *trnH-psbA* as a strong candidate for plant barcoding [9]–[12], [16]. However, Consortium for the Barcoding of Life (CBOL) disregarded *trnH-psbA* as it does not consistently provide bidirectional unambiguous sequencing reads [17]. Erstwhile studies have focused predominantly on plastid regions for barcoding. Chase *et al.* (2005) [10] and Kress *et al*. (2005) [14] recovered highest mean percentage sequence divergence (2.81 and 5.7% respectively) for nrITS region for plant barcoding. However, the use of ITS region as barcode locus has often been considered unfavorable because of the presence of paralogs in several plant taxa. Yet, in other studies, ITS has been successfully used as barcode locus [10], [14], [21]. More recently, ITS2 has been projected as an important plant barcode locus [19], [20]. We examined four DNA barcoding loci (one nuclear- nrITS and three plastid loci- *trnH-psbA*, *rbcL* and *matK*) in 16 species of *Berberis* L. (Berberidaceae) from India. For validation of the techniques, we tested these loci in selected species of two other genera, *Ficus* L. (Moraceae), comprises keystone species in tropical rain forest ecosystems and *Gossypium* L. (Malvaceae), a pan tropical genus including the commercial cotton plants cultivated widely on the tropical and subtropical regions throughout the world.

**Figure 1:**
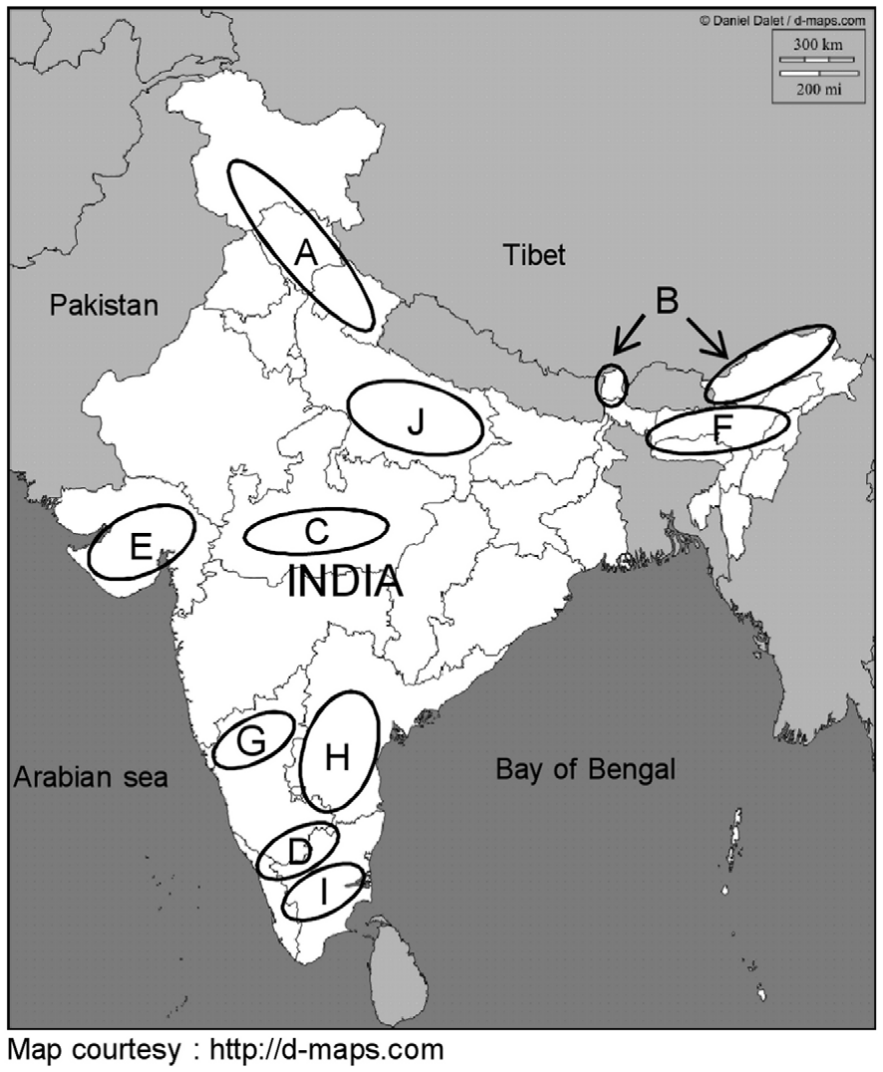
The geographical distribution of different species of *Berberis*, *Ficus and Gossipium*. Circles indicate the regions from where species were collected. Exact GPS data are shown in Table S1. Region A (western Himalayas): *B. aristata, B. asiatica, B. chitria, B. glaucocarpa, B. jaeschkeana, B. lycium, B. pachyacantha, B. umbellata;* Region B (Eastern Himalayas): *B. angulosa, B. griffithiana, B. insignis, B. macrosepala, B. replicata;* Region C (Satpura - Central India): *B. Hainesii;* Region D (Nilgiri Hills-Southern India): *B. tinctoria, B. wightiana; Region E* (Gujrat): *G. herbaceum, G. arboreum* Region F (Assam): *G. barbadense*; Region G (Karnataka): *G. herbaceum, G. arboretum, G. hirsutum* Region H (Andhra Pradesh): *G. hirsutum;* Region I (Tamil Nadu): *G. barbadense*; Region J (Uttar Pradesh): *Ficus* species.

The genus *Berberis* comprises of about 500 species [22], [23]. Based on phytogeographic distribution Schneider (1905) divided the species of *Berberis* into two groups, Septentrionales and Australes, [23]. The group Septentrionales (Old World) consists of ca 300 species occurring mainly in Eurasia but extending to North America (two species) and North Africa (four species). The group Australes (New World) contains about 200 species with most of them distributed in South America and a few in Middle America [22], [24]. These geographical groups are supported by grouping based on morphological characters [23]. A recent molecular study based on the internal transcribed spacer (ITS) sequences, supports the treatment of these two groups within *Berberis* [25]. In subdividing these groups, Ahrendt (1961) accepted the schemes of Schneider (1905) and recognized 29 sections with some modifications. Ahrendt (1961) further subdivided these sections into numerous subsections. In India, *Berberis* is represented by 55 species [26], which according to Ahrendt (1961) belong to 8 sections and 7 subsections [22]. The majority of the species are centered in the Himalayan region extending from Pakistan to Western China and to Central and Southern China. The 16 species selected for the present study represent wide geographical distribution across India and belong to 5 sections and subsections (Table S1). The detailed morpho-taxonomic characters of these sections and subsections were described by Ahrendt (1961) [22]. The geographical locations of the selected species are indicated in Figure 1. Some species of *Berberis* are known for high medicinal value because of the presence of alkaloids, principally ‘berberine’ [27], which show activity against cholera, diarrhea, amoebiasis, malaria and leishmaniasis [28]. Some species of *Berberis* give a high value wood dye while some others provide edible berries.

The taxonomy of *Berberis* is somewhat uncertain [27]. For example, Orisi (1984) considered 17 Patagonian species in Argentina [29], where as Landrum (1999) synonymized several of these species and recognized only nine [24]. Complexity in *Berberis* taxonomy has been attributed to hybridization and some degree of introgression in transitional zones which produce intermediate forms [27]. Out of 500 species of *Berberis*, Ahrendt (1961) recorded as many as 70 species and infraspecific taxa with a suspected hybrid origin [22]. Based on molecular phylogeny of *Berberis* species and previous taxonomic treatment of Landrum (1999) [24], Kim *et al* (2004) questioned the status of most sections and subsections in this genus [25]. Although taxonomic revision of Indian *Berberis*, based on morpho-taxonomic parameters has been proposed [26], no attempt has been made to establish the species delimitation in Indian *Berberis* using molecular approaches. In this study, we examined the application of standard plant DNA barcode loci in this challenging group considering Indian species of *Berberis*.

In order to test the universality of the standard barcode loci we applied these loci to *Ficus* and *Gossypium*. Although, taxonomically the genus *Ficus* is considered to be quite difficult, it forms distinct natural groups in which many species are very common and conspicuous and can easily be identified even with sterile specimens [30]. *Ficus* consists of about 1000 species of woody trees, shrubs, vines, epiphytes and hemi- epiphytes [31], occurring in most tropical and subtropical forests thought the world. In India, the genus comprises about 100 species which are distributed throughout the country with maximum diversity in Western Ghats and North Eastern India [32], [33]. The candidate species of *Ficus* selected in the present study belong to three subgenera and six sections (Table S2) [31]. Earlier phylogenetic study using ITS sequences indicates that three subgenera of *Ficus* studied here are monophyletic [34]. The four species of *Gossypium* considered here are well studied both taxonomically and at molecular diversity level elsewhere [35], [36].

## Results

### *Berberis*

morphometric analysis, 3–5 representative accessions from each species of *Berberis* were studied, as described in material and methods. The character matrix thus developed is shown in Table S3. A consensus parsimony tree was developed using the matrix. The cladogram showed clear segregation of the accessions into distinct species clades (Figure S1).

**Figure 2:**
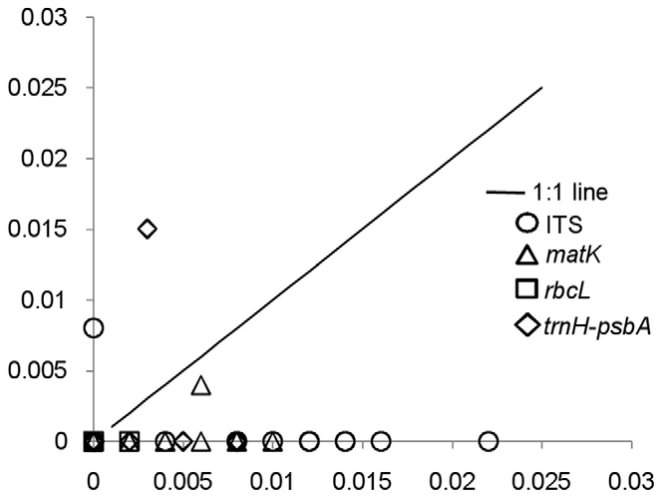
Presence/Absence of barcode gap. Sequence divergence for different gene regions in different species of *Berberis*. X-axis: maximum intraspecific, Y-axis: minimum interspecific K2P distances.

For DNA barcoding, we examined 129 DNA sequences for ITS (GenBank accession numbers GU934610-GU934738), 78 for *matK* (GenBank accession numbers GU934739-GU934816), 97 for *rbcL* (GenBank accession numbers GU934817-GU934913), and 83 for *trnH-psbA* (GenBank accession numbers GU934914-GU934996) representing 16 species of Indian *Berberis*. PCRs were generally successful with all the four loci. The maximum success in PCR was observed with *rbcL* and ITS (97%), followed by *trnH-psbA* (92%) and *matK* (76%). Sequencing success ranged from 95% for *rbcL* to 85% for *matK* (Table 1). The alignment of sequences was straight forward except in case of *trnH-psbA*, due to high variation in sequence length. The mean sequence lengths of ITS (ITS1+5.8S+ITS2), *matK, rbcL* and *trnH-psbA* were 602.2, 488.1, 479.0 and 410.0 bp, respectively. The corresponding percentage frequencies of parsimony informative characters were 4.9, 1.8, 0.6 and 2.6 and the percentage variable sites were 6.1, 3.0, 0.8, and 3.2, respectively (Table 1). The genetic divergence within and between species was calculated. The highest mean intraspecific divergence was obtained in ITS and the lowest mean inter- and intraspecific divergence was obtained in case of *rbcL* (Table 1). ANOVA test showed ITS and *trnH-psbA* as the most divergent barcode loci at interspecific level followed by *matK* and *rbcL* (Table S4 A). At intraspecific level, *rbcL* was the least and ITS was the most divergent locus (Table S4 B). In multilocus analysis, ITS+*trnH-psbA* provided the highest divergence at interspecific level as compared to the two, three and four loci combinations (Table S5 A). At intra specific level, there was no significant difference in the sequence divergence between all combinations (Table S5 B). To evaluate the barcoding gap we looked at the minimum inter- and maximum intraspecific divergences for each locus. No distinct barcoding gap was noticed in any of the four loci (Figure 2, Table S6). To detect paralogs of ITS, if any, we cloned the PCR product from eight randomly selected species and sequenced at least eight clones from each species. None of the species showed the presence of multiple copies as evident from sequence data (GenBank accession numbers HM347877 to HM347940).

**Table 1:** Comparison of four loci tested on *Berberis*, *Ficus* and *Gossypium*.

	*Berberis*				*Ficus*				*Gossypium*			
Locus	ITS	*matK*	*rbcL*	*trnH-psbA*	ITS	*matK*	*rbcL*	*trnH-psbA*	ITS	*matK*	*rbcL*	*trnH-psbA*
Number of species	16.0	13.0	15.0	14.0	11.0	8.0	11.0	11.0	4.0	4.0	4.0	4.0
Number of sequences	129.0	78.0	97.0	83.0	33.0	25.0	32.0	31.0	51.0	46.0	42.0	37.0
Mean sequence length	602.2	488.1	479.0	410.0	619.7	704.5	630.1	386.0	551.1	628.5	626.1	418.3
% PCR success	97.0	76.0	97.0	92.0	100.0	85.0	100.0	91.0	100.0	100.0	100.0	100.0
% Sequencing success#	94.0	85.0	95.0	90.0	100.0	85.0	98.0	91.0	100.0	100.0	100.0	100.o
% PIC*	4.9	1.8	0.6	2.6	21.7	1.9	0.6	6.9	8.0	0.9	0.3	1.8
% Variable sites	6.1	3.0	0.8	3.2	30.0	3.6	1.2	7.1	8.7	3.2	1.0	3.2
Mean inter-specific distances (K2P)	0.011 (0.002)†	0.005 (0.001)	0.001 (0.0005)	0.009 (0.003)	0.0824 (0.053)	0.008 (0.004)	0.001 (0.001)	0.017 (0.009)	0.051 (0.031)	0.002 (0.004)	0.002 (0.001)	0.002 (0.002)
Mean intra-specific distances (K2P)	0.003 (0.002)	0.001 (0.001)	0.0001 (0.0003	0.0009 (0.001)	0.004 (0.003)	0.004 (0.004)	0.0	0.0	0.002 (0.002)	0.002 (0.0039)	0.001 (0.001)	0.001 (0.001)

*Parsimony informative characters.

† Figures in parenthesis indicate standard deviation.

# Percent sequencing success refers to the fraction of sequences having QV above 30 and at least 70% overlap between sequence reads using forward and reverse primers (except in some cases of *matK*) of total number of PCR products.

Phylogenetic methods were applied using each barcode locus taken alone and in combinations to evaluate species recovery. The NJ, MP and UPGMA methods were used for both single locus (Table 2 and Figure S2) and multilocus analysis with 500 bootstrap replicates (Table 2 and Figure S3). When all the sequences for a given locus were considered, ITS, *matK* and *trnH-psbA* were able to form species specific clade only in case of *B. pachyacantha*. Not a single species was recovered with *rbcL* using any of the three methods. The clades formed in the trees were mostly mixtures of several species. While the species specific clades in ITS, *trnH-psbA* and *matK* trees resolved with high bootstrap confidence levels (76–99%), the internal branches of the non species-specific clades with mixtures of species showed low bootstrap support (0 to 65%). A data set of 58 sequences representing 13 species was prepared to have at least three common accessions (except in *B. angulosa*) which were sequenced using all the four loci. In this data set, the species recovery and their bootstrap support increased for all the four loci (Table 2 and Figure S4). In multilocus analysis, we used combinations of two, three and four loci to see if species recovery was better than in single locus analysis (Table 2, Figure S3). In two loci combination, highest species recovery was observed with ITS+*trnH-psbA* and ITS+*matK* at 30.8% followed by *matK+rbcL* at 23.1% and ITS+*rbcL* at 15.4%. All three-locus combinations yielded maximum species recovery at 30.8% except ITS+*trnH-psbA+rbcL* which yielded 23.1% species recovery. The four loci combination did not provide better species recovery as compared to the best performing two or three loci combinations. We did not find any significant difference using three methods of phylogenetic tree construction with single locus analysis as far as recovery of species in *Berberis* is concerned. However, in some cases of multilocus analysis, NJ method provided better species recovery as compared to MP and UPGMA.

**Table 2:** Proportion (%) of monophyletic species recovered with different phylogenetic methods using four individual loci and their combinations.

Genus	Locus	NJ	MP	UPGMA
*Berberis* *	ITS	6.3	6.3	6.3
	*matK*	7.7	7.7	7.7
	*rbcL*	0.0	0.0	0.0
	*trnH-psbA*	7.1	7.1	7.1
*Berberis* †	ITS	7.7	23.1	15.4
	*matk*	7.7	15.4	15.4
	*rbcL*	7.7	7.7	7.7
	*trnH-psbA*	15.4	15.4	15.4
	ITS*+matK*	30.8	30.8	30.8
	ITS*+rbcL*	15.4	15.4	15.4
	ITS*+trnH-psbA*	30.8	23.1	23.1
	*matK+rbcL*	23.1	15.4	15.4
	*trnH-psbA+matK*	15.4	15.4	15.4
	*trnH-psbA+rbcL*	23.1	15.4	23.1
	ITS*+matK+rbcL*	23.1	30.8	15.4
	ITS*+trnH-psbA+matK*	23.1	23.1	23.1
	*trnH-psbA+matK+rbcL*	15.4	23.1	15.4
	ITS*+trnH-psbA+rbcL*	30.8	23.1	23.1
	ITS*+trnH-psbA+matK+rbcL*	30.8	23.1	15.4
*Ficus*	ITS	100.0	90.9	100.0
	*matK*	25.0	25.0	0.0
	*rbcL*	9.1	0.0	9.1
	*trnH-psbA*	63.6	81.8	81.8
*Gossypium*	ITS	100.0	100.0	75.0
	*matK*	0.0	0.0	0.0
	*rbcL*	0.0	0.0	0.0
	*trnH-psbA*	25.0	0.0	0.0

*When all the sequences for individual locus are considered.

† When data set was reduced to 58 sequences representing at least three common accessions per species for each locus.

**Figure 3:**
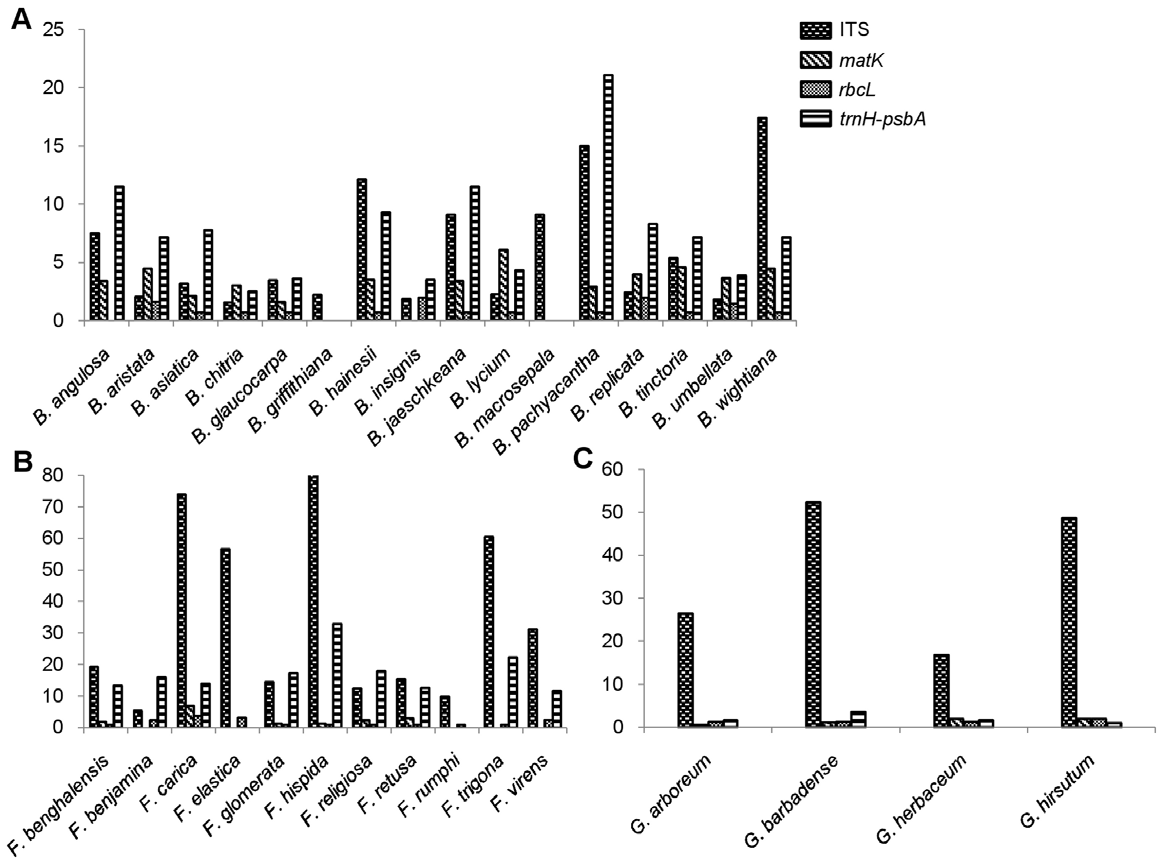
Ratios of mean inter-and intraspecific K2P distances of different species of *Berberis* (A), *Ficus* (B) and *Gossypium* (C). Ratios could not be determined in some species where there was either single accession or sequencing failure in some loci.

Character-based method was applied for species delineation as an alternative to the genetic divergence based approach, as diagnostic characters prevent the loss of information characteristic to distance approach [37], [38]. Using simple (characters which are confined to a single nucleotide position) or compound (combined states at multiple nucleotide positions) diagnostic characters, it was observed that except in *B. pachyacantha*, none of the loci showed unique diagnostic character(s) to distinguish species in *Berberis* (Table 3). Species discrimination was also calculated using the criteria reported by CBOL plant working group [17] i.e. discrimination was considered successful, if the minimum interspecific K2P (Kimura-2-parameter) distance involving a species was larger than its maximum intraspecific K2P distance. No one species but *B. pachyacantha* exhibited minimum interspecific K2P distance higher than the maximum intraspecific K2P distances with ITS sequences and *trnH-psbA*. Other two loci did not meet these criteria in any of the species in *Berberis* (Table S7). The ratios of interspecific and intraspecific K2P distances were calculated for all the loci. ITS exhibited the highest inter to intraspecific K2P distances ratio, followed by *trnH-psbA* (Figure 3A).

**Table 3:** Diagnostic characters for each species in ITS, *matK, rbcL* and *trnH-psbA*.

		ITS	*matK*	*rbcL*	*trnH-psbA*
	Ref. genbank accession No.	AF403383.1	AB038184.1		GQ435419.1
*B. pachyacantha*		29-T, 107-T, 526-G, 561-A, 562-T	824-T, 1055-G		476-C, 399-A, 248-A, 219-G
	Ref. genbank accession No.	AY730065.1	EU002177.1	EU002278.1	GQ982218.1
*F. bengalensis*		73-A			
*F. benjamiana*		62-C			379-G
*F. carica*		65-T, 161-G, 202-T, 413-G, 443-T, 447-T, 480-T, 485-T, 507-C	964-T	433-A, 684-T	279-T
*F. elastica*		78-G+181-G			
*F. rumphi*		450-T			
*F. glomerata*		42-T, 45-A, 56-A, 65-A, 74-T, 75-A, 78-T, 81-T, 106-A, 107-T, 129-T, 132-T, 138-A, 157-T, 159-A, 200-G, 219-T, 259-A, 285-C, 288-A, 289-T, 297-T, 308-A, 367-T, 372-T, 379-T, 382-T, 383-A, 385-C, 386-C, 418-A, 440-A, 442-T, 448-A, 453-A, 471-T, 474-T, 486-A, 492-T, 502-G, 509-A	600-T, 601-T		255-G
*F. hispida*		167-C, 188-C, 200-A, 201-C, 408-G, 415-A, 510-G, 515-A	583-G,		188-T
*F. retusa*		51-A			
*F. religiosa*		66-A, 86-T			280-G, 156-T
*F. trigona*		66-C, 71-G, 181-C, 466-A			228-A, 212-C
*F. virens*		36-G+ (66-T/86-C), 160-T+(66-T or 86-C)		690-A	
					
	Ref. genbank accession No.	AF057757.1			DQ345959.1
*G. arboreum*		154-C			
*G. barbadense*		103-C			235-G
*G. herbaceum*		193-T, 569-T			
*G. hirsutum*		80-G+274-G			

Diagnostic characters for each locus were identified with reference to GenBank sequence for the locus of particular species or related species (if not available in the GenBank data base). For ITS, the start of ITS1 of reference sequence was considered as position 1, for *matK* and *rbcL*, the first nucleotide of start codon of reference sequence was considered as position 1 and for *trnH-psbA*, the first position of intergenic spacer of reference sequence was considered as position 1.

### *Ficus* and *Gossypium*

**Figure 4:**
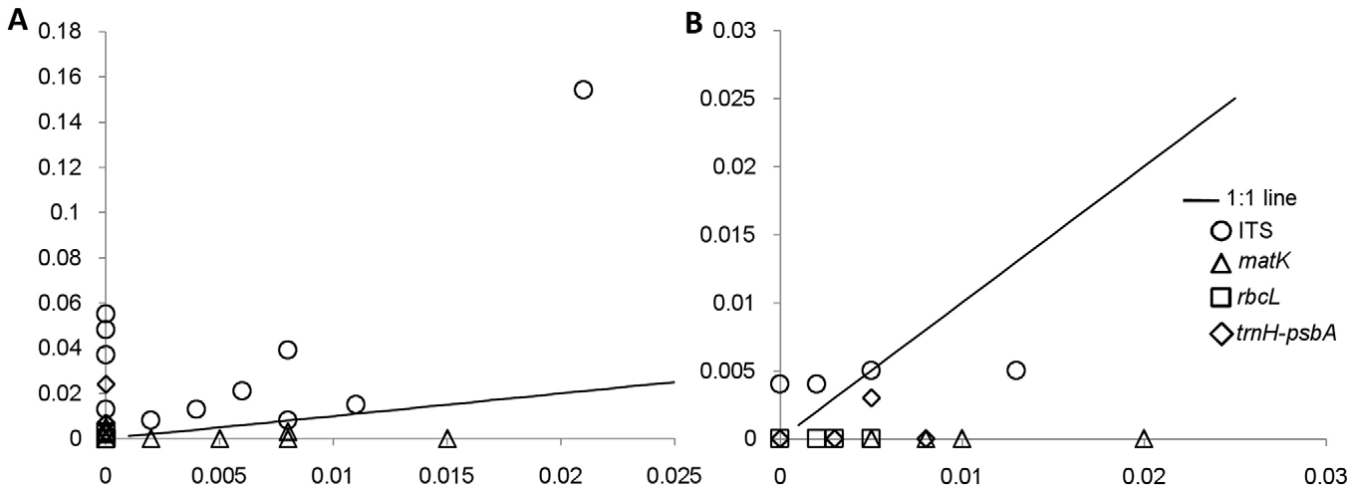
Presence/Absence of barcode gap. Sequence divergence for different gene regions for different species of *Ficus* (A) and *Gossypium* (B). X-axis: maximum intraspecific, Y-axis: minimum interspecific K2P distances.

We tested all the four loci in *Ficus* and *Gossypium* to validate applicability of barcoding loci in unrelated genera. In all, we analyzed 33 accessions representing 11 species of *Ficus* (GenBank accession numbers ITS; HM368181-HM368213, *matk*; GU935030-GU935054, *rbcL*; GU935055-GU935086, *trnH-psbA*; GU935087-GU935117) and 51 accessions representing four species of *Gossypium* (GenBank accession numbers ITS; GU935118-GU935168, *matk*; GU935169-GU935214, *rbcL*; GU935215-GU935256, *trnH-psbA*; HM437871-HM437907). PCR amplification and sequencing were largely successful with all the four loci (Table 1). The mean sequence length, parsimony informative characters and percent variable sites for the four loci are shown in Table 1. In both *Ficus* and *Gossypium*, ITS exhibited the highest interspecific and *rbcL* and *trnH-psbA* the lowest intraspecific divergence (Table 1). In ANOVA test, ITS emerged as the most divergent barcode locus at interspecific level than all other loci in *Ficus* and *Gossypium* (Table S8). At intraspecific level *rbcL* and *trnH-psbA* were the least divergent loci in *Ficus* where as in *Gossypium*, all loci were equally divergent at inra specific level. The highest inter- to intraspecific K2P distance ratios were exhibited by ITS in both the genera (Figure 3B and C). In the phylogenetic analysis of *Ficus*, ITS exhibited 100% species recovery (Figure S5A, Table 2) followed by trnH*-psbA* (82%) (Figure S5D, Table 2). In *Gossypium*, ITS recovered 100% species (Figure S6A). Other loci could not distinguish the species in the two genera. Since all tested species of both *Ficus* and *Gossypium* were recovered using ITS, we did not apply multilocus combinations in these genera. ITS and *trnH-psbA* exhibited distinct barcoding gap in *Ficus* (Figure 4) where the minimum inter specific K2P distances were significantly higher than the maximum intraspecific K2P distances (t test, p = 0.02, and 0.006 respectively, Table S6). In *Gossypium*, none of the loci showed significant barcoding gaps (Table S6). In character-based approach, diagnostic characters were found in the ITS sequences of *Ficus* and *Gossypium*. Nine species of *Ficus* exhibited simple diagnostic characters in single or multiple positions and two species, *F. elastica* and *F. virens* exhibited compound characters. All the four species of *Gossypium* were identified by simple or compound diagnostic characters using ITS. *trnH-psbA* exhibited simple diagnostic character at single position in four species and simple diagnostic characters at multiple positions in other two species of *Ficus. trnH-psbA* showed simple diagnostic character at single position in *G. barbadense*. The *matK* and *rbcL* did not provide any diagnostic characters in *Gossypium* species. However, in case of *Ficus*, *matK* and *rbcL* exhibited simple diagnostic characters in three species (*F. carica, F. hispida* and *F. recemosa*) and two species *(F. carica*, *F. virens*) respectively (Table 3). In distance-based approach, the highest intraspecific K2P distance of *trnH-psbA* was lower than the lowest interspecific K2P distance in all the species of *Ficus*. Similar results were obtained using ITS except in *F. religiosa* where maximum intraspecific K2P distance was equal to minimum interspecific K2P distance. In *Gossypium*, the highest intraspecific K2P distance of ITS was lower than the lowest interspecific K2P distance in *G*. *hirsutum* and *G. barbadense* but not in case of *G*. *herbaceum* and *G*. *arboretum* (Table S9).

### AFLP analysis of *Berberis* species

**Figure 5:**
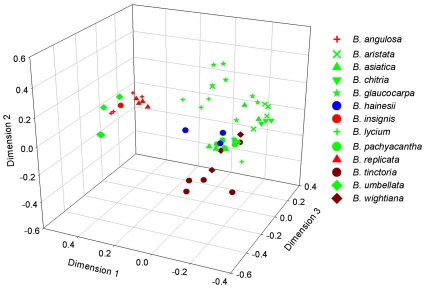
Results of principal coordinate analysis of AFLP markers for *Berberis* species showing separation of two clusters as revealed by phenograms. The three vectors, one, two and three contribute 10.2%, 4.9 and 3.6% of total variability respectively. Colors represent regions from where species were collected. Green: Western Himalaya, Red: Eastern Himalaya, Blue: Central India and Dark Red: Southern India.

**Figure 6:**
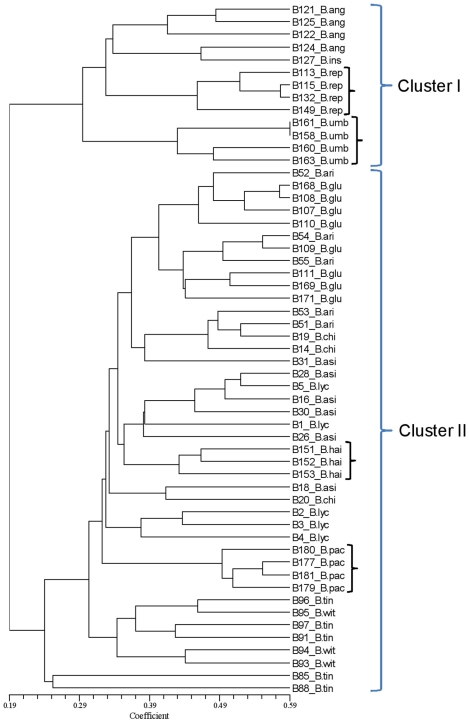
UPGMA cluster analysis thirteen species of *Berberis* based on Jaccard's coefficient similarities of AFLP molecular banding patterns. The numbers preceding the abbreviated species name denotes the DNA number. Abbreviations are: B.ang: *Berberis angulosa*, B.ari: *Berberis aristata*, B.asi: *Berberis asiatica*, B.chi: *Berberis chitria*, B.glu: *Berberis glaucocarpa*, B.hai: *Berberis hainesii*, B.ins: *Berberis insignis*, B.lyc: *Berberis lycium*, B.pac: *Berberis pachyacantha*, B.rep: *Berberis replicata*, B.tin: *Berberis tinctoria*, B.umb: *Berberis umbellata*, B.wit: *Berberis wightiana*.

Using ten different primer combinations, a total of 784 bands were scored, 776 were polymorphic representing 98.9% of the total number of bands (Table 4). The number of bands varied among species. The distribution of total bands in different species is shown in Table 5. The number of polymorphic bands ranged from 46 for *EcoR*I+ACA/*Mse*I+CTG to 97 for *EcoR*I+AAG/*Mse*I+CAT (Table 4). Among the polymorphic bands, only eight bands were unique representing the three species, *B. insignis*, *B. pachyacantha* and *B. replicata*. The results of principal coordinate analysis, based on Jaccard's coefficient of similarity are shown in Figure 5. The first three cumulatively accounted for 18.7% of the total variance detected, comprising 10.2%, 4.9% and 3.6% from the first, second and third vectors respectively. Ordination of the first vector with second and third showed two distinct clusters corresponding to species of Eastern Himalayas, *B. replicata*, *B. angulosa* and *B. insignis* along with one species of Western Himalayas *B. umbellata*, and the remaining species formed a second cluster. The trends revealed by principal coordinate analysis were supported by UPGMA cluster analysis based on Jaccard's similarity matrix (Figure 6). The phenogram largely recognized the major two clusters as that of principal coordinate analysis. Within the cluster I, the species of *B. umbellata*, and *B. replicata*, and in cluster II the species of *B. pachyacantha* and *B. hainesii* were well separated. Other species were not recognized by AFLP method. However, there was a tendency of these species to cluster according to geographic location rather than species affiliation. For example, *B. replicata*, *B. angulosa* and *B. insignis* are exclusively from Eastern Himalayas grouped in one cluster. Similarly the species from central part of India, *B. hainesii* formed one distinct cluster while the species from Southern part of India, *B. tinctoria*, and *B. wightiana* formed another distinct cluster. The remaining species are from Western Himalayas and were mostly scattered. The cophenetic correlation coefficient was 0.86 (*p*≤0.01), which indicates good agreement with the cluster analysis with the original distance matrix. In addition, the Mantel test between Jaccard's distance matrix based on AFLP and geographical distance matrix derived from GPS data of respective samples was quite strong (r = 0.46, *p*≤0.001).

**Table 4:** Level of polymorphism, fingerprinting patterns and unique bands of AFLP markers in different species of *Berberis*.

Primer pair	No. of Bands	No. of Polymorphic Bands	% Polymorphic Bands	Unique Bands (species)
P1. *EcoR*I+AAG/*Mse*I+CAA	85	84	98.8	1 (*B. insignis*)
P2. *EcoR*I+AAG/*Mse*I+CAT	97	97	100	0
P3. *EcoR*I+AAC/*Mse*I+CTT	88	88	100	0
P4. *EcoR*I+ AAC/*Mse*I+CTG	64	64	100	0
P5. *EcoR*I+AGG/*Mse*I+CAA	82	82	100	0
P6. *EcoR*I+AGG/*Mse*I+CTG	76	72	94.74	4 (*B. insignis-3, B. replicata-1*)
P7. *EcoR*I+ ACC/*Mse*I+CTC	88	88	100	0
P8. *EcoR*I+ACC/*Mse*I+CTT	94	93	98.94	1 (*B. pachycantha*)
P9. *Eco.R*I+ACG/*Mse*I+CAC	64	62	96.88	2 (*B. insignis*)
P10. *EcoR*I+ACA/*Mse*I+CTG	46	46	100	0

**Table 5:** Distribution of total bands of AFLP in different species using different primer combinations.

	Primer pair*									
Species	P1	P2	P3	P4	P5	P6	P7	P8	P9	P10
*B. angulosa*	48	7	33	0	52	40	38	33	33	0
*B. aristata*	30	54	60	44	9	18	29	35	6	17
*B. asiatica*	39	45	65	42	6	21	31	49	7	26
*B. chitria*	23	46	54	30	5	6	29	37	14	4
*B. glaucocarpa*	29	58	64	43	36	39	50	54	14	21
*B. hainesii*	23	33	41	27	22	23	22	30	9	0
*B. insignis*	29	3	7	0	24	26	13	11	11	0
*B. lycium*	35	48	53	34	34	29	44	48	20	26
*B. pachycantha*	22	24	46	28	13	18	29	36	6	19
*B. replicata*	27	7	32	0	42	31	30	24	20	0
*B. tinctoria*	24	26	37	35	16	19	30	34	7	7
*B. umbellata*	37	4	25	0	37	22	30	30	14	0
*B. wightiana*	19	17	48	27	9	15	26	29	4	15

*Primer name as mentioned in Table 4.

## Discussion

We investigated the species resolution ability of four barcode loci viz. ITS, *matK*, *rbcL*, and *trnH-psbA* in 16 species of *Berberis*, 11 species of *Ficus* and 4 species of *Gossypium*. In our study, the *matK*2.1a failed to give amplification in *Berberis*. The modified *matK* primer was largely successful in PCR amplification in all the three genera. There are mixed reports about PCR success and sequencing using *matK* primers, depending upon the use of particular primer and data sets [11], [12], [17]. However, the other three primers, ITS, *rbcL* and *trnH-psbA* provided good amplification with the three test genera. In spite of successful design of genus specific *matK* primer for *Berberis*, the PCR success rate was lower (76%) in *Berberis* as compared to *Ficus* (85%) and *Gossypium* (100%). This lower success rate of PCR using genus-specific *matK* in *Berberis* may be due to the instability and the uniqueness of the primer's 3′-end in *matK* sequences of *Berberis* samples as reported in other cases [39], [40]. A successful barcode locus is evaluated on the basis of its ability to recover monophyletic clusters corresponding to individual species [41]. Based on this criterion, all the species of *Ficus* and *Gossypium* were recovered using ITS. None of the four loci could resolve the species in *Berberis* except *B. pachyacantha*. However, when we analyzed 58 common sequences for all the four loci with at least three accessions per species, the proportion of species recovery increased with ITS and *trnH-psbA*. The improvement in species recovery by ITS and *trnH-psbA* using this reduced sequence set as compared to initial sequence set of 129 and 83 respectively, was due to removal of some sequences having higher intraspecific divergence than the others within the same species. Such overlap will not affect species identification of unknowns in already characterized species but can have impact in incompletely sampled groups [42].

In *Berberis*, species recovery improved using multilocus combinations as compared to the single locus. The species recovery by two loci combination was as good as three or four loci combinations (30.8%) (Table 2). In most of the studies reported earlier, multilocus analysis of more than three loci did not provide significant gain in species recovery as compared to one or two loci combinations [12], [17], [43], [44]. In most of these reports, ITS was not used in multilocus analysis. Our results indicate that ITS in combination with *trnH-psbA* provided better species discrimination as compared to *matK*+*rbcL* in these tested species for multilocus barcode analysis.

We examined the use of character-based approach as an alternative to distance-based approach for species delineation. In case of *Berberis*, ITS showed diagnostic characters only for the species *B*. *pachyacantha.* This species was also resolved with distance based approach. The tested species of *Ficus* and *Gossypium* were resolved by ITS sequences using either of the methods. Character-based approaches have been shown to be successful in species identification [43], [45]. The study of Rach *et al.* (2008), focused only on simple characters i.e. a standalone diagnostic at a single nucleotide position [45]. Wong *et al.* (2009) examined 64 shark species by character-based approaches [46]. They identified 20 species of shark by simple character and 37 species using compound characters of two or three nucleotide positions in combination. The consideration of simple diagnostic characters at multiple positions in our study increases the level of confidence of a resolved species as compared to a single position. However, we noticed no additional advantage using diagnostic character-based approach over distance-based approach as far as percentage species resolution is concerned.

The four barcode loci did not resolve the sections and subsections of Indian *Berberis* except section ‘vulgaris’, represented by only one species, *B. pachyacantha*. Similarly, although the species of *Ficus* could be resolved using ITS and *trnH-psbA*, they failed to identify the subgenus and sections of the genus. A recent molecular study conducted on a large number of samples (100 species) using three nuclear markers (ITS, ETS and G3pdh) support neither subgeneric nor sectional classification in *Ficus* except in subgenus *Sycidium* [47].

Although *matK* and *rbcL* have been shown to provide high level of species recovery in several plant DNA barcoding studies on different floristic or biodiversity hotspots [9], [11], [12], [17], [19], [30], these loci were not found useful in many other studies dealing with specific taxonomic groups [36]–[40] as in the present study. Another leading barcoding locus proposed by several workers is *trnH-psbA* [11], [12], [14], which also did not work in the tested species of *Berberis* and *Gossypium*. However, it provided good species recovery in *Ficus*. In our study, ITS recovered one species in *Berberis* and all the tested species of *Ficus* and *Gossypium*. Several other studies have also reported ITS as one of the suitable markers for barcoding in plants [14], [21]. Other studies described its inherent difficulties, e.g. low PCR success [9], [11], problem of secondary structure formation, resulting in poor quality sequence data [37], [38] and multiple copy numbers [48], etc. We did not find any difficulty in PCR and sequencing for ITS (Table 1). In addition, no paralogos of ITS were found in *Berberis* and *Ficus* using PCR as well as sequencing of at least eight clones from PCR products of each of the tested eight species of *Berberis*. Natural hybridization and polyploidy are rare in *Ficus* [49]. Therefore divergent paralogs of ITS may be uncommon in *Ficus*. It is also reported that ITS has undergone complete concerted evolution in *Gossypium* following allopolyploid speciation [50]. These studies and our findings in ITS sequences of *Berberis* indicate none of the tested three genera have paralogs of ITS. Our results, especially in *Ficus* and *Gossypium* suggest that ITS holds a good promise as a candidate barcode locus, as also reported earlier [51].

The low level of barcoding success observed in *Berberis* is not uncommon in plants. Similar difficulties have earlier been reported in *Aspalathus* [21], *Crocus* [52] *Solanum* sect. *Petota* [53] and *Carex* [44]. Even in the well studied taxonomic group, barley (*Hordeum* L.), Seberg *et al.* (2009) reported recognition of less than 50% species using *matK* and *rpoC1* [52]. In the morphologically distinct species of the Galapagos sun flower tree, *Scalesia* Arn, (Asteraceae), no variation was found in plastid loci and almost none in nuclear loci [52]. In our study, the morpho-taxonomic parameters of the selected species were considered for species delimitation. In *Berberis* the phenogram derived from this matrix showed that morphologically the species are well delineated whereas none of the four loci tested could distinguish species of Indian *Berberis*, except *B. pachyacantha*. This prompted us to check the species relationship in *Berberis* and evaluate genetic basis for the delimitation of species using amplified fragment length polymorphism (AFLP). Though the species recovery increased using AFLP method, most of the species remained unresolved. None of the Ahrendt's (1961) sections or subsections in *Berberis* was recognized with the exception of *B. pachyacantha*, which belongs to section ‘vulgaris’. Only one species of section ‘vulgaris’ was included in the present study. It remains to be seen if the species would be resolved when other species of the section are included. Kim *et al*. (2004), in analyzing the ITS phylogeny of *Berberis* species including a few species from India (*B. edgeworthiana, B. insignis, B. coriaria, B. hookeri*) could not recognize the sections and subsections of *Berberis* proposed by Ahrendt (1961). That the species could not be fully resolved using AFLP has been noted before for complexes containing closely related or hybridizing taxa [54]–[57]. There are several reports on occurrence of hybridization in the genus *Berberis* [58]–[60]. The lack of resolution of most of the species e.g. *B. asiatica, B. glaucocarpa, B. lycium* of the section Asiaticae, *B. chitria, B. aristata, B. tinctoria,* and *B. wightiana* of the section Tinctoriae, *B. angulosa* of the section Angulosae and *B. insignis* of the section *Wallichianae* with AFLP data as well as ITS sequences indicates a probable hybridization in these species. This is further corroborated by the fact that in AFLP analysis there was a tendency of the species of *Berberis* to cluster according to their geographic location rather than to species identity. This indicates that, species discrimination seems possible with morphological characters, but reproductive isolation appears to be weak in *Berberis* and in many cases probably only affected by geographical barriers. These findings are consistent with either non-monophyletic or reticulate evolution of these species. Kim *et al* (2004) [25] using ITS sequences of 79 taxa of *Berberis* representing four major groups including Septentrionales and 22 sections in the genus showed that these traditional geographical groups are monophyletic. Therefore, the latter hypothesis is preferred because of evidence of hybridization and relatively young age (5.33-0.01 Ma) of Indian *Berberis* (Pleistocene record of Kashmir, India) [61].

The taxonomic problems described here for Indian *Berberis* are not unique. Similar problems were reported by Spooner (2009) for *Solanum* sect. *Petota* [53]. Hawkes (1990) reported 232 species of sect. *Petota* [62] but Spooner and Salas (2006) reduced it to 190 [63] and more recently, Spooner has converged these to about 110 species [53]. Harlan and de Wet (1971) showed differences in the number of species recognized by different taxonomists in crops, e.g. 100 to 200 in wild relatives of potatoes, 2 to 24 in wheat and 1 to 31 in sorghum [64]. These are some examples where taxonomic disputes still remain unresolved. Plant DNA barcoding in these cases may be problematic and contribute to complexities in search of universal loci for plant DNA barcode.

Barcoding in plant genera like *Berberis* with possible occurrence of natural hybridization and gene introgression may be quite challenging. The morphological, geographical and genomic diversity study in Indian species of *Berberis* indicates probable reticulate nature of the species. Our results with *Ficus* and *Gossypium* suggest that ITS and *trnH-psbA* are good candidates for plant DNA barcoding and the *matK* and *rbcL*, the standard barcode loci for plant barcoding do not work in all the tested species of these three genera.

## Materials and Methods

### Sampling and morphometric analysis

One hundred and sixty four accessions representing 16 species of *Berberis* were collected from four different geographical regions e.g. Eastern Himalayas, Western Himalayas, Central India and Sothern India (Figure 1). Out of these, 3–5 representative accessions from each species were evaluated by morpho-taxonomic analysis. All morphological characters were weighted equally. Multistate characters were unordered. The character matrix thus developed (Table S3) was used to generate the phenograms (Figure S1) with PAUP*4.0b [65]. The parsimony trees were generated using bootstrap analyses with 10,000 replicates. Bootstrap searches were heuristic with simple addition of taxa, TBR branch-swapping and MulTrees turned off. We sampled at least two species from each region except central India where only one species occurs. Thirty three accessions of 11 species of *Ficus* and 51 accessions of 4 species of cultivated *Gossypium* were collected from different parts of India. Multiple accessions were included for each species. Specimen vouchers were deposited in the Herbarium of National Botanical Research Institute, India (LWG). Accession numbers including specimen collection locations are given in Table S10.

### PCR and DNA sequencing

Genomic DNA was extracted from either fresh or silica gel dried leaf materials using DNeasy Plant Mini Kit (Qiagen, Germany) according to manufacturer's instructions. PCR amplification was performed in 50-µl reaction mixtures containing approximately 50–75 ng genomic DNA templates, 1.5 mM MgCl_2_, 0.2 mM of each dNTP, 1 µM of each primer, 0.1 mg BSA/ml and 1 unit *Taq* DNA polymerase. The thermocycler programme was 94°C for 1 min (1 cycle), 94°C for 40 sec, 48°C–52°C (depending upon primer sets used) for 35 cycles, 72°C for 40 sec and 72°C for 5 min (1 cycle). The primers, *matK*2.1a and *matK*3.2r reported by Plant Working Group failed to give PCR amplification in *Berberis* even after changing some PCR conditions including addition of DMSO. A modified *matK* forward primer, *matK-*NBRI (here after referred as *matK*) was designed after aligning the *matK* sequences of genera closely related to *Berberis e.g. Nandina*, *Ranzania*, *Mahonia*. The *matK* along with the reported *matK*3.2r primer was able to successfully amplify in *Berberis*, *Ficus* and *Gossypium*. For primer sequences and references see Table S11. The PCR products were cleaned by Qiaquick® PCR Purification kit (Qiagen, Germany). In a few cases, where multiple bands appeared, these were gel extracted and sequenced. Sequencing was carried out bidirectionally using automated capillary sequencer, ABI3730XL DNA analyzer (Applied Biosystems, UK). Pairwise alignments were made by using the sequences obtained from forward and reverse primers. Sequences which covered more than 70% overlap between forward and reverse sequences were considered (except a few sequence of *matK* where coverage was less than 50%). A minimum average QV of 30 was considered as quality sequences. DNA sequences were edited manually by visual inspection of the electropherograms of both end sequences using Sequencher 4.1.4. The GenBank accession numbers for the sequences are given in Table S10.

Each nrITS sequence was searched in nucleotide data base using BLAST, to confirm its plant origin rather than from a possible fungal contamination of the sample. In all cases the best match retrieved the plant species, either as the same plant species sequence as query sequences or as the nearest plant species (e.g. in most cases sequences of Indian *Berberis* species were not available in data base and *B. thumbergii* showed the best match). Secondly, we looked for the presence of the characteristic conserved motif in the 5.8S rRNA gene of angiosperm plant ITS sequences [66]. The characteristic motif (5′- GAATTGCAGAAT***C***C-3′) was found in all the ITS sequences where as the variant of the motif generally found in fungi (5′-GAATTGCAGAAT***T***C-3′) was not found in any of the sequences.

### AFLP analysis

To establish the species diversity of Indian species of *Berberis* at the genome level, we employed amplified fragment length ploymorphism analysis in 55 accessions of 13 species of *Berberis*. We used the same DNA samples as used in barcode analysis except in few cases where DNA quality was poor for AFLP assays. AFLP protocol was followed as described in user manual (AFLP Plant Mapping Kit, Applied Biosystem, and USA) with minor modifications. Briefly, 0.5–1.0 µg genomic DNA was digested with 10 U *EcoR*I and 10 U *Mse*I in a 20 µL reaction and incubated at 37°C for 5 h. Following 15 min heat inactivation of enzymes, 20 µL of ligation master mix containing 75 pmol each *Mse*I and *EcoR*I adapters with 20 U T4 DNA ligase in 1X T4 DNA ligase buffer was added and incubated overnight at 16°C. The digestion- ligation mixture was diluted with 160 µL sterile water. Pre-selective amplification was performed by using a tri-selective nucleotide (+3) at the 3′. Ten primer combinations were employed to detect polymorphism among different genotypes: *EcoR*I+ AAG/*Mse*I+CAA, *EcoR*I +AAG/*Mse*I+CAT, *EcoR*I+AAC/*Mse*I+CTT,*EcoR*I+AAC/*Mse*I+CTG, *EcoR*I+AGG/*Mse*I+CAA *EcoR*I+AGG/*Mse*I+CTG, *EcoR*I+ACC/*Mse*I+CTC, *EcoR*I+ACC/*Mse*I+CTT, *EcoR*I+ACG/*Mse*I+CAC and *EcoR*I+ ACA/*Mse*I+CTG. The *EcoR*I adapter primers were 5′ fluorescent labeled either with 6-carboxyfluorescein (FAM)/(JOE)/(NED). The *Mse*I adapter primers were unlabeled. Each 25 µL reaction contained 5 µL diluted +1 reaction, 1X PCR buffer, 1.5 1 mM MgCl2, 300 µM dNTP, 4 pmol each Eco RI adapter +3 primer, 25 pmol Mse I 2 adapter +3 primer, and 1 U Taq DNA polymerase. The amplification profile was 94°C for 2 min, 10 cycles of 94°C for 20 s, 66°C for 30 s, 72°C for 2 min, reducing the annealing temperature by 1°C per cycle, followed by 30 cycles of 94°C for 30 s, 56°C for 30 s, 72°C for 2 min, ending with 72°C for 30 min.

### AFLP data analysis

Selective +3 AFLP amplification products were resolved using automated sequencing gels on an ABI Prism1 3730xl DNA Analyzer. Image analysis was performed using GeneMapper version 4.0 (Applied Biosystems,USA), and by visual inspection. Fragments were sized by running dye-labeled standards in each well. GeneMapper automatically scores fragments ranging from 50–500 bp in length. Similarity of fragment size was assumed to indicate homology. Fragment data were recorded as ‘1’ (presence) or ‘0’ (absence) and data entered into binary data matrix as discrete variables. Jaccard's coefficient of similarity was calculated for all pair wise comparison and a dendrogram was made through cluster analysis using the unweighted pair group method based on arithmetic average (UPGMA). The correlation between the Jaccard's similarity and the cophenetic coefficients for the clusters was calculated. Jaccard's coefficient of similarity was used in principal co-ordinate analysis using the DCENTRE and EIGEN functions to resolve pattern of variation among and within species. The relative contribution significance of AFLP bands in species discrimination for the three co-ordinates was analyzed. The NTSYS-pc2.02e was used for all statistical analysis [67].

### Correlation between Geographical and genetic distances

GPS data of samples were converted into a geographical distance matrix using WGS84 (World Geodetic System) model in Geographical Distance Matrix Generator programme [68]. Mantel test was applied to find the correlation between geographical distance matrix and Jaccard's distance coefficient matrix obtained from AFLP data.

### Cloning and sequencing of ITS for copy number detection

The gel eluted PCR fragments (as described above) of ITS sequences of 8 accessions from 8 species of *Berberis* were cloned separately into a pTZ57R/T (Fermentas, USA) TA cloning vector following standard protocol.

These were transformed into *Escherichia coli* DH5α competent cells. Eight colonies were randomly picked up for screening. Plasmid was isolated following standard protocol. Each clone was sequenced using M13 forward and reverse primers as described above.

### Data analyses

The sequences were aligned by ClustalW and the inter- and intraspecific genetic distances were calculated using MEGA4 [69] for each DNA barcode locus. The pair wise distances were calculated with the simplest K2P model implemented in MEGA4. The K2P model considers that transition and transversion happen at different rates and takes into account both transition and transversion rates to calculate the divergence between sequences. These considerations are important as far as variation at inter- and intraspecific level are considered. ANOVA with Bonferroni's Multiple Comparison Test was performed to compare mean inter- and intraspecific variability for each individual pair and all possible multilocus combinations of barcode loci. To exclude inequality of variances, if any, data was log transformed wherever required. The DNA barcoding gaps were evaluated by comparing the minimum interspecific divergence and the maximum intraspecific divergence by t-test. In order to assess the character based approach for barcoding, we first generated haplotype variation from the aligned sequences along with a reference sequence from GenBank data base for each locus using iBarcode web program [70]. Identification and confirmation of unique characters were accomplished in a straight forward manner with respect to the respective reference sequence from the data base by visual inspection. Diagnostic characters were identified using at least three or more accessions per species. Species discrimination power was also calculated using distance approach following CBOL plant working group [17].

### Phylogenetic analysis

To evaluate whether the species were recovered as monophyletic, using the four barcode loci singly or in combinations, we used standard phylogenetic methods. Phylogenetic trees were made with MEGA4 using Neighbor Joining (NJ), Parsimony and Unweighted Pair Group Method with Arithmetic Mean (UPGMA). The NJ and UPGMA trees were built with K2P distance model and 500 bootstrap replicates. MP trees were built with default setting implemented at MEGA4. In all cases indels were treated as complete deletion. In multilocus analysis, 58 DNA sequences representing at least three common accessions were included per species (except in case of *B. angulosa*) for the four loci.

## Supporting Information

Figure S1 Unrooted Bootstrap 50% majority-rule consensus tree of Berberis species. Bootstrap support values are indicated on the nodes. The detailed morphological characters are as described in Table S3.
(0.05 MB PDF)

Figure S2 Strict consensus NJ, MP and UPGMA trees of Berberis species. A) ITS, (B) matK, (C) rbcL and (D) trnH-psbA. Numbers at the branch nodes are bootstrap values. Codes preceding the species name indicate DNA numbers corresponding to the accession numbers analyzed in this study.
(0.12 MB PDF)

Figure S3 Strict consensus NJ, MP and UPGMA trees based on sequences of different multilocus combinations in Berberis. A) ITS+trnH-psbA+matK+rbcL (B) ITS+matK+rbcL (C) ITS+trnH-psbA+rbcL (D) ITS+trnH-psbA+matK (E) trnH-psbA+matK+rbcL (F) ITS+matK (G) ITS+rbcL (H) ITS+trnH-psbA (I) trnH-psbA+matK (J) trnH-psbA+rbcL (K) matK+rbcL. Other details are as in Figure S2.
(0.19 MB PDF)

Figure S4 Strict consensus NJ, MP, UPGMA trees based on 58 common sequences of four loci in Berberis. (A) ITS, (B) matK, (C) rbcL and (D) trnH-psbA. Other details are, as in Figure S2.
(0.09 MB PDF)

Figure S5 Strict consensus NJ, MP, UPGMA trees based on ITS (A), *matK* (B), *rbcL* (C) and *trnH-psbA* (D) sequences of different species of *Ficus*. *F.elastica* and *F.rumphi* were represented by only one accession with *trnH-psbA* sequences.
(0.10 MB PDF)

Figure S6 Strict consensus NJ, MP, UPGMA trees based on ITS (A), *matK* (B), *rbcL* (C) and *trnH-psbA* (D) sequences of different species of *Gossypium*.
(0.08 MB PDF)

Table S1 The classification and distribution of *Berberis* species according toAhrendt(1961). The arrangement of species is alphabetical. The detailed GPS data for distribution of the selected species is given in Table S10.
(0.02 MB PDF)

Table S2 The classification of *Ficus* species according to Corner (1958).
(0.05 MB PDF)

Table S3 The binary matrix developed on the basis of detailed morphological parameters considered for species delineation in *Berberis*. The character matrix developed on the basis of detailed morphological parameters considered for species delineation in Berberis.
(0.09 MB PDF)

Table S4 One Way ANOVA with Bonferroni's multiple comparison tests to compare inter (A) and intraspecific (B) variability for each individual locus in *Berberis*.
(0.06 MB PDF)

Table S5 One way ANOVA with Bonferroni's multiple comparison tests to compare inter (A) and intraspecific (B) variability for each possible multilocus combinations in *Berberis*.
(0.06 MB PDF)

Table S6 Results of paired t-test to compare between minimum inter and maximum intraspecific K2P distances of different loci.
(0.06 MB PDF)

Table S7 Minimum inter- and maximum intraspecific K2P distances of *Berberis* species for different loci and ability to discriminate species. These values could not be calculated in some cases (-) where there was either single accession or sequencing failure for the locus.
(0.08 MB PDF)

Table S8 One way ANOVA with Bonferroni's multiple comparison tests to compare inter (A, *Ficus* and C, *Gossypium*) and intraspecific (B, *Ficus* and D, *Gossypium*) variability for each individual locus.
(0.03 MB PDF)

Table S9 Minimum inter- and maximum intraspecific K2P distances of species of *Ficus* and *Gossypium* for different loci and ability to discriminate species. These values could not be calculated in some cases (-) where there was either single accession or sequencing failure for the locus.
(0.09 MB PDF)

Table S10 List of Accessions and DNA numbers of different plant species along with Global Positioning System (GPS) data, and collector's name. In some cases GPS data could not be taken.
(0.09 MB PDF)

Table S11 Primer sequences used in this study (listed 5′- to 3′).
(0.01 MB PDF)
