# Supplementary figures and images for: Universal Plant DNA Barcode Loci May Not Work in Complex Groups: A Case Study with Indian Berberis Species
Source: PLoS One. 2010 Oct 27;5(10):e13674. doi: 10.1371/journal.pone.0013674 (PMC2965122; doi:10.1371/journal.pone.0013674)

Figure S1

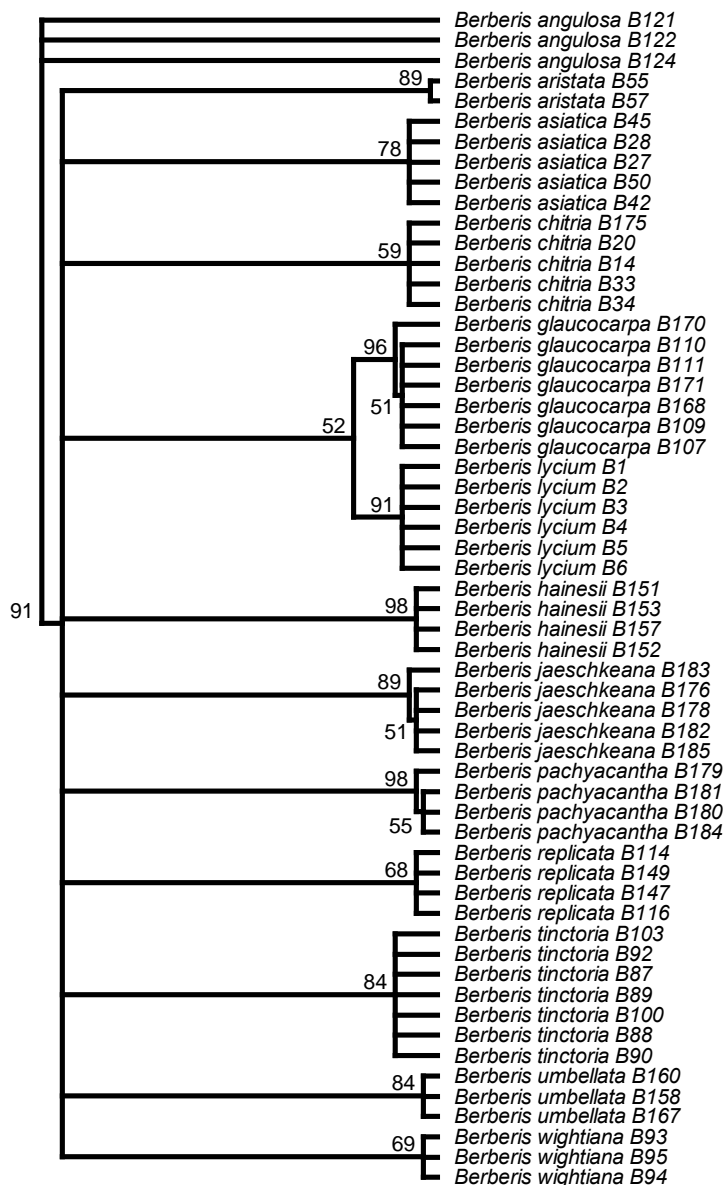

Supplement: Figure S1 — Unrooted Bootstrap 50% majority-rule consensus tree of Berberis species. Bootstrap support values are indicated on the nodes. The detailed morphological characters are as described in Table S3. (0.05 MB PDF) [file pone.0013674.s001.pdf]
