# Supplementary material for: Universal Plant DNA Barcode Loci May Not Work in Complex Groups: A Case Study with Indian Berberis Species
Source: PLoS One. 2010 Oct 27;5(10):e13674. doi: 10.1371/journal.pone.0013674 (PMC2965122; doi:10.1371/journal.pone.0013674)
Supplement: Figure S2 — Strict consensus NJ, MP and UPGMA trees of Berberis species. A) ITS, (B) matK, (C) rbcL and (D) trnH-psbA. Numbers at the branch nodes are bootstrap values. Codes preceding the species name indicate DNA numbers corresponding to the accession numbers analyzed in this study. (0.12 MB PDF) [file pone.0013674.s002.pdf]

(A)

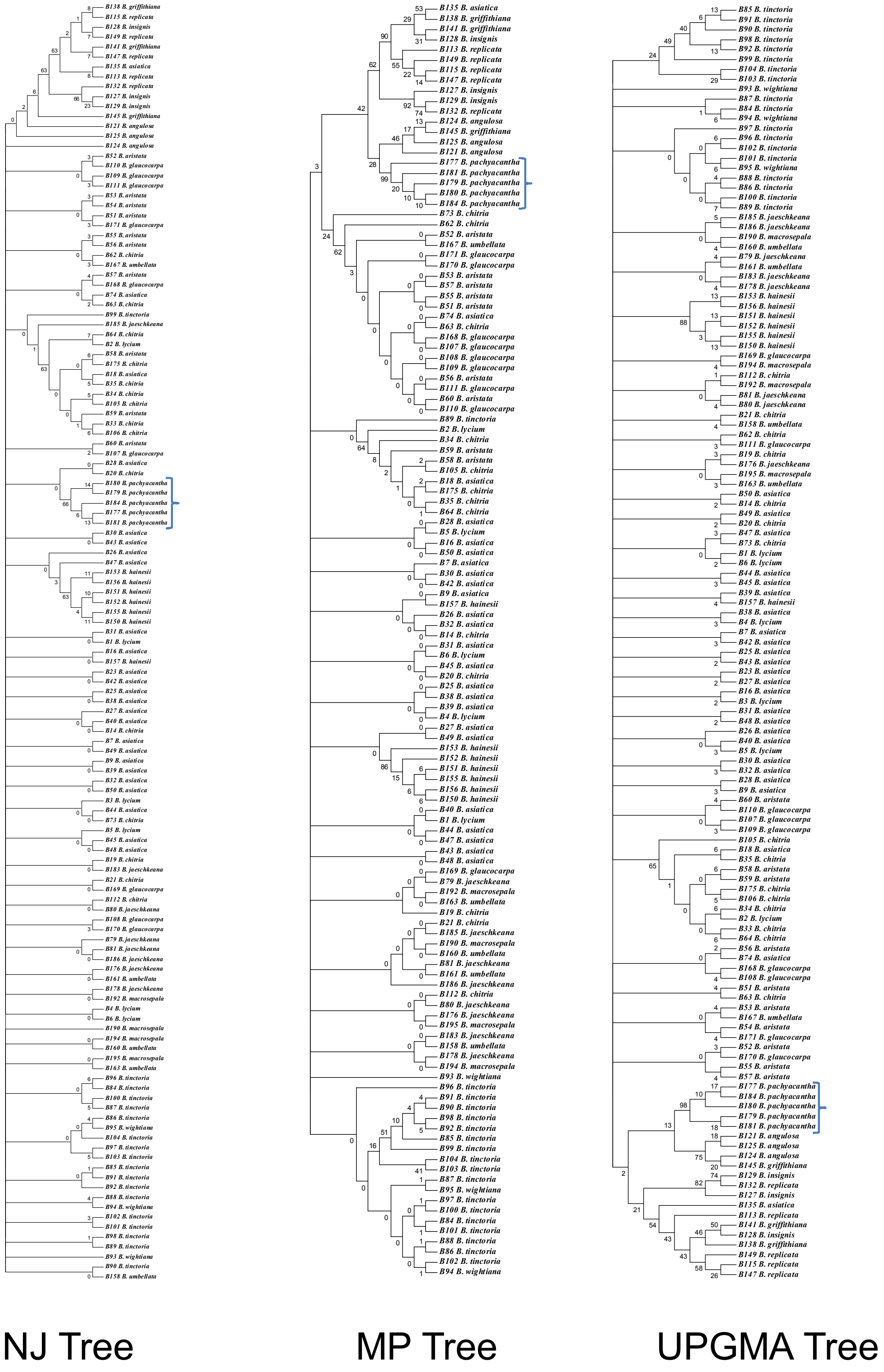

(B)

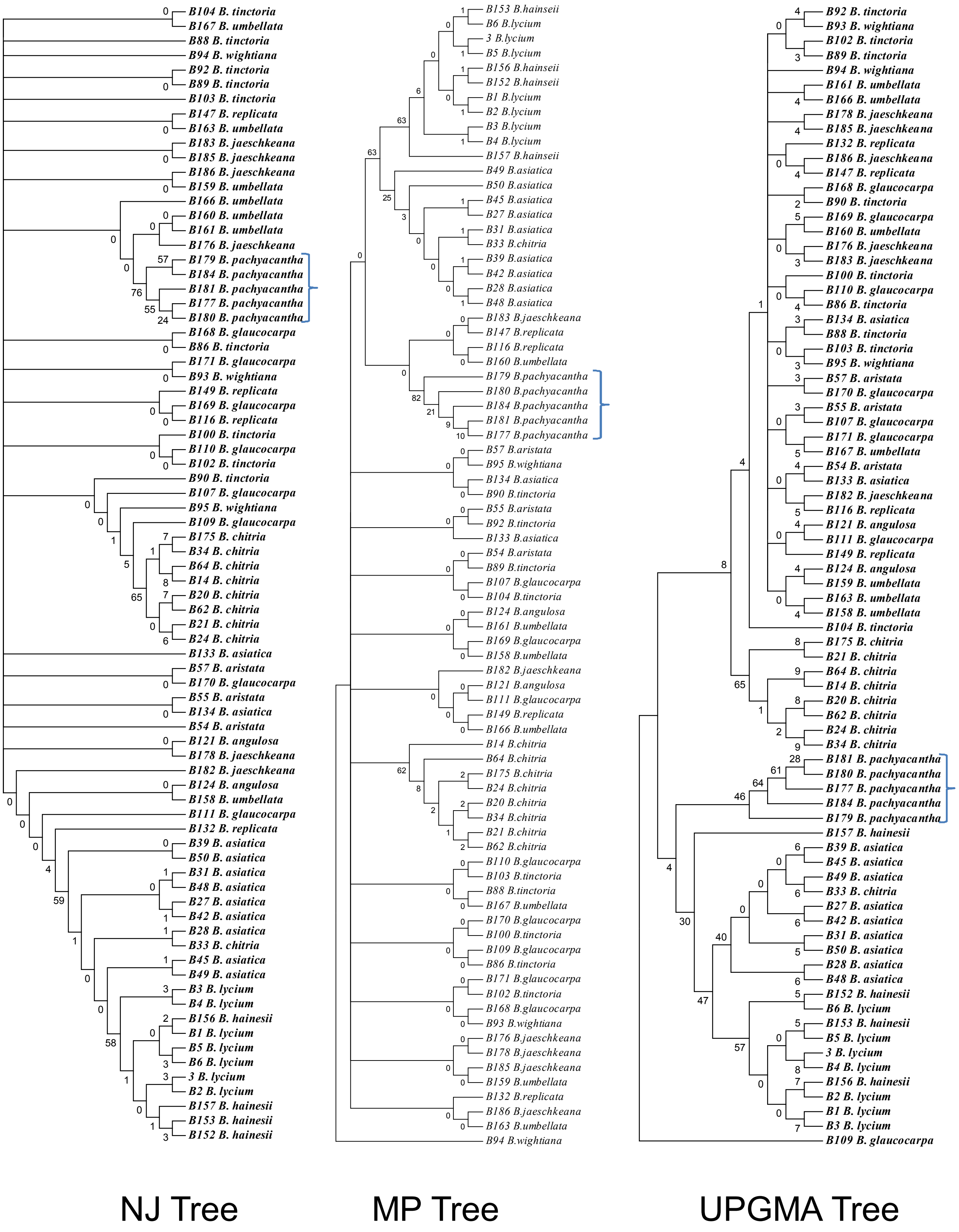
B104 B. tinctoria
B167 B. umbellata
B88 B. tinctoria
B94 B. wightiana
B92 B. tinctoria
B89 B. tinctoria
B103 B. tinctoria
B147 B. replicata
B163 B. umbellata
B183 B. jaeschkeana
B185 B. jaeschkeana
B186 B. jaeschkeana
B159 B. umbellata
B166 B. umbellata
B160 B. umbellata
B161 B. umbellata
B176 B. jaeschkeana
B179 B. pachyacantha
B184 B. pachyacantha
B181 B. pachyacantha
B177 B. pachyacantha
B180 B. pachyacantha
B168 B. glaucocarpa
B86 B. tinctoria
B171 B. glaucocarpa
B93 B. wightiana
B149 B. replicata
B169 B. glaucocarpa
B116 B. replicata
B100 B. tinctoria
B110 B. glaucocarpa
B102 B. tinctoria
B90 B. tinctoria
B107 B. glaucocarpa
B95 B. wightiana
B109 B. glaucocarpa
B175 B. chitria
B34 B. chitria
B64 B. chitria
B14 B. chitria
B20 B. chitria
B62 B. chitria
B21 B. chitria
B24 B. chitria
B133 B. asiatica
B57 B. aristata
B170 B. glaucocarpa
B55 B. aristata
B134 B. asiatica
B54 B. aristata
B121 B. angulosa
B178 B. jaeschkeana
B182 B. jaeschkeana
B124 B. angulosa
B158 B. umbellata
B111 B. glaucocarpa
B132 B. replicata
B39 B. asiatica
B50 B. asiatica
B31 B. asiatica
B48 B. asiatica
B27 B. asiatica
B42 B. asiatica
B28 B. asiatica
B33 B. chitria
B45 B. asiatica
B49 B. asiatica
B3 B. lycium
B4 B. lycium
B156 B. hainesii
B1 B. lycium
B5 B. lycium
B6 B. lycium
3 B. lycium
B2 B. lycium
B157 B. hainesii
B153 B. hainesii
B152 B. hainesii
B153 B.hainseii
B6 B.lycium
3 B.lycium
B5 B.lycium
B156 B.hainseii
B152 B.hainseii
B1 B.lycium
B2 B.lycium
B3 B.lycium
B4 B.lycium
B157 B.hainseii
B49 B.asiatica
B50 B.asiatica
B45 B.asiatica
B27 B.asiatica
B31 B.asiatica
B33 B.chitria
B39 B.asiatica
B42 B.asiatica
B28 B.asiatica
B48 B.asiatica
B183 B.jaeschkeana
B147 B.replicata
B116 B.replicata
B160 B.umbellata
B179 B.pachyacantha
B180 B.pachyacantha
B184 B.pachyacantha
B181 B.pachyacantha
B177 B.pachyacantha
B57 B.aristata
B95 B.wightiana
B134 B.asiatica
B90 B.tinctoria
B55 B.aristata
B92 B.tinctoria
B133 B.asiatica
B54 B.aristata
B89 B.tinctoria
B107 B.glaucocarpa
B104 B.tinctoria
B124 B.angulosa
B161 B.umbellata
B169 B.glaucocarpa
B158 B.umbellata
B182 B.jaeschkeana
B121 B.angulosa
B111 B.glaucocarpa
B149 B.replicata
B166 B.umbellata
B14 B.chitria
B64 B.chitria
B175 B.chitria
B24 B.chitria
B20 B.chitria
B34 B.chitria
B21 B.chitria
B62 B.chitria
B110 B.glaucocarpa
B103 B.tinctoria
B88 B.tinctoria
B167 B.umbellata
B170 B.glaucocarpa
B100 B.tinctoria
B109 B.glaucocarpa
B86 B.tinctoria
B171 B.glaucocarpa
B102 B.tinctoria
B168 B.glaucocarpa
B93 B.wightiana
B176 B.jaeschkeana
B178 B.jaeschkeana
B185 B.jaeschkeana
B159 B.umbellata
B132 B.replicata
B186 B.jaeschkeana
B163 B.umbellata
B94 B.wightiana
B92 B. tinctoria
B93 B. wightiana
B102 B. tinctoria
B89 B. tinctoria
B94 B. wightiana
B161 B. umbellata
B166 B. umbellata
B178 B. jaeschkeana
B185 B. jaeschkeana
B132 B. replicata
B186 B. jaeschkeana
B147 B. replicata
B168 B. glaucocarpa
B90 B. tinctoria
B169 B. glaucocarpa
B160 B. umbellata
B176 B. jaeschkeana
B183 B. jaeschkeana
B100 B. tinctoria
B110 B. glaucocarpa
B86 B. tinctoria
B134 B. asiatica
B88 B. tinctoria
B103 B. tinctoria
B95 B. wightiana
B57 B. aristata
B170 B. glaucocarpa
B55 B. aristata
B107 B. glaucocarpa
B171 B. glaucocarpa
B167 B. umbellata
B54 B. aristata
B133 B. asiatica
B182 B. jaeschkeana
B116 B. replicata
B121 B. angulosa
B111 B. glaucocarpa
B149 B. replicata
B124 B. angulosa
B159 B. umbellata
B163 B. umbellata
B158 B. umbellata
B104 B. tinctoria
B175 B. chitria
B21 B. chitria
B64 B. chitria
B14 B. chitria
B20 B. chitria
B62 B. chitria
B24 B. chitria
B34 B. chitria
B181 B. pachyacantha
B180 B. pachyacantha
B177 B. pachyacantha
B184 B. pachyacantha
B179 B. pachyacantha
B157 B. hainesii
B39 B. asiatica
B45 B. asiatica
B49 B. asiatica
B33 B. chitria
B27 B. asiatica
B42 B. asiatica
B31 B. asiatica
B50 B. asiatica
B28 B. asiatica
B48 B. asiatica
B152 B. hainesii
B6 B. lycium
B153 B. hainesii
B5 B. lycium
3 B. lycium
B4 B. lycium
B156 B. hainesii
B2 B. lycium
B1 B. lycium
B3 B. lycium
B109 B. glaucocarpa
NJ Tree
MP Tree
UPGMA Tree

(C)

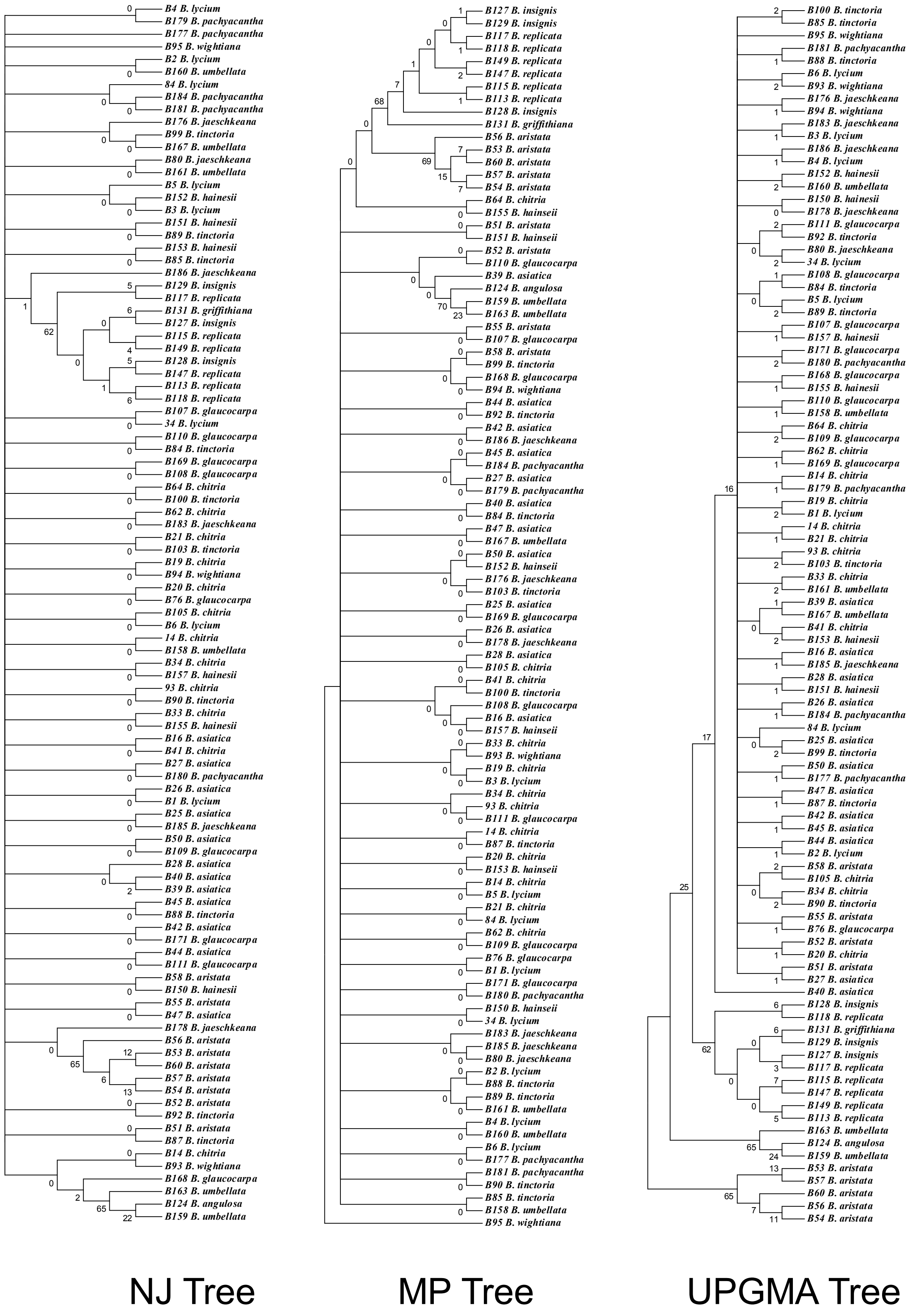
B4 B. lycium
B179 B. pachyacantha
B177 B. pachyacantha
B95 B. wightiana
B2 B. lycium
B160 B. umbellata
84 B. lycium
B184 B. pachyacantha
B181 B. pachyacantha
B176 B. jaeschkeana
B99 B. tinctoria
B167 B. umbellata
B80 B. jaeschkeana
B161 B. umbellata
B5 B. lycium
B152 B. hainesii
B3 B. lycium
B151 B. hainesii
B89 B. tinctoria
B153 B. hainesii
B85 B. tinctoria
B186 B. jaeschkeana
B129 B. insignis
B117 B. replicata
B131 B. griffithiana
B127 B. insignis
B115 B. replicata
B149 B. replicata
B128 B. insignis
B147 B. replicata
B113 B. replicata
B118 B. replicata
B107 B. glaucocarpa
34 B. lycium
B110 B. glaucocarpa
B84 B. tinctoria
B169 B. glaucocarpa
B108 B. glaucocarpa
B64 B. chitria
B100 B. tinctoria
B62 B. chitria
B183 B. jaeschkeana
B21 B. chitria
B103 B. tinctoria
B19 B. chitria
B94 B. wightiana
B20 B. chitria
B76 B. glaucocarpa
B105 B. chitria
B6 B. lycium
14 B. chitria
B158 B. umbellata
B34 B. chitria
B157 B. hainesii
93 B. chitria
B90 B. tinctoria
B33 B. chitria
B155 B. hainesii
B16 B. asiatica
B41 B. chitria
B27 B. asiatica
B180 B. pachyacantha
B26 B. asiatica
B1 B. lycium
B25 B. asiatica
B185 B. jaeschkeana
B50 B. asiatica
B109 B. glaucocarpa
B28 B. asiatica
B40 B. asiatica
B39 B. asiatica
B45 B. asiatica
B88 B. tinctoria
B42 B. asiatica
B171 B. glaucocarpa
B44 B. asiatica
B111 B. glaucocarpa
B58 B. aristata
B150 B. hainesii
B55 B. aristata
B47 B. asiatica
B178 B. jaeschkeana
B56 B. aristata
B53 B. aristata
B60 B. aristata
B57 B. aristata
B54 B. aristata
B52 B. aristata
B92 B. tinctoria
B51 B. aristata
B87 B. tinctoria
B14 B. chitria
B93 B. wightiana
B168 B. glaucocarpa
B163 B. umbellata
B124 B. angulosa
B159 B. umbellata
B127 B. insignis
B129 B. insignis
B117 B. replicata
B118 B. replicata
B149 B. replicata
B147 B. replicata
B115 B. replicata
B113 B. replicata
B128 B. insignis
B131 B. griffithiana
B56 B. aristata
B53 B. aristata
B60 B. aristata
B57 B. aristata
B54 B. aristata
B64 B. chitria
B155 B. hainseii
B51 B. aristata
B151 B. hainseii
B52 B. aristata
B110 B. glaucocarpa
B39 B. asiatica
B124 B. angulosa
B159 B. umbellata
B163 B. umbellata
B55 B. aristata
B107 B. glaucocarpa
B58 B. aristata
B99 B. tinctoria
B168 B. glaucocarpa
B94 B. wightiana
B44 B. asiatica
B92 B. tinctoria
B42 B. asiatica
B186 B. jaeschkeana
B45 B. asiatica
B184 B. pachyacantha
B27 B. asiatica
B179 B. pachyacantha
B40 B. asiatica
B84 B. tinctoria
B47 B. asiatica
B167 B. umbellata
B50 B. asiatica
B152 B. hainseii
B176 B. jaeschkeana
B103 B. tinctoria
B25 B. asiatica
B169 B. glaucocarpa
B26 B. asiatica
B178 B. jaeschkeana
B28 B. asiatica
B105 B. chitria
B41 B. chitria
B100 B. tinctoria
B108 B. glaucocarpa
B16 B. asiatica
B157 B. hainseii
B33 B. chitria
B93 B. wightiana
B19 B. chitria
B3 B. lycium
B34 B. chitria
93 B. chitria
B111 B. glaucocarpa
14 B. chitria
B87 B. tinctoria
B20 B. chitria
B153 B. hainseii
B14 B. chitria
B5 B. lycium
B21 B. chitria
84 B. lycium
B62 B. chitria
B109 B. glaucocarpa
B76 B. glaucocarpa
B1 B. lycium
B171 B. glaucocarpa
B180 B. pachyacantha
B150 B. hainseii
34 B. lycium
B183 B. jaeschkeana
B185 B. jaeschkeana
B80 B. jaeschkeana
B2 B. lycium
B88 B. tinctoria
B89 B. tinctoria
B161 B. umbellata
B4 B. lycium
B160 B. umbellata
B6 B. lycium
B177 B. pachyacantha
B181 B. pachyacantha
B90 B. tinctoria
B85 B. tinctoria
B158 B. umbellata
B95 B. wightiana
B100 B. tinctoria
B85 B. tinctoria
B95 B. wightiana
B181 B. pachyacantha
B88 B. tinctoria
B6 B. lycium
B93 B. wightiana
B176 B. jaeschkeana
B94 B. wightiana
B183 B. jaeschkeana
B3 B. lycium
B186 B. jaeschkeana
B4 B. lycium
B152 B. hainesii
B160 B. umbellata
B150 B. hainesii
B178 B. jaeschkeana
B111 B. glaucocarpa
B92 B. tinctoria
B80 B. jaeschkeana
34 B. lycium
B108 B. glaucocarpa
B84 B. tinctoria
B5 B. lycium
B89 B. tinctoria
B107 B. glaucocarpa
B157 B. hainesii
B171 B. glaucocarpa
B180 B. pachyacantha
B168 B. glaucocarpa
B155 B. hainesii
B110 B. glaucocarpa
B158 B. umbellata
B64 B. chitria
B109 B. glaucocarpa
B62 B. chitria
B169 B. glaucocarpa
B14 B. chitria
B179 B. pachyacantha
B19 B. chitria
B1 B. lycium
14 B. chitria
B21 B. chitria
93 B. chitria
B103 B. tinctoria
B33 B. chitria
B161 B. umbellata
B39 B. asiatica
B167 B. umbellata
B41 B. chitria
B153 B. hainesii
B16 B. asiatica
B185 B. jaeschkeana
B28 B. asiatica
B151 B. hainesii
B26 B. asiatica
B184 B. pachyacantha
84 B. lycium
B25 B. asiatica
B99 B. tinctoria
B50 B. asiatica
B177 B. pachyacantha
B47 B. asiatica
B87 B. tinctoria
B42 B. asiatica
B45 B. asiatica
B44 B. asiatica
B2 B. lycium
B58 B. aristata
B105 B. chitria
B34 B. chitria
B90 B. tinctoria
B55 B. aristata
B76 B. glaucocarpa
B52 B. aristata
B20 B. chitria
B51 B. aristata
B27 B. asiatica
B40 B. asiatica
B128 B. insignis
B118 B. replicata
B131 B. griffithiana
B129 B. insignis
B127 B. insignis
B117 B. replicata
B115 B. replicata
B147 B. replicata
B149 B. replicata
B113 B. replicata
B163 B. umbellata
B124 B. angulosa
B159 B. umbellata
B53 B. aristata
B57 B. aristata
B60 B. aristata
B56 B. aristata
B54 B. aristata
NJ Tree
MP Tree
UPGMA Tree

(D)

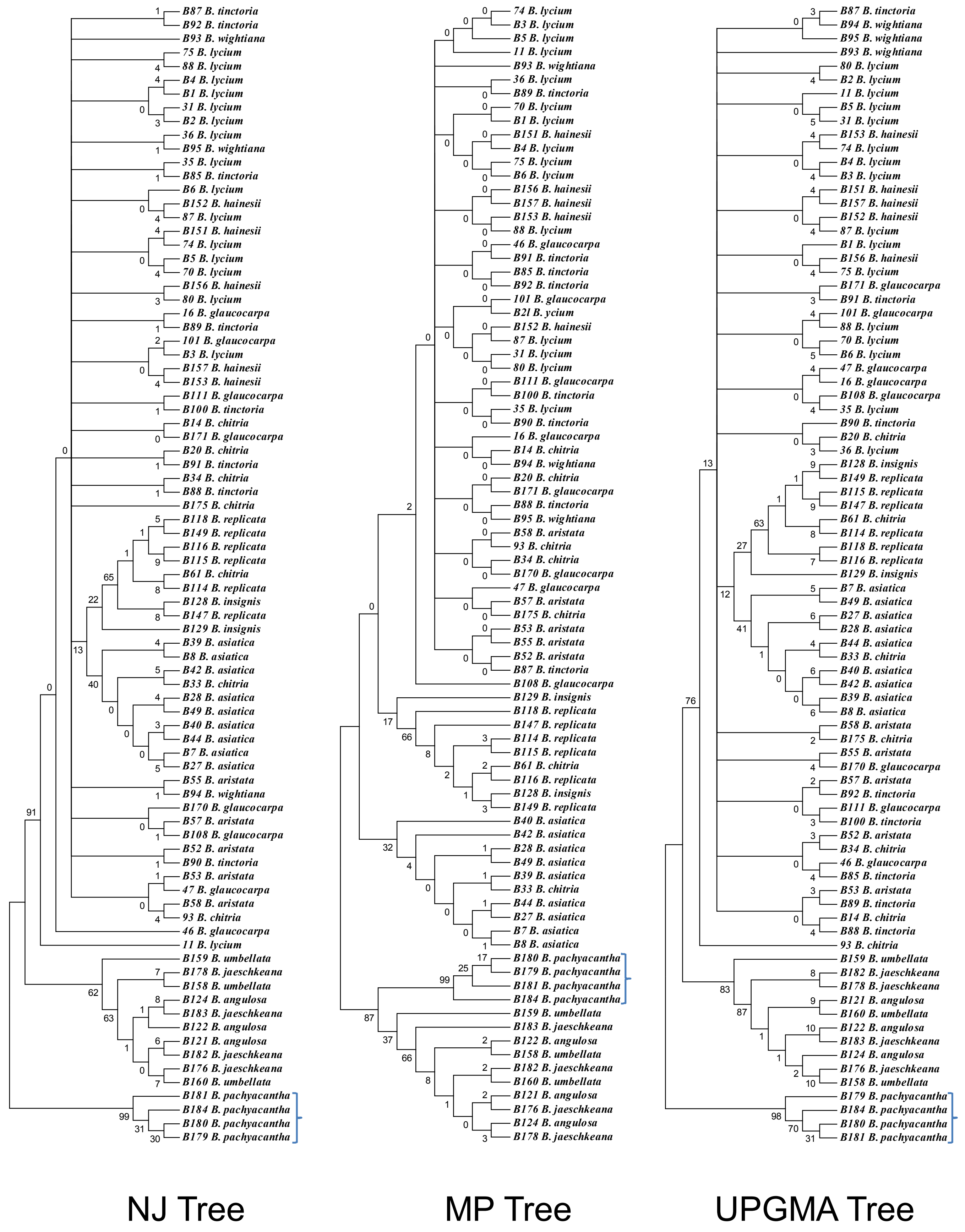
B87 B. tinctoria
B92 B. tinctoria
B93 B. wightiana
75 B. lycium
88 B. lycium
B4 B. lycium
B1 B. lycium
31 B. lycium
B2 B. lycium
36 B. lycium
B95 B. wightiana
35 B. lycium
B85 B. tinctoria
B6 B. lycium
B152 B. hainesii
87 B. lycium
B151 B. hainesii
74 B. lycium
B5 B. lycium
70 B. lycium
B156 B. hainesii
80 B. lycium
16 B. glaucocarpa
B89 B. tinctoria
101 B. glaucocarpa
B3 B. lycium
B157 B. hainesii
B153 B. hainesii
B111 B. glaucocarpa
B100 B. tinctoria
B14 B. chitria
B171 B. glaucocarpa
B20 B. chitria
B91 B. tinctoria
B34 B. chitria
B88 B. tinctoria
B175 B. chitria
B118 B. replicata
B149 B. replicata
B116 B. replicata
B115 B. replicata
B61 B. chitria
B114 B. replicata
B128 B. insignis
B147 B. replicata
B129 B. insignis
B39 B. asiatica
B8 B. asiatica
B42 B. asiatica
B33 B. chitria
B28 B. asiatica
B49 B. asiatica
B40 B. asiatica
B44 B. asiatica
B7 B. asiatica
B27 B. asiatica
B55 B. aristata
B94 B. wightiana
B170 B. glaucocarpa
B57 B. aristata
B108 B. glaucocarpa
B52 B. aristata
B90 B. tinctoria
B53 B. aristata
47 B. glaucocarpa
B58 B. aristata
93 B. chitria
46 B. glaucocarpa
11 B. lycium
B159 B. umbellata
B178 B. jaeschkeana
B158 B. umbellata
B124 B. angulosa
B183 B. jaeschkeana
B122 B. angulosa
B121 B. angulosa
B182 B. jaeschkeana
B176 B. jaeschkeana
B160 B. umbellata
B181 B. pachyacantha
B184 B. pachyacantha
B180 B. pachyacantha
B179 B. pachyacantha
74 B. lycium
B3 B. lycium
B5 B. lycium
11 B. lycium
B93 B. wightiana
36 B. lycium
B89 B. tinctoria
70 B. lycium
B1 B. lycium
B151 B. hainesii
B4 B. lycium
75 B. lycium
B6 B. lycium
B156 B. hainesii
B157 B. hainesii
B153 B. hainesii
88 B. lycium
46 B. glaucocarpa
B91 B. tinctoria
B85 B. tinctoria
B92 B. tinctoria
101 B. glaucocarpa
B21 B. ycium
B152 B. hainesii
87 B. lycium
31 B. lycium
80 B. lycium
B111 B. glaucocarpa
B100 B. tinctoria
35 B. lycium
B90 B. tinctoria
16 B. glaucocarpa
B14 B. chitria
B94 B. wightiana
B20 B. chitria
B171 B. glaucocarpa
B88 B. tinctoria
B95 B. wightiana
B58 B. aristata
93 B. chitria
B34 B. chitria
B170 B. glaucocarpa
47 B. glaucocarpa
B57 B. aristata
B175 B. chitria
B53 B. aristata
B55 B. aristata
B52 B. aristata
B87 B. tinctoria
B108 B. glaucocarpa
B129 B. insignis
B118 B. replicata
B147 B. replicata
B114 B. replicata
B115 B. replicata
B61 B. chitria
B116 B. replicata
B128 B. insignis
B149 B. replicata
B40 B. asiatica
B42 B. asiatica
B28 B. asiatica
B49 B. asiatica
B39 B. asiatica
B33 B. chitria
B44 B. asiatica
B27 B. asiatica
B7 B. asiatica
B8 B. asiatica
B180 B. pachyacantha
B179 B. pachyacantha
B181 B. pachyacantha
B184 B. pachyacantha
B159 B. umbellata
B183 B. jaeschkeana
B122 B. angulosa
B158 B. umbellata
B182 B. jaeschkeana
B160 B. umbellata
B121 B. angulosa
B176 B. jaeschkeana
B124 B. angulosa
B178 B. jaeschkeana
B87 B. tinctoria
B94 B. wightiana
B95 B. wightiana
B93 B. wightiana
80 B. lycium
B2 B. lycium
11 B. lycium
B5 B. lycium
31 B. lycium
B153 B. hainesii
74 B. lycium
B4 B. lycium
B3 B. lycium
B151 B. hainesii
B157 B. hainesii
B152 B. hainesii
87 B. lycium
B1 B. lycium
B156 B. hainesii
75 B. lycium
B171 B. glaucocarpa
B91 B. tinctoria
101 B. glaucocarpa
88 B. lycium
70 B. lycium
B6 B. lycium
47 B. glaucocarpa
16 B. glaucocarpa
B108 B. glaucocarpa
35 B. lycium
B90 B. tinctoria
B20 B. chitria
36 B. lycium
B128 B. insignis
B149 B. replicata
B115 B. replicata
B147 B. replicata
B61 B. chitria
B114 B. replicata
B118 B. replicata
B116 B. replicata
B129 B. insignis
B7 B. asiatica
B49 B. asiatica
B27 B. asiatica
B28 B. asiatica
B44 B. asiatica
B33 B. chitria
B40 B. asiatica
B42 B. asiatica
B39 B. asiatica
B8 B. asiatica
B58 B. aristata
B175 B. chitria
B55 B. aristata
B170 B. glaucocarpa
B57 B. aristata
B92 B. tinctoria
B111 B. glaucocarpa
B100 B. tinctoria
B52 B. aristata
B34 B. chitria
46 B. glaucocarpa
B85 B. tinctoria
B53 B. aristata
B89 B. tinctoria
B14 B. chitria
B88 B. tinctoria
93 B. chitria
B159 B. umbellata
B182 B. jaeschkeana
B178 B. jaeschkeana
B121 B. angulosa
B160 B. umbellata
B122 B. angulosa
B183 B. jaeschkeana
B124 B. angulosa
B176 B. jaeschkeana
B158 B. umbellata
B179 B. pachyacantha
B184 B. pachyacantha
B180 B. pachyacantha
B181 B. pachyacantha
NJ Tree
MP Tree
UPGMA Tree
