# Supplementary material for: Universal Plant DNA Barcode Loci May Not Work in Complex Groups: A Case Study with Indian Berberis Species
Source: PLoS One. 2010 Oct 27;5(10):e13674. doi: 10.1371/journal.pone.0013674 (PMC2965122; doi:10.1371/journal.pone.0013674)
Supplement: Figure S3 — Strict consensus NJ, MP and UPGMA trees based on sequences of different multilocus combinations in Berberis. A) ITS+trnH-psbA+matK+rbcL (B) ITS+matK+rbcL (C) ITS+trnH-psbA+rbcL (D) ITS+trnH-psbA+matK (E) trnH-psbA+matK+rbcL (F) ITS+matK (G) ITS+rbcL (H) ITS+trnH-psbA (I) trnH-psbA+matK (J) trnH-psbA+rbcL (K) matK+rbcL. Other details are as in Figure S2. (0.19 MB PDF) [file pone.0013674.s003.pdf]

(A)

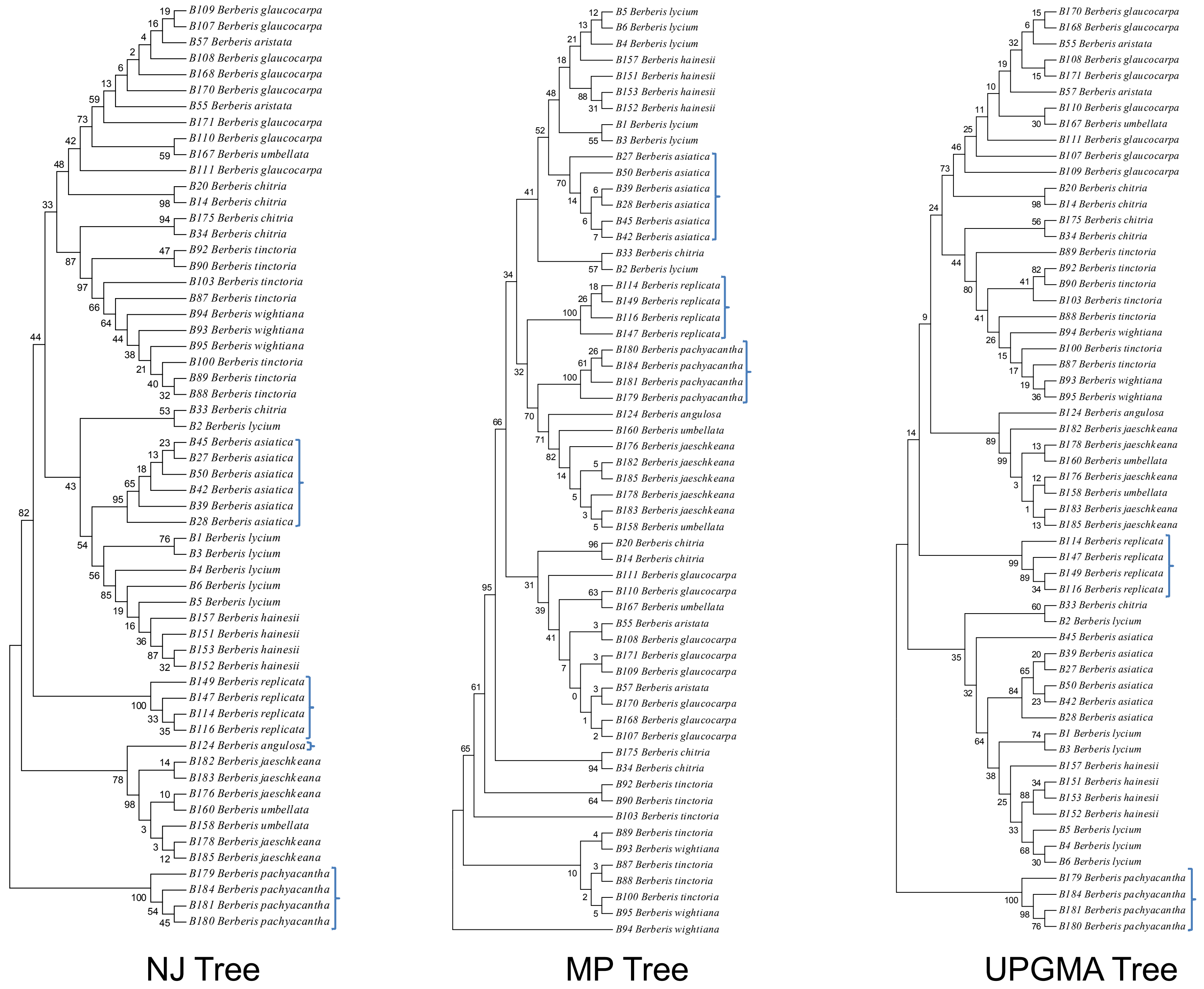
B109 Berberis glaucocarpa
B107 Berberis glaucocarpa
B57 Berberis aristata
B108 Berberis glaucocarpa
B168 Berberis glaucocarpa
B170 Berberis glaucocarpa
B55 Berberis aristata
B171 Berberis glaucocarpa
B110 Berberis glaucocarpa
B167 Berberis umbellata
B111 Berberis glaucocarpa
B20 Berberis chitria
B14 Berberis chitria
B175 Berberis chitria
B34 Berberis chitria
B92 Berberis tinctoria
B90 Berberis tinctoria
B103 Berberis tinctoria
B87 Berberis tinctoria
B94 Berberis wightiana
B93 Berberis wightiana
B95 Berberis wightiana
B100 Berberis tinctoria
B89 Berberis tinctoria
B88 Berberis tinctoria
B33 Berberis chitria
B2 Berberis lycium
B45 Berberis asiatica
B27 Berberis asiatica
B50 Berberis asiatica
B42 Berberis asiatica
B39 Berberis asiatica
B28 Berberis asiatica
B1 Berberis lycium
B3 Berberis lycium
B4 Berberis lycium
B6 Berberis lycium
B5 Berberis lycium
B157 Berberis hainesii
B151 Berberis hainesii
B153 Berberis hainesii
B152 Berberis hainesii
B149 Berberis replicata
B147 Berberis replicata
B114 Berberis replicata
B116 Berberis replicata
B124 Berberis angulosa
B182 Berberis jaeschkeana
B183 Berberis jaeschkeana
B176 Berberis jaeschkeana
B160 Berberis umbellata
B158 Berberis umbellata
B178 Berberis jaeschkeana
B185 Berberis jaeschkeana
B179 Berberis pachyacantha
B184 Berberis pachyacantha
B181 Berberis pachyacantha
B180 Berberis pachyacantha
NJ Tree
B5 Berberis lycium
B6 Berberis lycium
B4 Berberis lycium
B157 Berberis hainesii
B151 Berberis hainesii
B153 Berberis hainesii
B152 Berberis hainesii
B1 Berberis lycium
B3 Berberis lycium
B27 Berberis asiatica
B50 Berberis asiatica
B39 Berberis asiatica
B28 Berberis asiatica
B45 Berberis asiatica
B42 Berberis asiatica
B33 Berberis chitria
B2 Berberis lycium
B114 Berberis replicata
B149 Berberis replicata
B116 Berberis replicata
B147 Berberis replicata
B180 Berberis pachyacantha
B184 Berberis pachyacantha
B181 Berberis pachyacantha
B179 Berberis pachyacantha
B124 Berberis angulosa
B160 Berberis umbellata
B176 Berberis jaeschkeana
B182 Berberis jaeschkeana
B185 Berberis jaeschkeana
B178 Berberis jaeschkeana
B183 Berberis jaeschkeana
B158 Berberis umbellata
B20 Berberis chitria
B14 Berberis chitria
B111 Berberis glaucocarpa
B110 Berberis glaucocarpa
B167 Berberis umbellata
B55 Berberis aristata
B108 Berberis glaucocarpa
B171 Berberis glaucocarpa
B109 Berberis glaucocarpa
B57 Berberis aristata
B170 Berberis glaucocarpa
B168 Berberis glaucocarpa
B107 Berberis glaucocarpa
B175 Berberis chitria
B34 Berberis chitria
B92 Berberis tinctoria
B90 Berberis tinctoria
B103 Berberis tinctoria
B89 Berberis tinctoria
B93 Berberis wightiana
B87 Berberis tinctoria
B88 Berberis tinctoria
B100 Berberis tinctoria
B95 Berberis wightiana
B94 Berberis wightiana
MP Tree
B170 Berberis glaucocarpa
B168 Berberis glaucocarpa
B55 Berberis aristata
B108 Berberis glaucocarpa
B171 Berberis glaucocarpa
B57 Berberis aristata
B110 Berberis glaucocarpa
B167 Berberis umbellata
B111 Berberis glaucocarpa
B107 Berberis glaucocarpa
B109 Berberis glaucocarpa
B20 Berberis chitria
B14 Berberis chitria
B175 Berberis chitria
B34 Berberis chitria
B89 Berberis tinctoria
B92 Berberis tinctoria
B90 Berberis tinctoria
B103 Berberis tinctoria
B88 Berberis tinctoria
B94 Berberis wightiana
B100 Berberis tinctoria
B87 Berberis tinctoria
B93 Berberis wightiana
B95 Berberis wightiana
B124 Berberis angulosa
B182 Berberis jaeschkeana
B178 Berberis jaeschkeana
B160 Berberis umbellata
B176 Berberis jaeschkeana
B158 Berberis umbellata
B183 Berberis jaeschkeana
B185 Berberis jaeschkeana
B114 Berberis replicata
B147 Berberis replicata
B149 Berberis replicata
B116 Berberis replicata
B33 Berberis chitria
B2 Berberis lycium
B45 Berberis asiatica
B39 Berberis asiatica
B27 Berberis asiatica
B50 Berberis asiatica
B42 Berberis asiatica
B28 Berberis asiatica
B1 Berberis lycium
B3 Berberis lycium
B157 Berberis hainesii
B151 Berberis hainesii
B153 Berberis hainesii
B152 Berberis hainesii
B5 Berberis lycium
B4 Berberis lycium
B6 Berberis lycium
B179 Berberis pachyacantha
B184 Berberis pachyacantha
B181 Berberis pachyacantha
B180 Berberis pachyacantha
UPGMA Tree

(B)

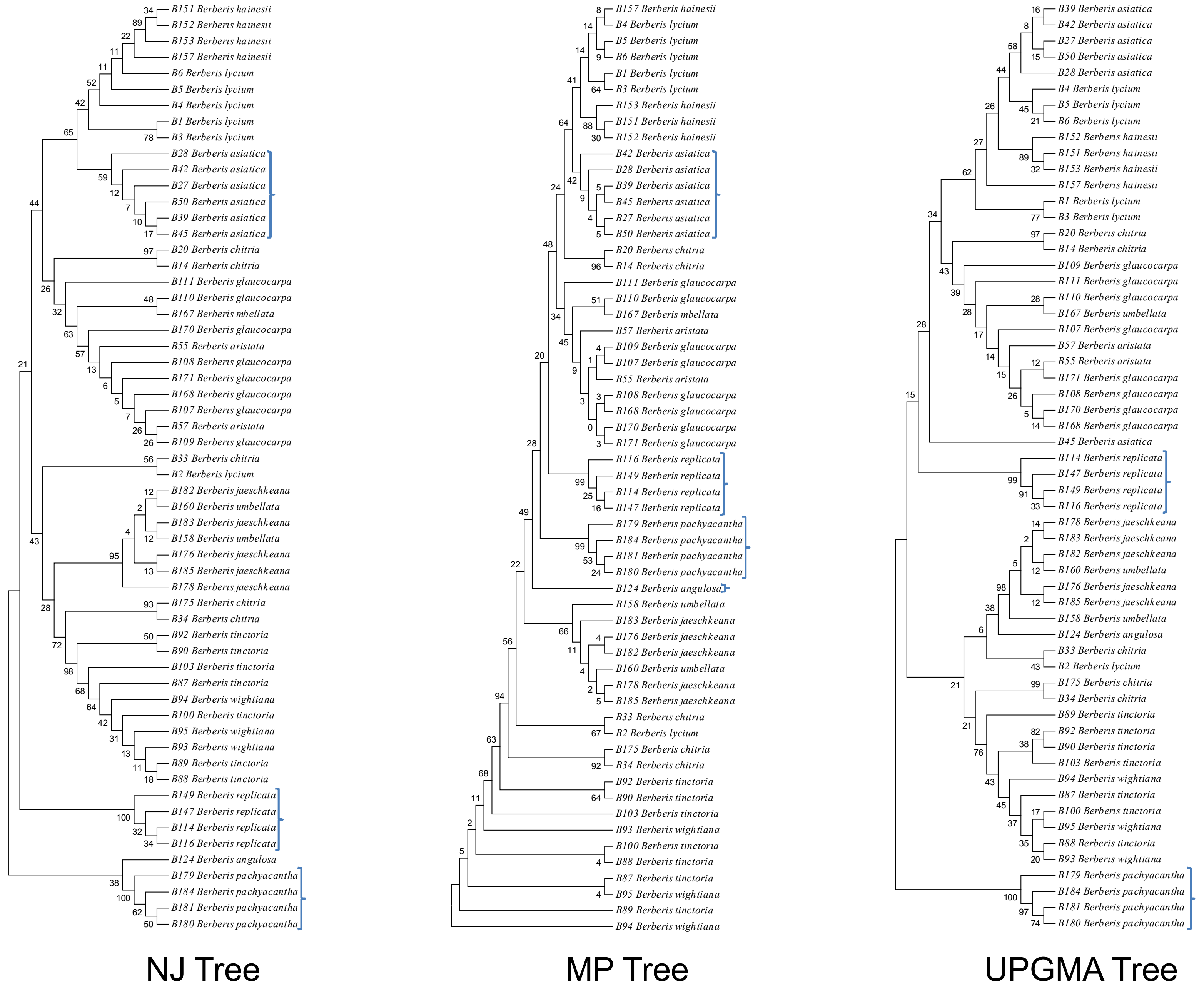
B151 Berberis hainesii
B152 Berberis hainesii
B153 Berberis hainesii
B157 Berberis hainesii
B6 Berberis lycium
B5 Berberis lycium
B4 Berberis lycium
B1 Berberis lycium
B3 Berberis lycium
B28 Berberis asiatica
B42 Berberis asiatica
B27 Berberis asiatica
B50 Berberis asiatica
B39 Berberis asiatica
B45 Berberis asiatica
B20 Berberis chitria
B14 Berberis chitria
B111 Berberis glaucocarpa
B110 Berberis glaucocarpa
B167 Berberis mbellata
B170 Berberis glaucocarpa
B55 Berberis aristata
B108 Berberis glaucocarpa
B171 Berberis glaucocarpa
B168 Berberis glaucocarpa
B107 Berberis glaucocarpa
B57 Berberis aristata
B109 Berberis glaucocarpa
B33 Berberis chitria
B2 Berberis lycium
B182 Berberis jaeschkeana
B160 Berberis umbellata
B183 Berberis jaeschkeana
B158 Berberis umbellata
B176 Berberis jaeschkeana
B185 Berberis jaeschkeana
B178 Berberis jaeschkeana
B175 Berberis chitria
B34 Berberis chitria
B92 Berberis tinctoria
B90 Berberis tinctoria
B103 Berberis tinctoria
B87 Berberis tinctoria
B94 Berberis wightiana
B100 Berberis tinctoria
B95 Berberis wightiana
B93 Berberis wightiana
B89 Berberis tinctoria
B88 Berberis tinctoria
B149 Berberis replicata
B147 Berberis replicata
B114 Berberis replicata
B116 Berberis replicata
B124 Berberis angulosa
B179 Berberis pachyacantha
B184 Berberis pachyacantha
B181 Berberis pachyacantha
B180 Berberis pachyacantha
NJ Tree
B157 Berberis hainesii
B4 Berberis lycium
B5 Berberis lycium
B6 Berberis lycium
B1 Berberis lycium
B3 Berberis lycium
B153 Berberis hainesii
B151 Berberis hainesii
B152 Berberis hainesii
B42 Berberis asiatica
B28 Berberis asiatica
B39 Berberis asiatica
B45 Berberis asiatica
B27 Berberis asiatica
B50 Berberis asiatica
B20 Berberis chitria
B14 Berberis chitria
B111 Berberis glaucocarpa
B110 Berberis glaucocarpa
B167 Berberis mbellata
B57 Berberis aristata
B109 Berberis glaucocarpa
B107 Berberis glaucocarpa
B55 Berberis aristata
B108 Berberis glaucocarpa
B168 Berberis glaucocarpa
B170 Berberis glaucocarpa
B171 Berberis glaucocarpa
B116 Berberis replicata
B149 Berberis replicata
B114 Berberis replicata
B147 Berberis replicata
B179 Berberis pachyacantha
B184 Berberis pachyacantha
B181 Berberis pachyacantha
B180 Berberis pachyacantha
B124 Berberis angulosa
B158 Berberis umbellata
B183 Berberis jaeschkeana
B176 Berberis jaeschkeana
B182 Berberis jaeschkeana
B160 Berberis umbellata
B178 Berberis jaeschkeana
B185 Berberis jaeschkeana
B33 Berberis chitria
B2 Berberis lycium
B175 Berberis chitria
B34 Berberis chitria
B92 Berberis tinctoria
B90 Berberis tinctoria
B103 Berberis tinctoria
B93 Berberis wightiana
B100 Berberis tinctoria
B88 Berberis tinctoria
B87 Berberis tinctoria
B95 Berberis wightiana
B89 Berberis tinctoria
B94 Berberis wightiana
MP Tree
B39 Berberis asiatica
B42 Berberis asiatica
B27 Berberis asiatica
B50 Berberis asiatica
B28 Berberis asiatica
B4 Berberis lycium
B5 Berberis lycium
B6 Berberis lycium
B152 Berberis hainesii
B151 Berberis hainesii
B153 Berberis hainesii
B157 Berberis hainesii
B1 Berberis lycium
B3 Berberis lycium
B20 Berberis chitria
B14 Berberis chitria
B109 Berberis glaucocarpa
B111 Berberis glaucocarpa
B110 Berberis glaucocarpa
B167 Berberis umbellata
B107 Berberis glaucocarpa
B57 Berberis aristata
B55 Berberis aristata
B171 Berberis glaucocarpa
B108 Berberis glaucocarpa
B170 Berberis glaucocarpa
B168 Berberis glaucocarpa
B45 Berberis asiatica
B114 Berberis replicata
B147 Berberis replicata
B149 Berberis replicata
B116 Berberis replicata
B178 Berberis jaeschkeana
B183 Berberis jaeschkeana
B182 Berberis jaeschkeana
B160 Berberis umbellata
B176 Berberis jaeschkeana
B185 Berberis jaeschkeana
B158 Berberis umbellata
B124 Berberis angulosa
B33 Berberis chitria
B2 Berberis lycium
B175 Berberis chitria
B34 Berberis chitria
B89 Berberis tinctoria
B92 Berberis tinctoria
B90 Berberis tinctoria
B103 Berberis tinctoria
B94 Berberis wightiana
B87 Berberis tinctoria
B100 Berberis tinctoria
B95 Berberis wightiana
B88 Berberis tinctoria
B93 Berberis wightiana
B179 Berberis pachyacantha
B184 Berberis pachyacantha
B181 Berberis pachyacantha
B180 Berberis pachyacantha
UPGMA Tree

(C)

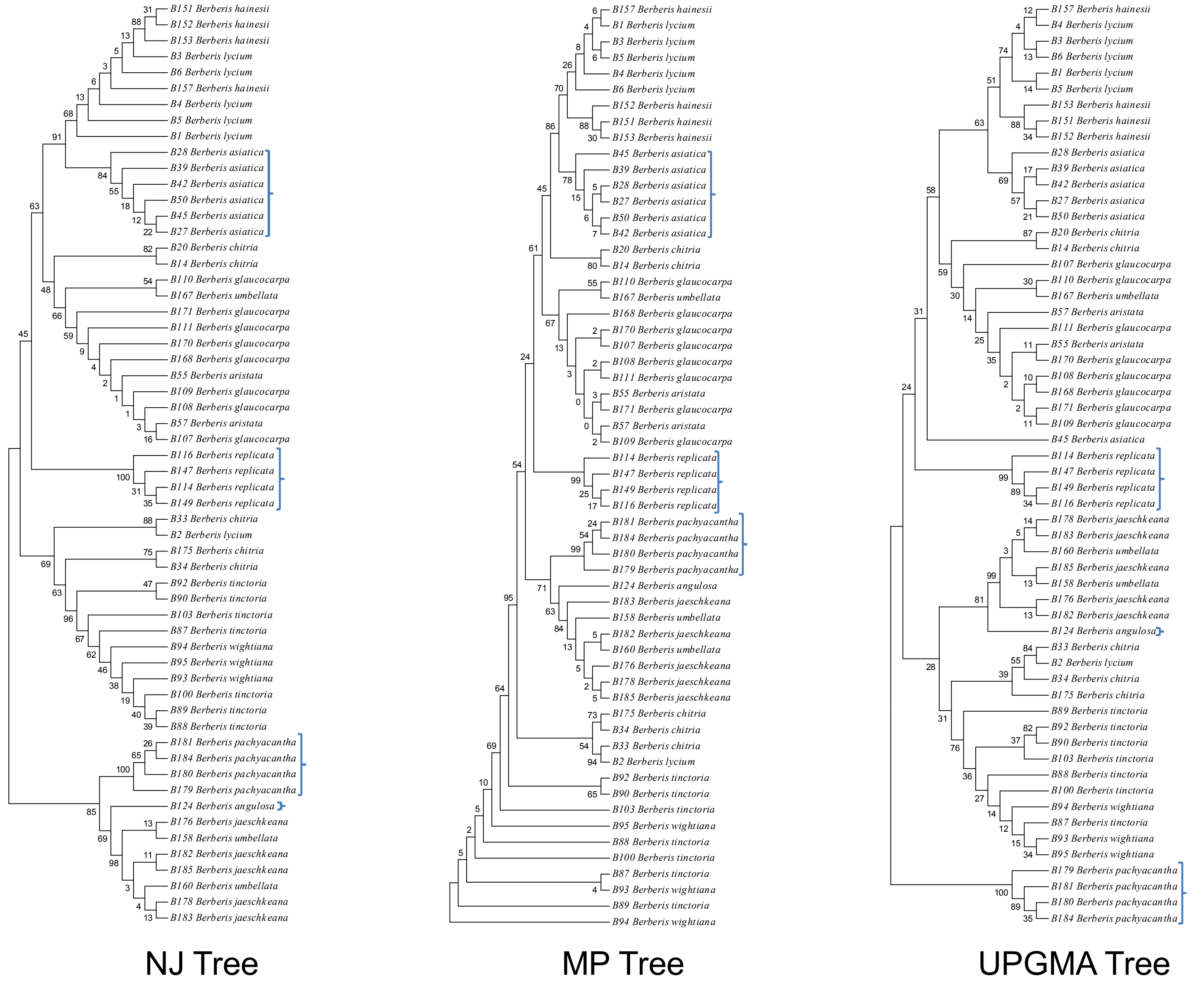

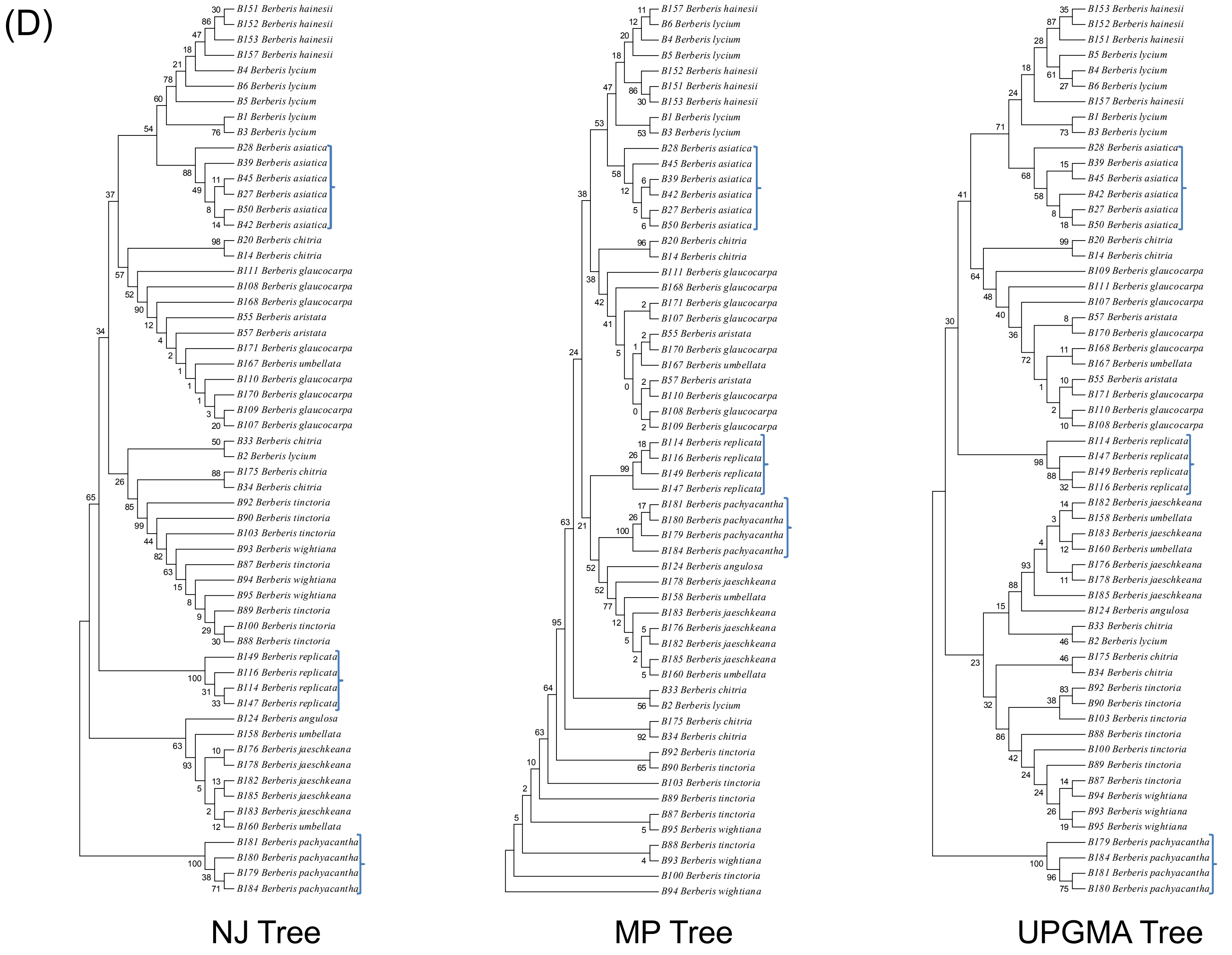
(D)
B151 Berberis hainesii
B152 Berberis hainesii
B153 Berberis hainesii
B157 Berberis hainesii
B4 Berberis lycium
B6 Berberis lycium
B5 Berberis lycium
B1 Berberis lycium
B3 Berberis lycium
B28 Berberis asiatica
B39 Berberis asiatica
B45 Berberis asiatica
B27 Berberis asiatica
B50 Berberis asiatica
B42 Berberis asiatica
B20 Berberis chitria
B14 Berberis chitria
B111 Berberis glaucocarpa
B108 Berberis glaucocarpa
B168 Berberis glaucocarpa
B55 Berberis aristata
B57 Berberis aristata
B171 Berberis glaucocarpa
B167 Berberis umbellata
B110 Berberis glaucocarpa
B170 Berberis glaucocarpa
B109 Berberis glaucocarpa
B107 Berberis glaucocarpa
B33 Berberis chitria
B2 Berberis lycium
B175 Berberis chitria
B34 Berberis chitria
B92 Berberis tinctoria
B90 Berberis tinctoria
B103 Berberis tinctoria
B93 Berberis wightiana
B87 Berberis tinctoria
B94 Berberis wightiana
B95 Berberis wightiana
B89 Berberis tinctoria
B100 Berberis tinctoria
B88 Berberis tinctoria
B149 Berberis replicata
B116 Berberis replicata
B114 Berberis replicata
B147 Berberis replicata
B124 Berberis angulosa
B158 Berberis umbellata
B176 Berberis jaeschkeana
B178 Berberis jaeschkeana
B182 Berberis jaeschkeana
B185 Berberis jaeschkeana
B183 Berberis jaeschkeana
B160 Berberis umbellata
B181 Berberis pachyacantha
B180 Berberis pachyacantha
B179 Berberis pachyacantha
B184 Berberis pachyacantha
NJ Tree
B157 Berberis hainesii
B6 Berberis lycium
B4 Berberis lycium
B5 Berberis lycium
B152 Berberis hainesii
B151 Berberis hainesii
B153 Berberis hainesii
B1 Berberis lycium
B3 Berberis lycium
B28 Berberis asiatica
B45 Berberis asiatica
B39 Berberis asiatica
B42 Berberis asiatica
B27 Berberis asiatica
B50 Berberis asiatica
B20 Berberis chitria
B14 Berberis chitria
B111 Berberis glaucocarpa
B168 Berberis glaucocarpa
B171 Berberis glaucocarpa
B107 Berberis glaucocarpa
B55 Berberis aristata
B170 Berberis glaucocarpa
B167 Berberis umbellata
B57 Berberis aristata
B110 Berberis glaucocarpa
B108 Berberis glaucocarpa
B109 Berberis glaucocarpa
B114 Berberis replicata
B116 Berberis replicata
B149 Berberis replicata
B147 Berberis replicata
B181 Berberis pachyacantha
B180 Berberis pachyacantha
B179 Berberis pachyacantha
B184 Berberis pachyacantha
B124 Berberis angulosa
B178 Berberis jaeschkeana
B158 Berberis umbellata
B183 Berberis jaeschkeana
B176 Berberis jaeschkeana
B182 Berberis jaeschkeana
B185 Berberis jaeschkeana
B160 Berberis umbellata
B33 Berberis chitria
B2 Berberis lycium
B175 Berberis chitria
B34 Berberis chitria
B92 Berberis tinctoria
B90 Berberis tinctoria
B103 Berberis tinctoria
B89 Berberis tinctoria
B87 Berberis tinctoria
B95 Berberis wightiana
B88 Berberis tinctoria
B93 Berberis wightiana
B100 Berberis tinctoria
B94 Berberis wightiana
MP Tree
B153 Berberis hainesii
B152 Berberis hainesii
B151 Berberis hainesii
B5 Berberis lycium
B4 Berberis lycium
B6 Berberis lycium
B157 Berberis hainesii
B1 Berberis lycium
B3 Berberis lycium
B28 Berberis asiatica
B39 Berberis asiatica
B45 Berberis asiatica
B42 Berberis asiatica
B27 Berberis asiatica
B50 Berberis asiatica
B20 Berberis chitria
B14 Berberis chitria
B109 Berberis glaucocarpa
B111 Berberis glaucocarpa
B107 Berberis glaucocarpa
B57 Berberis aristata
B170 Berberis glaucocarpa
B168 Berberis glaucocarpa
B167 Berberis umbellata
B55 Berberis aristata
B171 Berberis glaucocarpa
B110 Berberis glaucocarpa
B108 Berberis glaucocarpa
B114 Berberis replicata
B147 Berberis replicata
B149 Berberis replicata
B116 Berberis replicata
B182 Berberis jaeschkeana
B158 Berberis umbellata
B183 Berberis jaeschkeana
B160 Berberis umbellata
B176 Berberis jaeschkeana
B178 Berberis jaeschkeana
B185 Berberis jaeschkeana
B124 Berberis angulosa
B33 Berberis chitria
B2 Berberis lycium
B175 Berberis chitria
B34 Berberis chitria
B92 Berberis tinctoria
B90 Berberis tinctoria
B103 Berberis tinctoria
B88 Berberis tinctoria
B100 Berberis tinctoria
B89 Berberis tinctoria
B87 Berberis tinctoria
B94 Berberis wightiana
B93 Berberis wightiana
B95 Berberis wightiana
B179 Berberis pachyacantha
B184 Berberis pachyacantha
B181 Berberis pachyacantha
B180 Berberis pachyacantha
UPGMA Tree

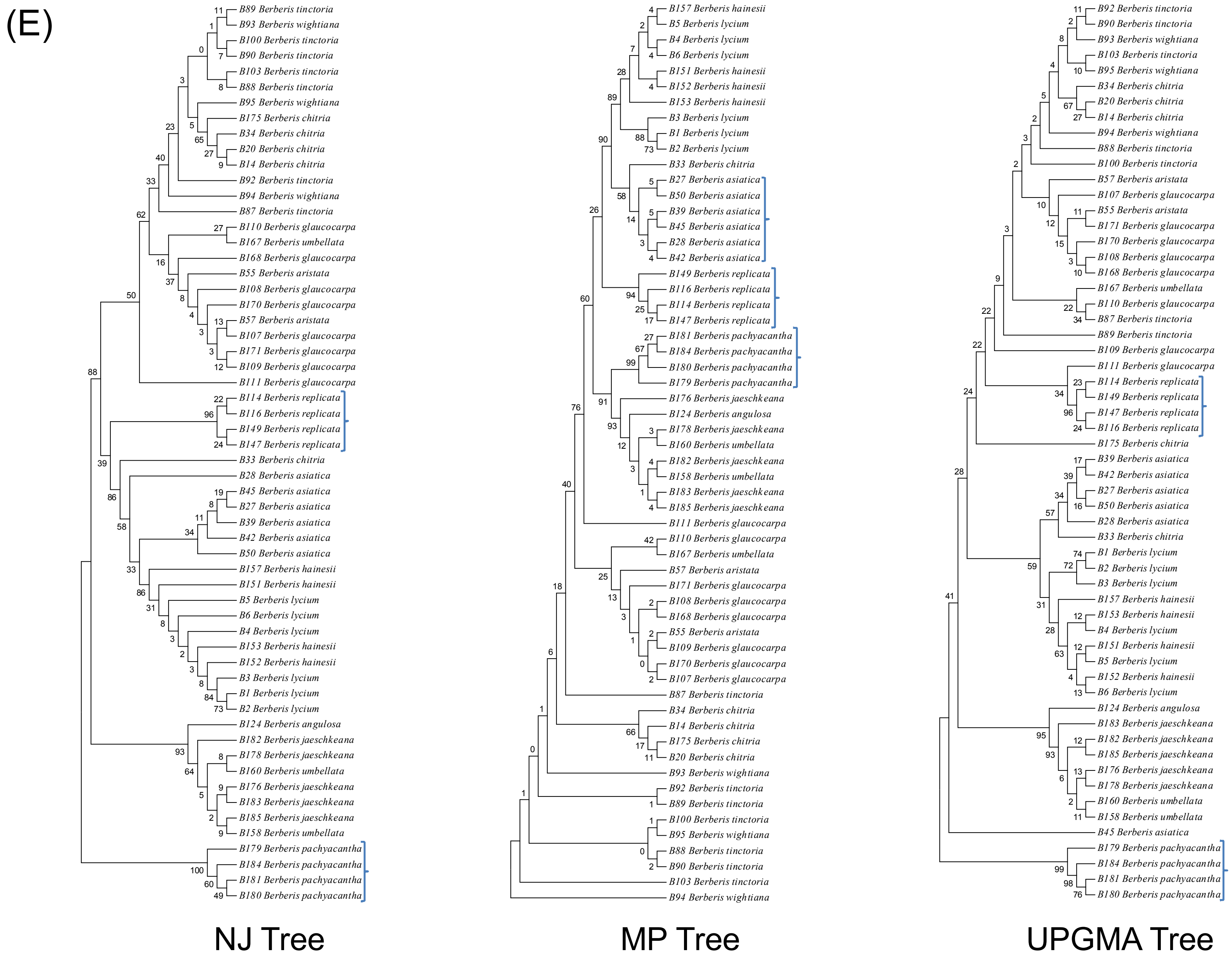
(E)
B89 Berberis tinctoria
B93 Berberis wightiana
B100 Berberis tinctoria
B90 Berberis tinctoria
B103 Berberis tinctoria
B88 Berberis tinctoria
B95 Berberis wightiana
B175 Berberis chitria
B34 Berberis chitria
B20 Berberis chitria
B14 Berberis chitria
B92 Berberis tinctoria
B94 Berberis wightiana
B87 Berberis tinctoria
B110 Berberis glaucocarpa
B167 Berberis umbellata
B168 Berberis glaucocarpa
B55 Berberis aristata
B108 Berberis glaucocarpa
B170 Berberis glaucocarpa
B57 Berberis aristata
B107 Berberis glaucocarpa
B171 Berberis glaucocarpa
B109 Berberis glaucocarpa
B111 Berberis glaucocarpa
B114 Berberis replicata
B116 Berberis replicata
B149 Berberis replicata
B147 Berberis replicata
B33 Berberis chitria
B28 Berberis asiatica
B45 Berberis asiatica
B27 Berberis asiatica
B39 Berberis asiatica
B42 Berberis asiatica
B50 Berberis asiatica
B157 Berberis hainesii
B151 Berberis hainesii
B5 Berberis lycium
B6 Berberis lycium
B4 Berberis lycium
B153 Berberis hainesii
B152 Berberis hainesii
B3 Berberis lycium
B1 Berberis lycium
B2 Berberis lycium
B124 Berberis angulosa
B182 Berberis jaeschkeana
B178 Berberis jaeschkeana
B160 Berberis umbellata
B176 Berberis jaeschkeana
B183 Berberis jaeschkeana
B185 Berberis jaeschkeana
B158 Berberis umbellata
B179 Berberis pachyacantha
B184 Berberis pachyacantha
B181 Berberis pachyacantha
B180 Berberis pachyacantha
NJ Tree
B157 Berberis hainesii
B5 Berberis lycium
B4 Berberis lycium
B6 Berberis lycium
B151 Berberis hainesii
B152 Berberis hainesii
B153 Berberis hainesii
B3 Berberis lycium
B1 Berberis lycium
B2 Berberis lycium
B33 Berberis chitria
B27 Berberis asiatica
B50 Berberis asiatica
B39 Berberis asiatica
B45 Berberis asiatica
B28 Berberis asiatica
B42 Berberis asiatica
B149 Berberis replicata
B116 Berberis replicata
B114 Berberis replicata
B147 Berberis replicata
B181 Berberis pachyacantha
B184 Berberis pachyacantha
B180 Berberis pachyacantha
B179 Berberis pachyacantha
B176 Berberis jaeschkeana
B124 Berberis angulosa
B178 Berberis jaeschkeana
B160 Berberis umbellata
B182 Berberis jaeschkeana
B158 Berberis umbellata
B183 Berberis jaeschkeana
B185 Berberis jaeschkeana
B111 Berberis glaucocarpa
B110 Berberis glaucocarpa
B167 Berberis umbellata
B57 Berberis aristata
B171 Berberis glaucocarpa
B108 Berberis glaucocarpa
B168 Berberis glaucocarpa
B55 Berberis aristata
B109 Berberis glaucocarpa
B170 Berberis glaucocarpa
B107 Berberis glaucocarpa
B87 Berberis tinctoria
B34 Berberis chitria
B14 Berberis chitria
B175 Berberis chitria
B20 Berberis chitria
B93 Berberis wightiana
B92 Berberis tinctoria
B89 Berberis tinctoria
B100 Berberis tinctoria
B95 Berberis wightiana
B88 Berberis tinctoria
B90 Berberis tinctoria
B103 Berberis tinctoria
B94 Berberis wightiana
MP Tree
B92 Berberis tinctoria
B90 Berberis tinctoria
B93 Berberis wightiana
B103 Berberis tinctoria
B95 Berberis wightiana
B34 Berberis chitria
B20 Berberis chitria
B14 Berberis chitria
B94 Berberis wightiana
B88 Berberis tinctoria
B100 Berberis tinctoria
B57 Berberis aristata
B107 Berberis glaucocarpa
B55 Berberis aristata
B171 Berberis glaucocarpa
B170 Berberis glaucocarpa
B108 Berberis glaucocarpa
B168 Berberis glaucocarpa
B167 Berberis umbellata
B110 Berberis glaucocarpa
B87 Berberis tinctoria
B89 Berberis tinctoria
B109 Berberis glaucocarpa
B111 Berberis glaucocarpa
B114 Berberis replicata
B149 Berberis replicata
B147 Berberis replicata
B116 Berberis replicata
B175 Berberis chitria
B39 Berberis asiatica
B42 Berberis asiatica
B27 Berberis asiatica
B50 Berberis asiatica
B28 Berberis asiatica
B33 Berberis chitria
B1 Berberis lycium
B2 Berberis lycium
B3 Berberis lycium
B157 Berberis hainesii
B153 Berberis hainesii
B4 Berberis lycium
B151 Berberis hainesii
B5 Berberis lycium
B152 Berberis hainesii
B6 Berberis lycium
B124 Berberis angulosa
B183 Berberis jaeschkeana
B182 Berberis jaeschkeana
B185 Berberis jaeschkeana
B176 Berberis jaeschkeana
B178 Berberis jaeschkeana
B160 Berberis umbellata
B158 Berberis umbellata
B45 Berberis asiatica
B179 Berberis pachyacantha
B184 Berberis pachyacantha
B181 Berberis pachyacantha
B180 Berberis pachyacantha
UPGMA Tree

(F)

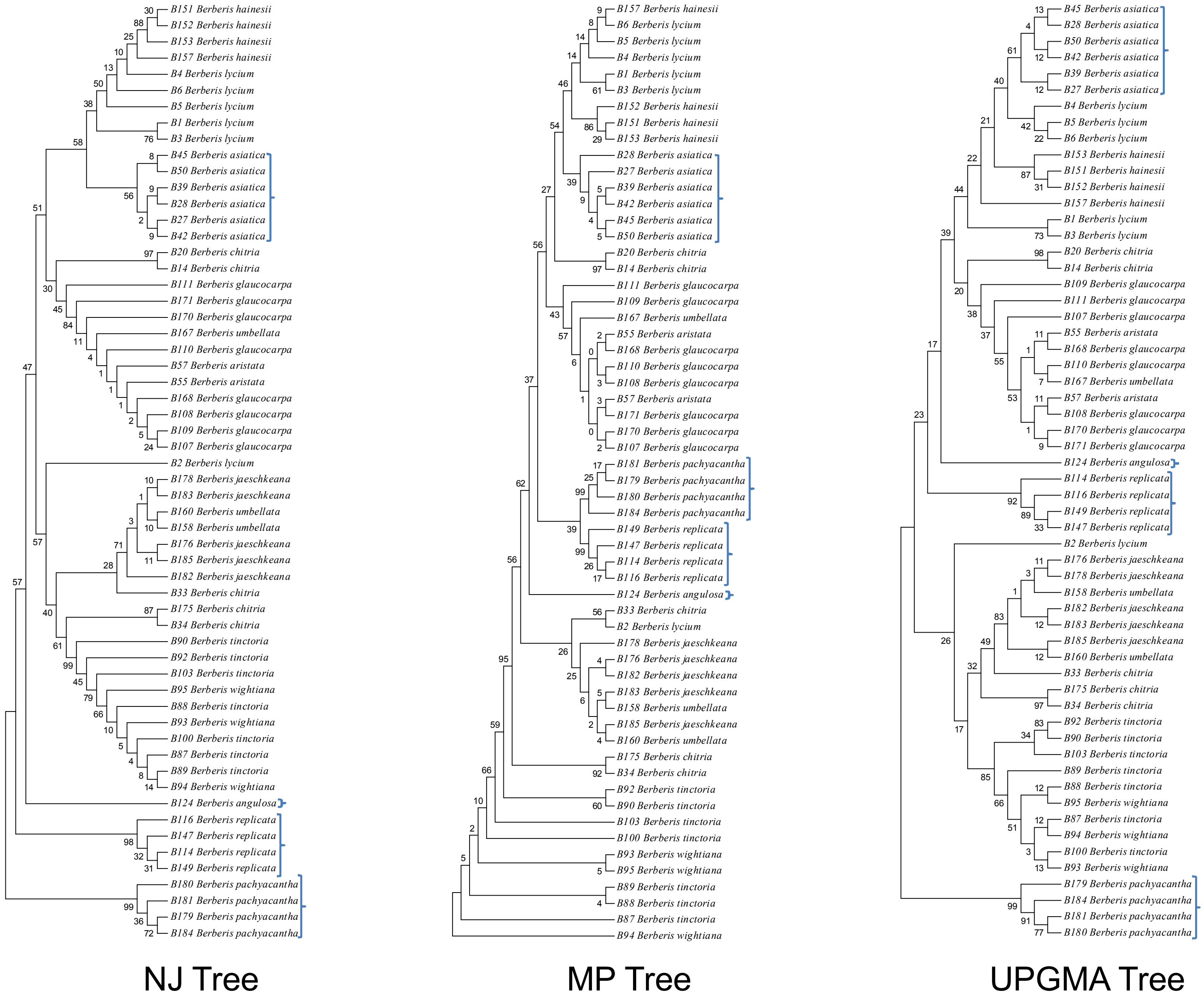

B151 Berberis hainesii
B152 Berberis hainesii
B153 Berberis hainesii
B157 Berberis hainesii
B4 Berberis lycium
B6 Berberis lycium
B5 Berberis lycium
B1 Berberis lycium
B3 Berberis lycium
B45 Berberis asiatica
B50 Berberis asiatica
B39 Berberis asiatica
B28 Berberis asiatica
B27 Berberis asiatica
B42 Berberis asiatica
B20 Berberis chitria
B14 Berberis chitria
B111 Berberis glaucocarpa
B171 Berberis glaucocarpa
B170 Berberis glaucocarpa
B167 Berberis umbellata
B110 Berberis glaucocarpa
B57 Berberis aristata
B55 Berberis aristata
B168 Berberis glaucocarpa
B108 Berberis glaucocarpa
B109 Berberis glaucocarpa
B107 Berberis glaucocarpa
B2 Berberis lycium
B178 Berberis jaeschkeana
B183 Berberis jaeschkeana
B160 Berberis umbellata
B158 Berberis umbellata
B176 Berberis jaeschkeana
B185 Berberis jaeschkeana
B182 Berberis jaeschkeana
B33 Berberis chitria
B175 Berberis chitria
B34 Berberis chitria
B90 Berberis tinctoria
B92 Berberis tinctoria
B103 Berberis tinctoria
B95 Berberis wightiana
B88 Berberis tinctoria
B93 Berberis wightiana
B100 Berberis tinctoria
B87 Berberis tinctoria
B89 Berberis tinctoria
B94 Berberis wightiana
B124 Berberis angulosa
B116 Berberis replicata
B147 Berberis replicata
B114 Berberis replicata
B149 Berberis replicata
B180 Berberis pachyacantha
B181 Berberis pachyacantha
B179 Berberis pachyacantha
B184 Berberis pachyacantha
NJ Tree
B157 Berberis hainesii
B6 Berberis lycium
B5 Berberis lycium
B4 Berberis lycium
B1 Berberis lycium
B3 Berberis lycium
B152 Berberis hainesii
B151 Berberis hainesii
B153 Berberis hainesii
B28 Berberis asiatica
B27 Berberis asiatica
B39 Berberis asiatica
B42 Berberis asiatica
B45 Berberis asiatica
B50 Berberis asiatica
B20 Berberis chitria
B14 Berberis chitria
B111 Berberis glaucocarpa
B109 Berberis glaucocarpa
B167 Berberis umbellata
B55 Berberis aristata
B168 Berberis glaucocarpa
B110 Berberis glaucocarpa
B108 Berberis glaucocarpa
B57 Berberis aristata
B171 Berberis glaucocarpa
B170 Berberis glaucocarpa
B107 Berberis glaucocarpa
B181 Berberis pachyacantha
B179 Berberis pachyacantha
B180 Berberis pachyacantha
B184 Berberis pachyacantha
B149 Berberis replicata
B147 Berberis replicata
B114 Berberis replicata
B116 Berberis replicata
B124 Berberis angulosa
B33 Berberis chitria
B2 Berberis lycium
B178 Berberis jaeschkeana
B176 Berberis jaeschkeana
B182 Berberis jaeschkeana
B183 Berberis jaeschkeana
B158 Berberis umbellata
B185 Berberis jaeschkeana
B160 Berberis umbellata
B175 Berberis chitria
B34 Berberis chitria
B92 Berberis tinctoria
B90 Berberis tinctoria
B103 Berberis tinctoria
B100 Berberis tinctoria
B93 Berberis wightiana
B95 Berberis wightiana
B89 Berberis tinctoria
B88 Berberis tinctoria
B87 Berberis tinctoria
B94 Berberis wightiana
MP Tree
B45 Berberis asiatica
B28 Berberis asiatica
B50 Berberis asiatica
B42 Berberis asiatica
B39 Berberis asiatica
B27 Berberis asiatica
B4 Berberis lycium
B5 Berberis lycium
B6 Berberis lycium
B153 Berberis hainesii
B151 Berberis hainesii
B152 Berberis hainesii
B157 Berberis hainesii
B1 Berberis lycium
B3 Berberis lycium
B20 Berberis chitria
B14 Berberis chitria
B109 Berberis glaucocarpa
B111 Berberis glaucocarpa
B107 Berberis glaucocarpa
B55 Berberis aristata
B168 Berberis glaucocarpa
B110 Berberis glaucocarpa
B167 Berberis umbellata
B57 Berberis aristata
B108 Berberis glaucocarpa
B170 Berberis glaucocarpa
B171 Berberis glaucocarpa
B124 Berberis angulosa
B114 Berberis replicata
B116 Berberis replicata
B149 Berberis replicata
B147 Berberis replicata
B2 Berberis lycium
B176 Berberis jaeschkeana
B178 Berberis jaeschkeana
B158 Berberis umbellata
B182 Berberis jaeschkeana
B183 Berberis jaeschkeana
B185 Berberis jaeschkeana
B160 Berberis umbellata
B33 Berberis chitria
B175 Berberis chitria
B34 Berberis chitria
B92 Berberis tinctoria
B90 Berberis tinctoria
B103 Berberis tinctoria
B89 Berberis tinctoria
B88 Berberis tinctoria
B95 Berberis wightiana
B87 Berberis tinctoria
B94 Berberis wightiana
B100 Berberis tinctoria
B93 Berberis wightiana
B179 Berberis pachyacantha
B184 Berberis pachyacantha
B181 Berberis pachyacantha
B180 Berberis pachyacantha
UPGMA Tree

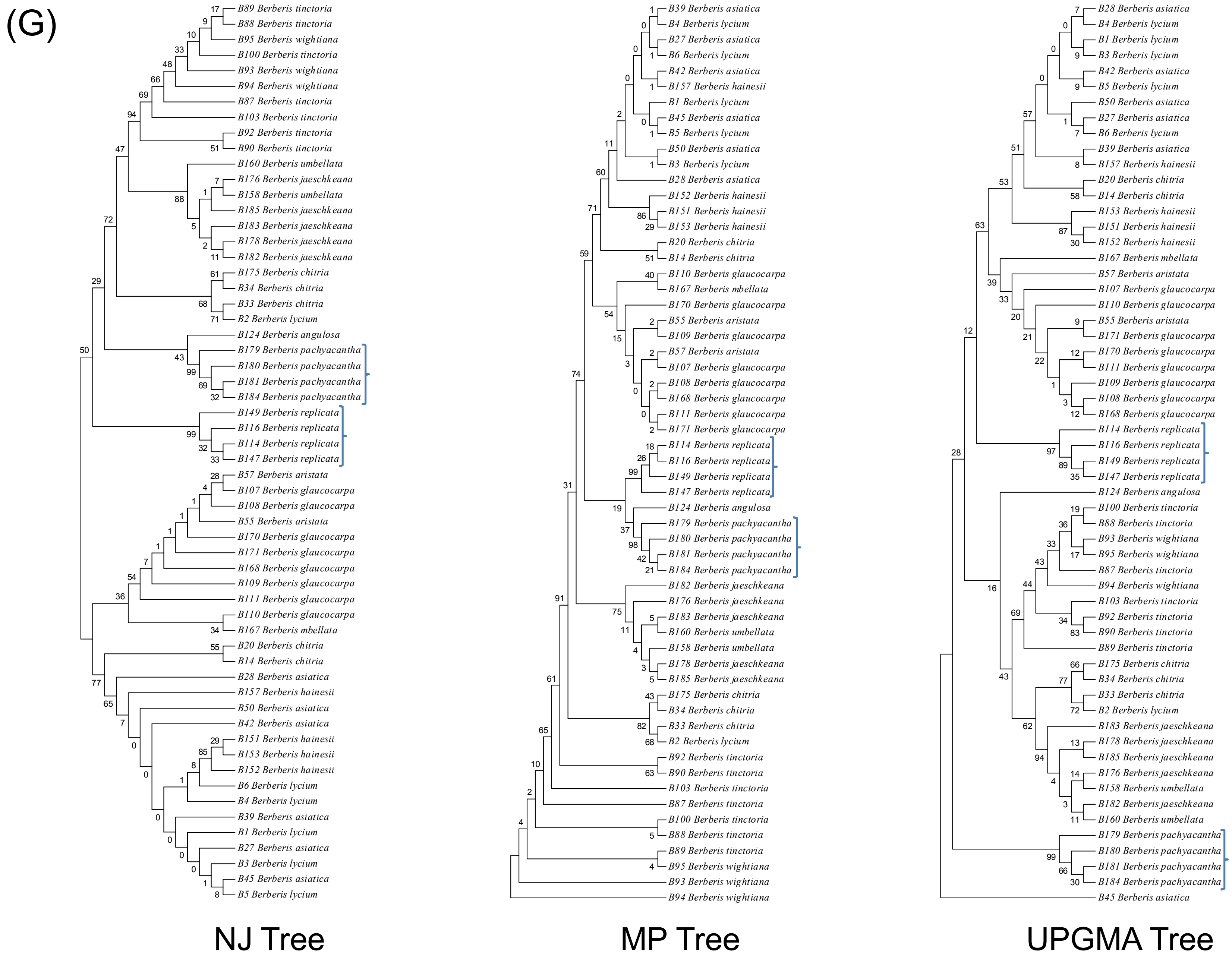

(G)
B89 Berberis tinctoria
B88 Berberis tinctoria
B95 Berberis wightiana
B100 Berberis tinctoria
B93 Berberis wightiana
B94 Berberis wightiana
B87 Berberis tinctoria
B103 Berberis tinctoria
B92 Berberis tinctoria
B90 Berberis tinctoria
B160 Berberis umbellata
B176 Berberis jaeschkeana
B158 Berberis umbellata
B185 Berberis jaeschkeana
B183 Berberis jaeschkeana
B178 Berberis jaeschkeana
B182 Berberis jaeschkeana
B175 Berberis chitria
B34 Berberis chitria
B33 Berberis chitria
B2 Berberis lycium
B124 Berberis angulosa
B179 Berberis pachyacantha
B180 Berberis pachyacantha
B181 Berberis pachyacantha
B184 Berberis pachyacantha
B149 Berberis replicata
B116 Berberis replicata
B114 Berberis replicata
B147 Berberis replicata
B57 Berberis aristata
B107 Berberis glaucocarpa
B108 Berberis glaucocarpa
B55 Berberis aristata
B170 Berberis glaucocarpa
B171 Berberis glaucocarpa
B168 Berberis glaucocarpa
B109 Berberis glaucocarpa
B111 Berberis glaucocarpa
B110 Berberis glaucocarpa
B167 Berberis mbellata
B20 Berberis chitria
B14 Berberis chitria
B28 Berberis asiatica
B157 Berberis hainesii
B50 Berberis asiatica
B42 Berberis asiatica
B151 Berberis hainesii
B153 Berberis hainesii
B152 Berberis hainesii
B6 Berberis lycium
B4 Berberis lycium
B39 Berberis asiatica
B1 Berberis lycium
B27 Berberis asiatica
B3 Berberis lycium
B45 Berberis asiatica
B5 Berberis lycium
NJ Tree
B39 Berberis asiatica
B4 Berberis lycium
B27 Berberis asiatica
B6 Berberis lycium
B42 Berberis asiatica
B157 Berberis hainesii
B1 Berberis lycium
B45 Berberis asiatica
B5 Berberis lycium
B50 Berberis asiatica
B3 Berberis lycium
B28 Berberis asiatica
B152 Berberis hainesii
B151 Berberis hainesii
B153 Berberis hainesii
B20 Berberis chitria
B14 Berberis chitria
B110 Berberis glaucocarpa
B167 Berberis mbellata
B170 Berberis glaucocarpa
B55 Berberis aristata
B109 Berberis glaucocarpa
B57 Berberis aristata
B107 Berberis glaucocarpa
B108 Berberis glaucocarpa
B168 Berberis glaucocarpa
B111 Berberis glaucocarpa
B171 Berberis glaucocarpa
B114 Berberis replicata
B116 Berberis replicata
B149 Berberis replicata
B147 Berberis replicata
B124 Berberis angulosa
B179 Berberis pachyacantha
B180 Berberis pachyacantha
B181 Berberis pachyacantha
B184 Berberis pachyacantha
B182 Berberis jaeschkeana
B176 Berberis jaeschkeana
B183 Berberis jaeschkeana
B160 Berberis umbellata
B158 Berberis umbellata
B178 Berberis jaeschkeana
B185 Berberis jaeschkeana
B175 Berberis chitria
B34 Berberis chitria
B33 Berberis chitria
B2 Berberis lycium
B92 Berberis tinctoria
B90 Berberis tinctoria
B103 Berberis tinctoria
B87 Berberis tinctoria
B100 Berberis tinctoria
B88 Berberis tinctoria
B89 Berberis tinctoria
B95 Berberis wightiana
B93 Berberis wightiana
B94 Berberis wightiana
MP Tree
B28 Berberis asiatica
B4 Berberis lycium
B1 Berberis lycium
B3 Berberis lycium
B42 Berberis asiatica
B5 Berberis lycium
B50 Berberis asiatica
B27 Berberis asiatica
B6 Berberis lycium
B39 Berberis asiatica
B157 Berberis hainesii
B20 Berberis chitria
B14 Berberis chitria
B153 Berberis hainesii
B151 Berberis hainesii
B152 Berberis hainesii
B167 Berberis mbellata
B57 Berberis aristata
B107 Berberis glaucocarpa
B110 Berberis glaucocarpa
B55 Berberis aristata
B171 Berberis glaucocarpa
B170 Berberis glaucocarpa
B111 Berberis glaucocarpa
B109 Berberis glaucocarpa
B108 Berberis glaucocarpa
B168 Berberis glaucocarpa
B114 Berberis replicata
B116 Berberis replicata
B149 Berberis replicata
B147 Berberis replicata
B124 Berberis angulosa
B100 Berberis tinctoria
B88 Berberis tinctoria
B93 Berberis wightiana
B95 Berberis wightiana
B87 Berberis tinctoria
B94 Berberis wightiana
B103 Berberis tinctoria
B92 Berberis tinctoria
B90 Berberis tinctoria
B89 Berberis tinctoria
B175 Berberis chitria
B34 Berberis chitria
B33 Berberis chitria
B2 Berberis lycium
B183 Berberis jaeschkeana
B178 Berberis jaeschkeana
B185 Berberis jaeschkeana
B176 Berberis jaeschkeana
B158 Berberis umbellata
B182 Berberis jaeschkeana
B160 Berberis umbellata
B179 Berberis pachyacantha
B180 Berberis pachyacantha
B181 Berberis pachyacantha
B184 Berberis pachyacantha
B45 Berberis asiatica
UPGMA Tree

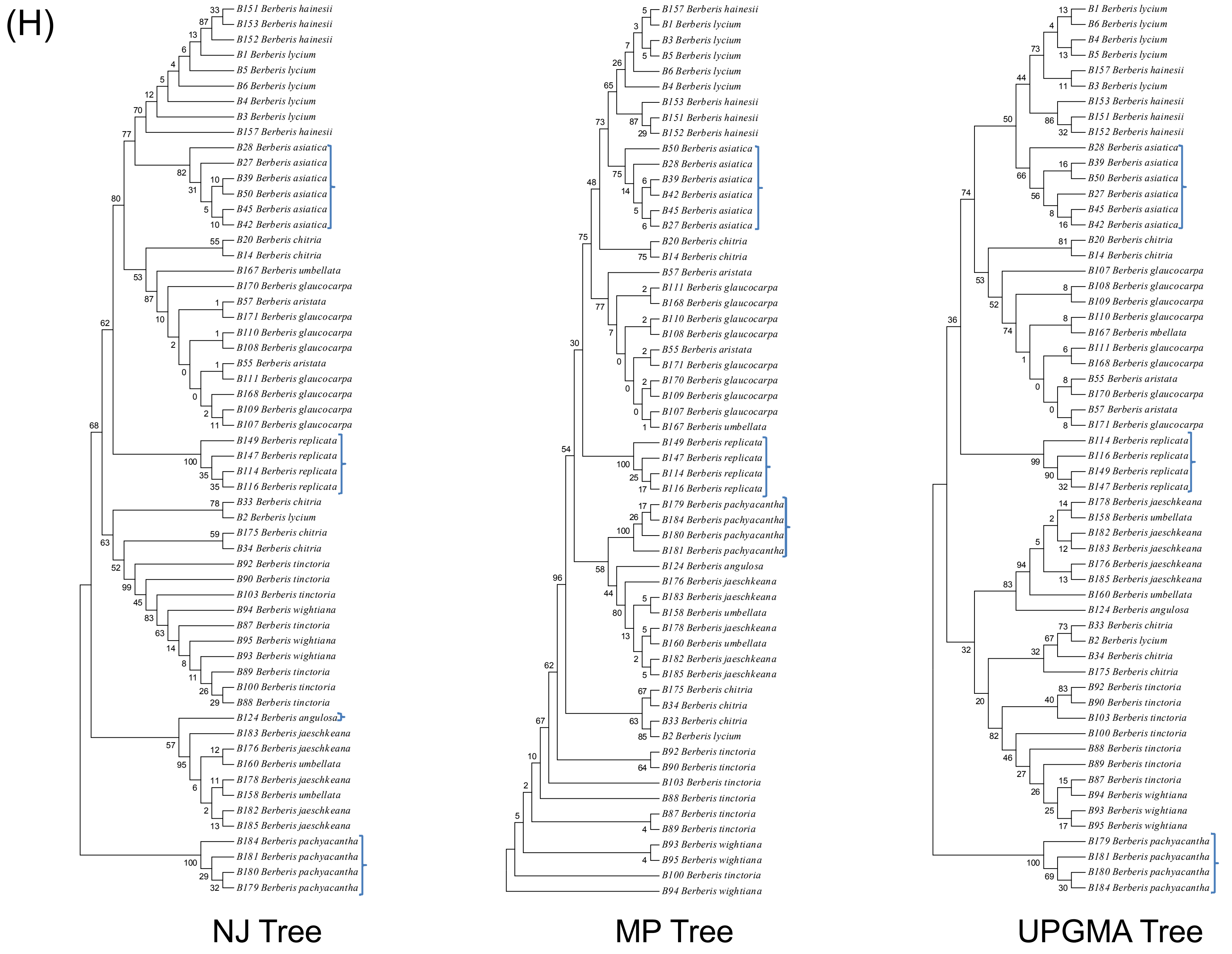
(H)
B151 Berberis hainesii
B153 Berberis hainesii
B152 Berberis hainesii
B1 Berberis lycium
B5 Berberis lycium
B6 Berberis lycium
B4 Berberis lycium
B3 Berberis lycium
B157 Berberis hainesii
B28 Berberis asiatica
B27 Berberis asiatica
B39 Berberis asiatica
B50 Berberis asiatica
B45 Berberis asiatica
B42 Berberis asiatica
B20 Berberis chitria
B14 Berberis chitria
B167 Berberis umbellata
B170 Berberis glaucocarpa
B57 Berberis aristata
B171 Berberis glaucocarpa
B110 Berberis glaucocarpa
B108 Berberis glaucocarpa
B55 Berberis aristata
B111 Berberis glaucocarpa
B168 Berberis glaucocarpa
B109 Berberis glaucocarpa
B107 Berberis glaucocarpa
B149 Berberis replicata
B147 Berberis replicata
B114 Berberis replicata
B116 Berberis replicata
B33 Berberis chitria
B2 Berberis lycium
B175 Berberis chitria
B34 Berberis chitria
B92 Berberis tinctoria
B90 Berberis tinctoria
B103 Berberis tinctoria
B94 Berberis wightiana
B87 Berberis tinctoria
B95 Berberis wightiana
B93 Berberis wightiana
B89 Berberis tinctoria
B100 Berberis tinctoria
B88 Berberis tinctoria
B124 Berberis angulosa
B183 Berberis jaeschkeana
B176 Berberis jaeschkeana
B160 Berberis umbellata
B178 Berberis jaeschkeana
B158 Berberis umbellata
B182 Berberis jaeschkeana
B185 Berberis jaeschkeana
B184 Berberis pachyacantha
B181 Berberis pachyacantha
B180 Berberis pachyacantha
B179 Berberis pachyacantha
NJ Tree
B157 Berberis hainesii
B1 Berberis lycium
B3 Berberis lycium
B5 Berberis lycium
B6 Berberis lycium
B4 Berberis lycium
B153 Berberis hainesii
B151 Berberis hainesii
B152 Berberis hainesii
B50 Berberis asiatica
B28 Berberis asiatica
B39 Berberis asiatica
B42 Berberis asiatica
B45 Berberis asiatica
B27 Berberis asiatica
B20 Berberis chitria
B14 Berberis chitria
B57 Berberis aristata
B111 Berberis glaucocarpa
B168 Berberis glaucocarpa
B110 Berberis glaucocarpa
B108 Berberis glaucocarpa
B55 Berberis aristata
B171 Berberis glaucocarpa
B170 Berberis glaucocarpa
B109 Berberis glaucocarpa
B107 Berberis glaucocarpa
B167 Berberis umbellata
B149 Berberis replicata
B147 Berberis replicata
B114 Berberis replicata
B116 Berberis replicata
B179 Berberis pachyacantha
B184 Berberis pachyacantha
B180 Berberis pachyacantha
B181 Berberis pachyacantha
B124 Berberis angulosa
B176 Berberis jaeschkeana
B183 Berberis jaeschkeana
B158 Berberis umbellata
B178 Berberis jaeschkeana
B160 Berberis umbellata
B182 Berberis jaeschkeana
B185 Berberis jaeschkeana
B175 Berberis chitria
B34 Berberis chitria
B33 Berberis chitria
B2 Berberis lycium
B92 Berberis tinctoria
B90 Berberis tinctoria
B103 Berberis tinctoria
B88 Berberis tinctoria
B87 Berberis tinctoria
B89 Berberis tinctoria
B93 Berberis wightiana
B95 Berberis wightiana
B100 Berberis tinctoria
B94 Berberis wightiana
MP Tree
B1 Berberis lycium
B6 Berberis lycium
B4 Berberis lycium
B5 Berberis lycium
B157 Berberis hainesii
B3 Berberis lycium
B153 Berberis hainesii
B151 Berberis hainesii
B152 Berberis hainesii
B28 Berberis asiatica
B39 Berberis asiatica
B50 Berberis asiatica
B27 Berberis asiatica
B45 Berberis asiatica
B42 Berberis asiatica
B20 Berberis chitria
B14 Berberis chitria
B107 Berberis glaucocarpa
B108 Berberis glaucocarpa
B109 Berberis glaucocarpa
B110 Berberis glaucocarpa
B167 Berberis mbellata
B111 Berberis glaucocarpa
B168 Berberis glaucocarpa
B55 Berberis aristata
B170 Berberis glaucocarpa
B57 Berberis aristata
B171 Berberis glaucocarpa
B114 Berberis replicata
B116 Berberis replicata
B149 Berberis replicata
B147 Berberis replicata
B178 Berberis jaeschkeana
B158 Berberis umbellata
B182 Berberis jaeschkeana
B183 Berberis jaeschkeana
B176 Berberis jaeschkeana
B185 Berberis jaeschkeana
B160 Berberis umbellata
B124 Berberis angulosa
B33 Berberis chitria
B2 Berberis lycium
B34 Berberis chitria
B175 Berberis chitria
B92 Berberis tinctoria
B90 Berberis tinctoria
B103 Berberis tinctoria
B100 Berberis tinctoria
B88 Berberis tinctoria
B89 Berberis tinctoria
B87 Berberis tinctoria
B94 Berberis wightiana
B93 Berberis wightiana
B95 Berberis wightiana
B179 Berberis pachyacantha
B181 Berberis pachyacantha
B180 Berberis pachyacantha
B184 Berberis pachyacantha
UPGMA Tree

(I)

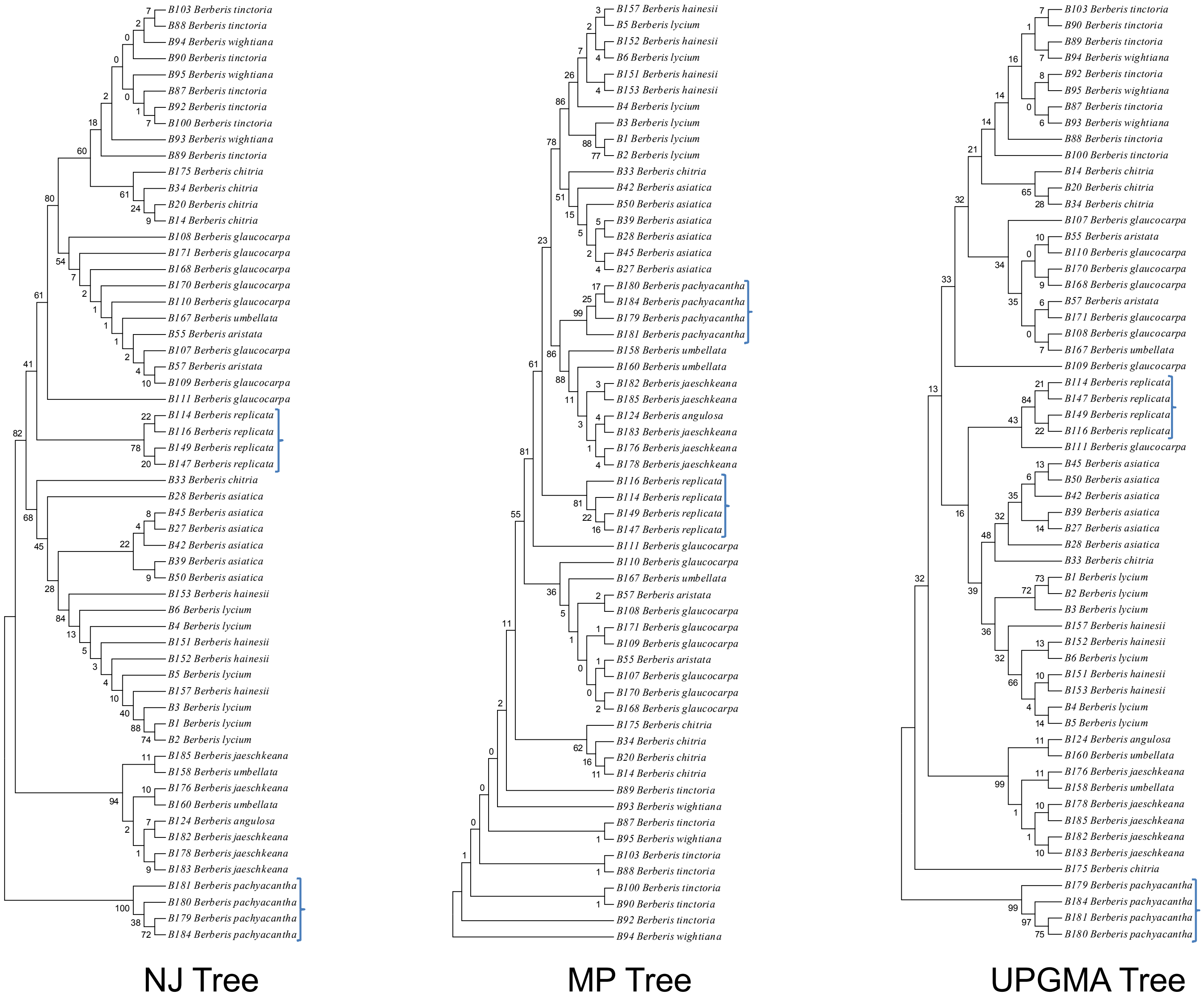
B103 Berberis tinctoria
B88 Berberis tinctoria
B94 Berberis wightiana
B90 Berberis tinctoria
B95 Berberis wightiana
B87 Berberis tinctoria
B92 Berberis tinctoria
B100 Berberis tinctoria
B93 Berberis wightiana
B89 Berberis tinctoria
B175 Berberis chitria
B34 Berberis chitria
B20 Berberis chitria
B14 Berberis chitria
B108 Berberis glaucocarpa
B171 Berberis glaucocarpa
B168 Berberis glaucocarpa
B170 Berberis glaucocarpa
B110 Berberis glaucocarpa
B167 Berberis umbellata
B55 Berberis aristata
B107 Berberis glaucocarpa
B57 Berberis aristata
B109 Berberis glaucocarpa
B111 Berberis glaucocarpa
B114 Berberis replicata
B116 Berberis replicata
B149 Berberis replicata
B147 Berberis replicata
B33 Berberis chitria
B28 Berberis asiatica
B45 Berberis asiatica
B27 Berberis asiatica
B42 Berberis asiatica
B39 Berberis asiatica
B50 Berberis asiatica
B153 Berberis hainesii
B6 Berberis lycium
B4 Berberis lycium
B151 Berberis hainesii
B152 Berberis hainesii
B5 Berberis lycium
B157 Berberis hainesii
B3 Berberis lycium
B1 Berberis lycium
B2 Berberis lycium
B185 Berberis jaeschkeana
B158 Berberis umbellata
B176 Berberis jaeschkeana
B160 Berberis umbellata
B124 Berberis angulosa
B182 Berberis jaeschkeana
B178 Berberis jaeschkeana
B183 Berberis jaeschkeana
B181 Berberis pachyacantha
B180 Berberis pachyacantha
B179 Berberis pachyacantha
B184 Berberis pachyacantha
NJ Tree
B157 Berberis hainesii
B5 Berberis lycium
B152 Berberis hainesii
B6 Berberis lycium
B151 Berberis hainesii
B153 Berberis hainesii
B4 Berberis lycium
B3 Berberis lycium
B1 Berberis lycium
B2 Berberis lycium
B33 Berberis chitria
B42 Berberis asiatica
B50 Berberis asiatica
B39 Berberis asiatica
B28 Berberis asiatica
B45 Berberis asiatica
B27 Berberis asiatica
B180 Berberis pachyacantha
B184 Berberis pachyacantha
B179 Berberis pachyacantha
B181 Berberis pachyacantha
B158 Berberis umbellata
B160 Berberis umbellata
B182 Berberis jaeschkeana
B185 Berberis jaeschkeana
B124 Berberis angulosa
B183 Berberis jaeschkeana
B176 Berberis jaeschkeana
B178 Berberis jaeschkeana
B116 Berberis replicata
B114 Berberis replicata
B149 Berberis replicata
B147 Berberis replicata
B111 Berberis glaucocarpa
B110 Berberis glaucocarpa
B167 Berberis umbellata
B57 Berberis aristata
B108 Berberis glaucocarpa
B171 Berberis glaucocarpa
B109 Berberis glaucocarpa
B55 Berberis aristata
B107 Berberis glaucocarpa
B170 Berberis glaucocarpa
B168 Berberis glaucocarpa
B175 Berberis chitria
B34 Berberis chitria
B20 Berberis chitria
B14 Berberis chitria
B89 Berberis tinctoria
B93 Berberis wightiana
B87 Berberis tinctoria
B95 Berberis wightiana
B103 Berberis tinctoria
B88 Berberis tinctoria
B100 Berberis tinctoria
B90 Berberis tinctoria
B92 Berberis tinctoria
B94 Berberis wightiana
MP Tree
B103 Berberis tinctoria
B90 Berberis tinctoria
B89 Berberis tinctoria
B94 Berberis wightiana
B92 Berberis tinctoria
B95 Berberis wightiana
B87 Berberis tinctoria
B93 Berberis wightiana
B88 Berberis tinctoria
B100 Berberis tinctoria
B14 Berberis chitria
B20 Berberis chitria
B34 Berberis chitria
B107 Berberis glaucocarpa
B55 Berberis aristata
B110 Berberis glaucocarpa
B170 Berberis glaucocarpa
B168 Berberis glaucocarpa
B57 Berberis aristata
B171 Berberis glaucocarpa
B108 Berberis glaucocarpa
B167 Berberis umbellata
B109 Berberis glaucocarpa
B114 Berberis replicata
B147 Berberis replicata
B149 Berberis replicata
B116 Berberis replicata
B111 Berberis glaucocarpa
B45 Berberis asiatica
B50 Berberis asiatica
B42 Berberis asiatica
B39 Berberis asiatica
B27 Berberis asiatica
B28 Berberis asiatica
B33 Berberis chitria
B1 Berberis lycium
B2 Berberis lycium
B3 Berberis lycium
B157 Berberis hainesii
B152 Berberis hainesii
B6 Berberis lycium
B151 Berberis hainesii
B153 Berberis hainesii
B4 Berberis lycium
B5 Berberis lycium
B124 Berberis angulosa
B160 Berberis umbellata
B176 Berberis jaeschkeana
B158 Berberis umbellata
B178 Berberis jaeschkeana
B185 Berberis jaeschkeana
B182 Berberis jaeschkeana
B183 Berberis jaeschkeana
B175 Berberis chitria
B179 Berberis pachyacantha
B184 Berberis pachyacantha
B181 Berberis pachyacantha
B180 Berberis pachyacantha
UPGMA Tree

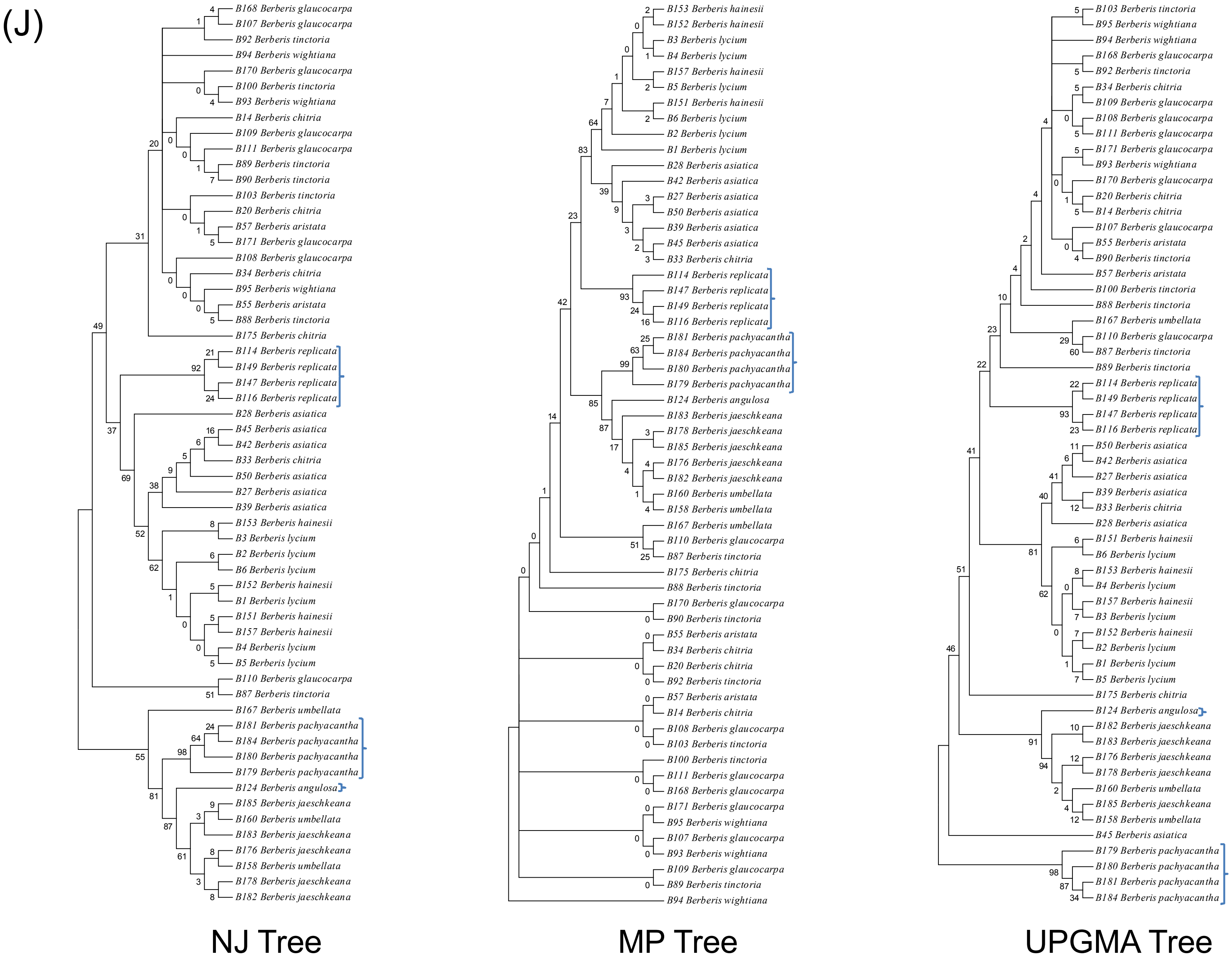
(J)
B168 Berberis glaucocarpa
B107 Berberis glaucocarpa
B92 Berberis tinctoria
B94 Berberis wightiana
B170 Berberis glaucocarpa
B100 Berberis tinctoria
B93 Berberis wightiana
B14 Berberis chitria
B109 Berberis glaucocarpa
B111 Berberis glaucocarpa
B89 Berberis tinctoria
B90 Berberis tinctoria
B103 Berberis tinctoria
B20 Berberis chitria
B57 Berberis aristata
B171 Berberis glaucocarpa
B108 Berberis glaucocarpa
B34 Berberis chitria
B95 Berberis wightiana
B55 Berberis aristata
B88 Berberis tinctoria
B175 Berberis chitria
B114 Berberis replicata
B149 Berberis replicata
B147 Berberis replicata
B116 Berberis replicata
B28 Berberis asiatica
B45 Berberis asiatica
B42 Berberis asiatica
B33 Berberis chitria
B50 Berberis asiatica
B27 Berberis asiatica
B39 Berberis asiatica
B153 Berberis hainesii
B3 Berberis lycium
B2 Berberis lycium
B6 Berberis lycium
B152 Berberis hainesii
B1 Berberis lycium
B151 Berberis hainesii
B157 Berberis hainesii
B4 Berberis lycium
B5 Berberis lycium
B110 Berberis glaucocarpa
B87 Berberis tinctoria
B167 Berberis umbellata
B181 Berberis pachyacantha
B184 Berberis pachyacantha
B180 Berberis pachyacantha
B179 Berberis pachyacantha
B124 Berberis angulosa
B185 Berberis jaeschkeana
B160 Berberis umbellata
B183 Berberis jaeschkeana
B176 Berberis jaeschkeana
B158 Berberis umbellata
B178 Berberis jaeschkeana
B182 Berberis jaeschkeana
NJ Tree
B153 Berberis hainesii
B152 Berberis hainesii
B3 Berberis lycium
B4 Berberis lycium
B157 Berberis hainesii
B5 Berberis lycium
B151 Berberis hainesii
B6 Berberis lycium
B2 Berberis lycium
B1 Berberis lycium
B28 Berberis asiatica
B42 Berberis asiatica
B27 Berberis asiatica
B50 Berberis asiatica
B39 Berberis asiatica
B45 Berberis asiatica
B33 Berberis chitria
B114 Berberis replicata
B147 Berberis replicata
B149 Berberis replicata
B116 Berberis replicata
B181 Berberis pachyacantha
B184 Berberis pachyacantha
B180 Berberis pachyacantha
B179 Berberis pachyacantha
B124 Berberis angulosa
B183 Berberis jaeschkeana
B178 Berberis jaeschkeana
B185 Berberis jaeschkeana
B176 Berberis jaeschkeana
B182 Berberis jaeschkeana
B160 Berberis umbellata
B158 Berberis umbellata
B167 Berberis umbellata
B110 Berberis glaucocarpa
B87 Berberis tinctoria
B175 Berberis chitria
B88 Berberis tinctoria
B170 Berberis glaucocarpa
B90 Berberis tinctoria
B55 Berberis aristata
B34 Berberis chitria
B20 Berberis chitria
B92 Berberis tinctoria
B57 Berberis aristata
B14 Berberis chitria
B108 Berberis glaucocarpa
B103 Berberis tinctoria
B100 Berberis tinctoria
B111 Berberis glaucocarpa
B168 Berberis glaucocarpa
B171 Berberis glaucocarpa
B95 Berberis wightiana
B107 Berberis glaucocarpa
B93 Berberis wightiana
B109 Berberis glaucocarpa
B89 Berberis tinctoria
B94 Berberis wightiana
MP Tree
B103 Berberis tinctoria
B95 Berberis wightiana
B94 Berberis wightiana
B168 Berberis glaucocarpa
B92 Berberis tinctoria
B34 Berberis chitria
B109 Berberis glaucocarpa
B108 Berberis glaucocarpa
B111 Berberis glaucocarpa
B171 Berberis glaucocarpa
B93 Berberis wightiana
B170 Berberis glaucocarpa
B20 Berberis chitria
B14 Berberis chitria
B107 Berberis glaucocarpa
B55 Berberis aristata
B90 Berberis tinctoria
B57 Berberis aristata
B100 Berberis tinctoria
B88 Berberis tinctoria
B167 Berberis umbellata
B110 Berberis glaucocarpa
B87 Berberis tinctoria
B89 Berberis tinctoria
B114 Berberis replicata
B149 Berberis replicata
B147 Berberis replicata
B116 Berberis replicata
B50 Berberis asiatica
B42 Berberis asiatica
B27 Berberis asiatica
B39 Berberis asiatica
B33 Berberis chitria
B28 Berberis asiatica
B151 Berberis hainesii
B6 Berberis lycium
B153 Berberis hainesii
B4 Berberis lycium
B157 Berberis hainesii
B3 Berberis lycium
B152 Berberis hainesii
B2 Berberis lycium
B1 Berberis lycium
B5 Berberis lycium
B175 Berberis chitria
B124 Berberis angulosa
B182 Berberis jaeschkeana
B183 Berberis jaeschkeana
B176 Berberis jaeschkeana
B178 Berberis jaeschkeana
B160 Berberis umbellata
B185 Berberis jaeschkeana
B158 Berberis umbellata
B45 Berberis asiatica
B179 Berberis pachyacantha
B180 Berberis pachyacantha
B181 Berberis pachyacantha
B184 Berberis pachyacantha
UPGMA Tree

(K)

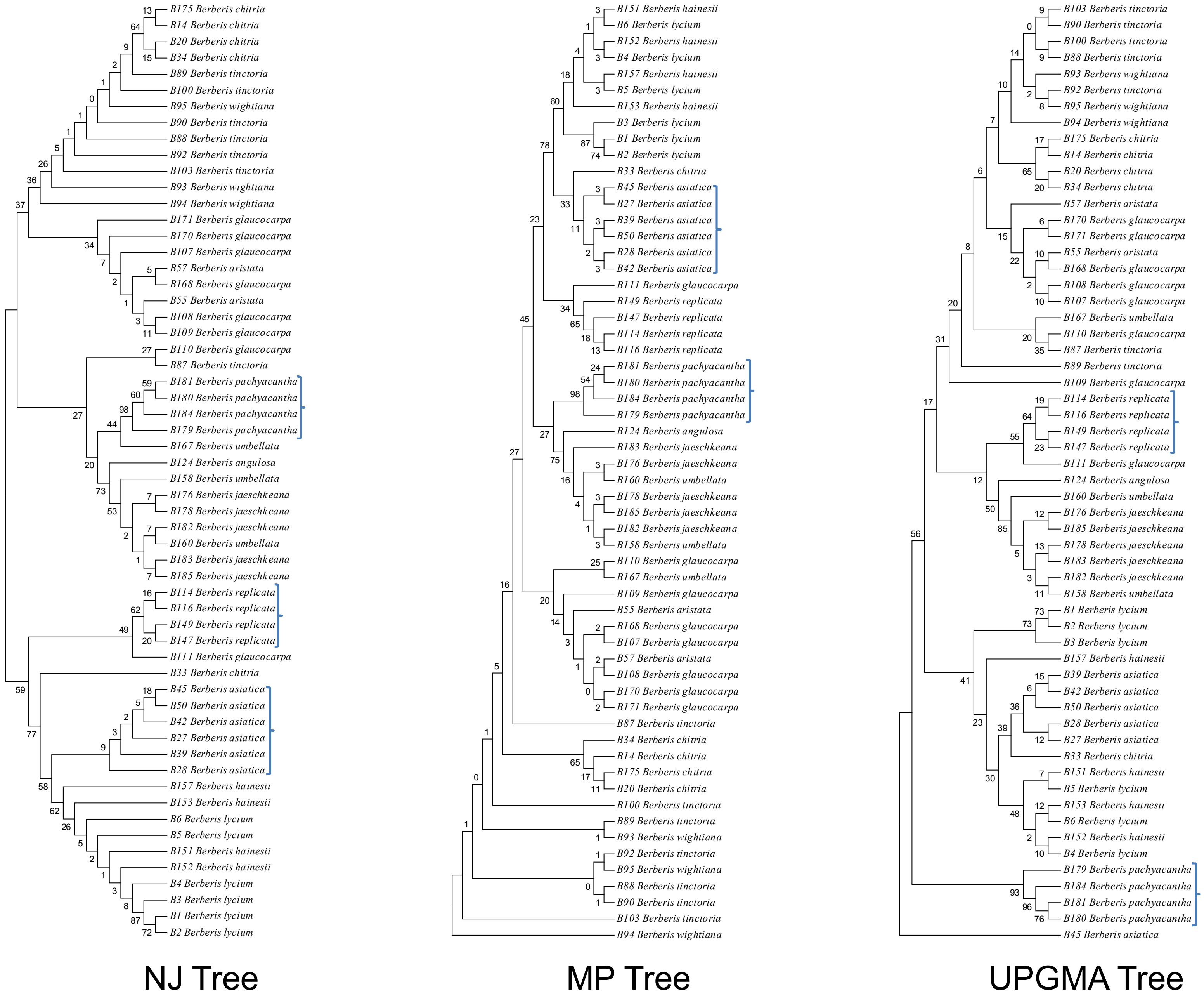
B175 Berberis chitria
B14 Berberis chitria
B20 Berberis chitria
B34 Berberis chitria
B89 Berberis tinctoria
B100 Berberis tinctoria
B95 Berberis wightiana
B90 Berberis tinctoria
B88 Berberis tinctoria
B92 Berberis tinctoria
B103 Berberis tinctoria
B93 Berberis wightiana
B94 Berberis wightiana
B171 Berberis glaucocarpa
B170 Berberis glaucocarpa
B107 Berberis glaucocarpa
B57 Berberis aristata
B168 Berberis glaucocarpa
B55 Berberis aristata
B108 Berberis glaucocarpa
B109 Berberis glaucocarpa
B110 Berberis glaucocarpa
B87 Berberis tinctoria
B181 Berberis pachyacantha
B180 Berberis pachyacantha
B184 Berberis pachyacantha
B179 Berberis pachyacantha
B167 Berberis umbellata
B124 Berberis angulosa
B158 Berberis umbellata
B176 Berberis jaeschkeana
B178 Berberis jaeschkeana
B182 Berberis jaeschkeana
B160 Berberis umbellata
B183 Berberis jaeschkeana
B185 Berberis jaeschkeana
B114 Berberis replicata
B116 Berberis replicata
B149 Berberis replicata
B147 Berberis replicata
B111 Berberis glaucocarpa
B33 Berberis chitria
B45 Berberis asiatica
B50 Berberis asiatica
B42 Berberis asiatica
B27 Berberis asiatica
B39 Berberis asiatica
B28 Berberis asiatica
B157 Berberis hainesii
B153 Berberis hainesii
B6 Berberis lycium
B5 Berberis lycium
B151 Berberis hainesii
B152 Berberis hainesii
B4 Berberis lycium
B3 Berberis lycium
B1 Berberis lycium
B2 Berberis lycium
NJ Tree
B151 Berberis hainesii
B6 Berberis lycium
B152 Berberis hainesii
B4 Berberis lycium
B157 Berberis hainesii
B5 Berberis lycium
B153 Berberis hainesii
B3 Berberis lycium
B1 Berberis lycium
B2 Berberis lycium
B33 Berberis chitria
B45 Berberis asiatica
B27 Berberis asiatica
B39 Berberis asiatica
B50 Berberis asiatica
B28 Berberis asiatica
B42 Berberis asiatica
B111 Berberis glaucocarpa
B149 Berberis replicata
B147 Berberis replicata
B114 Berberis replicata
B116 Berberis replicata
B181 Berberis pachyacantha
B180 Berberis pachyacantha
B184 Berberis pachyacantha
B179 Berberis pachyacantha
B124 Berberis angulosa
B183 Berberis jaeschkeana
B176 Berberis jaeschkeana
B160 Berberis umbellata
B178 Berberis jaeschkeana
B185 Berberis jaeschkeana
B182 Berberis jaeschkeana
B158 Berberis umbellata
B110 Berberis glaucocarpa
B167 Berberis umbellata
B109 Berberis glaucocarpa
B55 Berberis aristata
B168 Berberis glaucocarpa
B107 Berberis glaucocarpa
B57 Berberis aristata
B108 Berberis glaucocarpa
B170 Berberis glaucocarpa
B171 Berberis glaucocarpa
B87 Berberis tinctoria
B34 Berberis chitria
B14 Berberis chitria
B175 Berberis chitria
B20 Berberis chitria
B100 Berberis tinctoria
B89 Berberis tinctoria
B93 Berberis wightiana
B92 Berberis tinctoria
B95 Berberis wightiana
B88 Berberis tinctoria
B90 Berberis tinctoria
B103 Berberis tinctoria
B94 Berberis wightiana
MP Tree
B103 Berberis tinctoria
B90 Berberis tinctoria
B100 Berberis tinctoria
B88 Berberis tinctoria
B93 Berberis wightiana
B92 Berberis tinctoria
B95 Berberis wightiana
B94 Berberis wightiana
B175 Berberis chitria
B14 Berberis chitria
B20 Berberis chitria
B34 Berberis chitria
B57 Berberis aristata
B170 Berberis glaucocarpa
B171 Berberis glaucocarpa
B55 Berberis aristata
B168 Berberis glaucocarpa
B108 Berberis glaucocarpa
B107 Berberis glaucocarpa
B167 Berberis umbellata
B110 Berberis glaucocarpa
B87 Berberis tinctoria
B89 Berberis tinctoria
B109 Berberis glaucocarpa
B114 Berberis replicata
B116 Berberis replicata
B149 Berberis replicata
B147 Berberis replicata
B111 Berberis glaucocarpa
B124 Berberis angulosa
B160 Berberis umbellata
B176 Berberis jaeschkeana
B185 Berberis jaeschkeana
B178 Berberis jaeschkeana
B183 Berberis jaeschkeana
B182 Berberis jaeschkeana
B158 Berberis umbellata
B1 Berberis lycium
B2 Berberis lycium
B3 Berberis lycium
B157 Berberis hainesii
B39 Berberis asiatica
B42 Berberis asiatica
B50 Berberis asiatica
B28 Berberis asiatica
B27 Berberis asiatica
B33 Berberis chitria
B151 Berberis hainesii
B5 Berberis lycium
B153 Berberis hainesii
B6 Berberis lycium
B152 Berberis hainesii
B4 Berberis lycium
B179 Berberis pachyacantha
B184 Berberis pachyacantha
B181 Berberis pachyacantha
B180 Berberis pachyacantha
B45 Berberis asiatica
UPGMA Tree
