# Supplementary material for: Universal Plant DNA Barcode Loci May Not Work in Complex Groups: A Case Study with Indian Berberis Species
Source: PLoS One. 2010 Oct 27;5(10):e13674. doi: 10.1371/journal.pone.0013674 (PMC2965122; doi:10.1371/journal.pone.0013674)
Supplement: Figure S4 — Strict consensus NJ, MP, UPGMA trees based on 58 common sequences of four loci in Berberis. (A) ITS, (B) matK, (C) rbcL and (D) trnH-psbA. Other details are, as in Figure S2. (0.09 MB PDF) [file pone.0013674.s004.pdf]

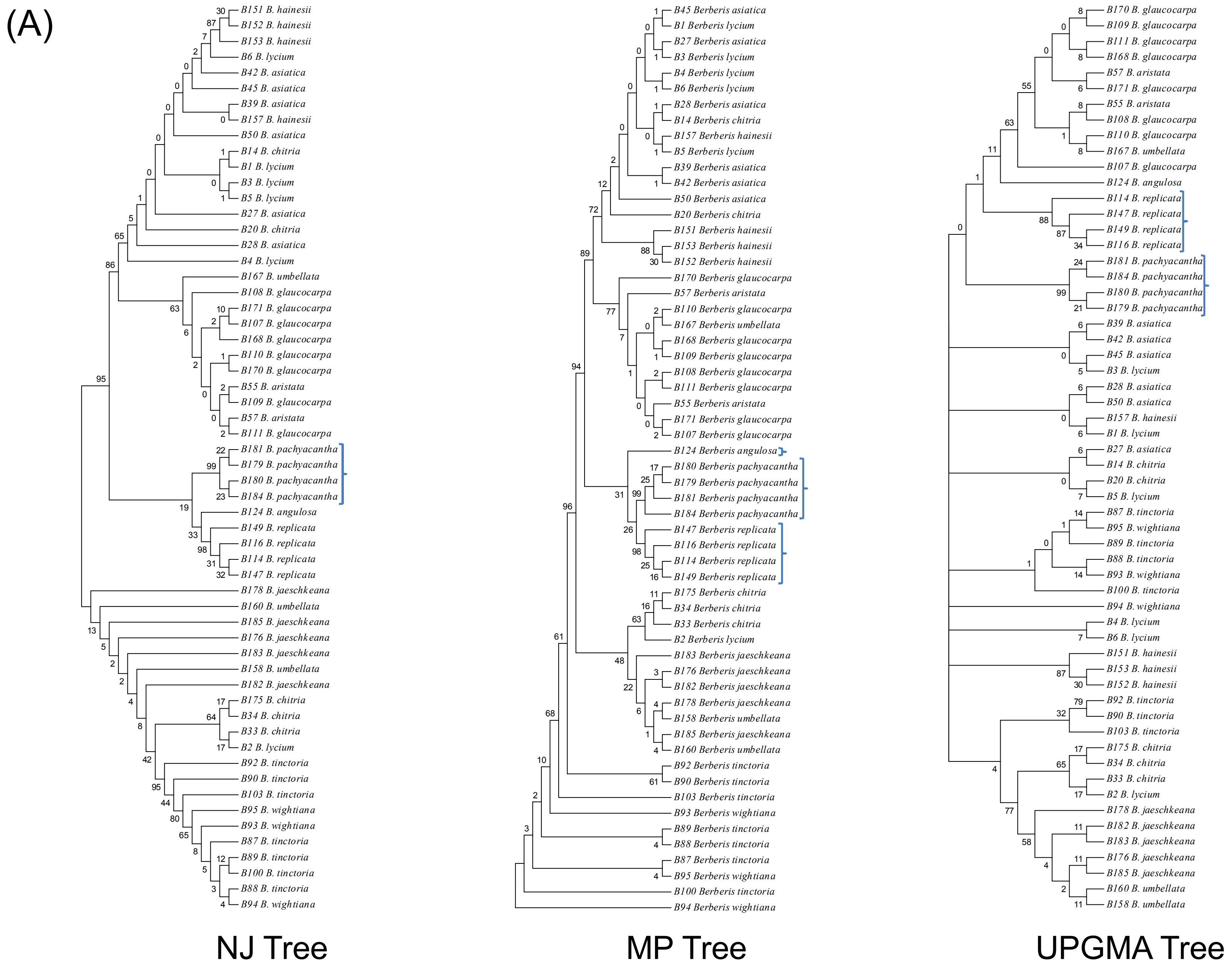
(A)
B151 B. hainesii
B152 B. hainesii
B153 B. hainesii
B6 B. lycium
B42 B. asiatica
B45 B. asiatica
B39 B. asiatica
B157 B. hainesii
B50 B. asiatica
B14 B. chitria
B1 B. lycium
B3 B. lycium
B5 B. lycium
B27 B. asiatica
B20 B. chitria
B28 B. asiatica
B4 B. lycium
B167 B. umbellata
B108 B. glaucocarpa
B171 B. glaucocarpa
B107 B. glaucocarpa
B168 B. glaucocarpa
B110 B. glaucocarpa
B170 B. glaucocarpa
B55 B. aristata
B109 B. glaucocarpa
B57 B. aristata
B111 B. glaucocarpa
B181 B. pachyacantha
B179 B. pachyacantha
B180 B. pachyacantha
B184 B. pachyacantha
B124 B. angulosa
B149 B. replicata
B116 B. replicata
B114 B. replicata
B147 B. replicata
B178 B. jaeschkeana
B160 B. umbellata
B185 B. jaeschkeana
B176 B. jaeschkeana
B183 B. jaeschkeana
B158 B. umbellata
B182 B. jaeschkeana
B175 B. chitria
B34 B. chitria
B33 B. chitria
B2 B. lycium
B92 B. tinctoria
B90 B. tinctoria
B103 B. tinctoria
B95 B. wightiana
B93 B. wightiana
B87 B. tinctoria
B89 B. tinctoria
B100 B. tinctoria
B88 B. tinctoria
B94 B. wightiana
NJ Tree
B45 Berberis asiatica
B1 Berberis lycium
B27 Berberis asiatica
B3 Berberis lycium
B4 Berberis lycium
B6 Berberis lycium
B28 Berberis asiatica
B14 Berberis chitria
B157 Berberis hainesii
B5 Berberis lycium
B39 Berberis asiatica
B42 Berberis asiatica
B50 Berberis asiatica
B20 Berberis chitria
B151 Berberis hainesii
B153 Berberis hainesii
B152 Berberis hainesii
B170 Berberis glaucocarpa
B57 Berberis aristata
B110 Berberis glaucocarpa
B167 Berberis umbellata
B168 Berberis glaucocarpa
B109 Berberis glaucocarpa
B108 Berberis glaucocarpa
B111 Berberis glaucocarpa
B55 Berberis aristata
B171 Berberis glaucocarpa
B107 Berberis glaucocarpa
B124 Berberis angulosa
B180 Berberis pachyacantha
B179 Berberis pachyacantha
B181 Berberis pachyacantha
B184 Berberis pachyacantha
B147 Berberis replicata
B116 Berberis replicata
B114 Berberis replicata
B149 Berberis replicata
B175 Berberis chitria
B34 Berberis chitria
B33 Berberis chitria
B2 Berberis lycium
B183 Berberis jaeschkeana
B176 Berberis jaeschkeana
B182 Berberis jaeschkeana
B178 Berberis jaeschkeana
B158 Berberis umbellata
B185 Berberis jaeschkeana
B160 Berberis umbellata
B92 Berberis tinctoria
B90 Berberis tinctoria
B103 Berberis tinctoria
B93 Berberis wightiana
B89 Berberis tinctoria
B88 Berberis tinctoria
B87 Berberis tinctoria
B95 Berberis wightiana
B100 Berberis tinctoria
B94 Berberis wightiana
MP Tree
B170 B. glaucocarpa
B109 B. glaucocarpa
B111 B. glaucocarpa
B168 B. glaucocarpa
B57 B. aristata
B171 B. glaucocarpa
B55 B. aristata
B108 B. glaucocarpa
B110 B. glaucocarpa
B167 B. umbellata
B107 B. glaucocarpa
B124 B. angulosa
B114 B. replicata
B147 B. replicata
B149 B. replicata
B116 B. replicata
B181 B. pachyacantha
B184 B. pachyacantha
B180 B. pachyacantha
B179 B. pachyacantha
B39 B. asiatica
B42 B. asiatica
B45 B. asiatica
B3 B. lycium
B28 B. asiatica
B50 B. asiatica
B157 B. hainesii
B1 B. lycium
B27 B. asiatica
B14 B. chitria
B20 B. chitria
B5 B. lycium
B87 B. tinctoria
B95 B. wightiana
B89 B. tinctoria
B88 B. tinctoria
B93 B. wightiana
B100 B. tinctoria
B94 B. wightiana
B4 B. lycium
B6 B. lycium
B151 B. hainesii
B153 B. hainesii
B152 B. hainesii
B92 B. tinctoria
B90 B. tinctoria
B103 B. tinctoria
B175 B. chitria
B34 B. chitria
B33 B. chitria
B2 B. lycium
B178 B. jaeschkeana
B182 B. jaeschkeana
B183 B. jaeschkeana
B176 B. jaeschkeana
B185 B. jaeschkeana
B160 B. umbellata
B158 B. umbellata
UPGMA Tree

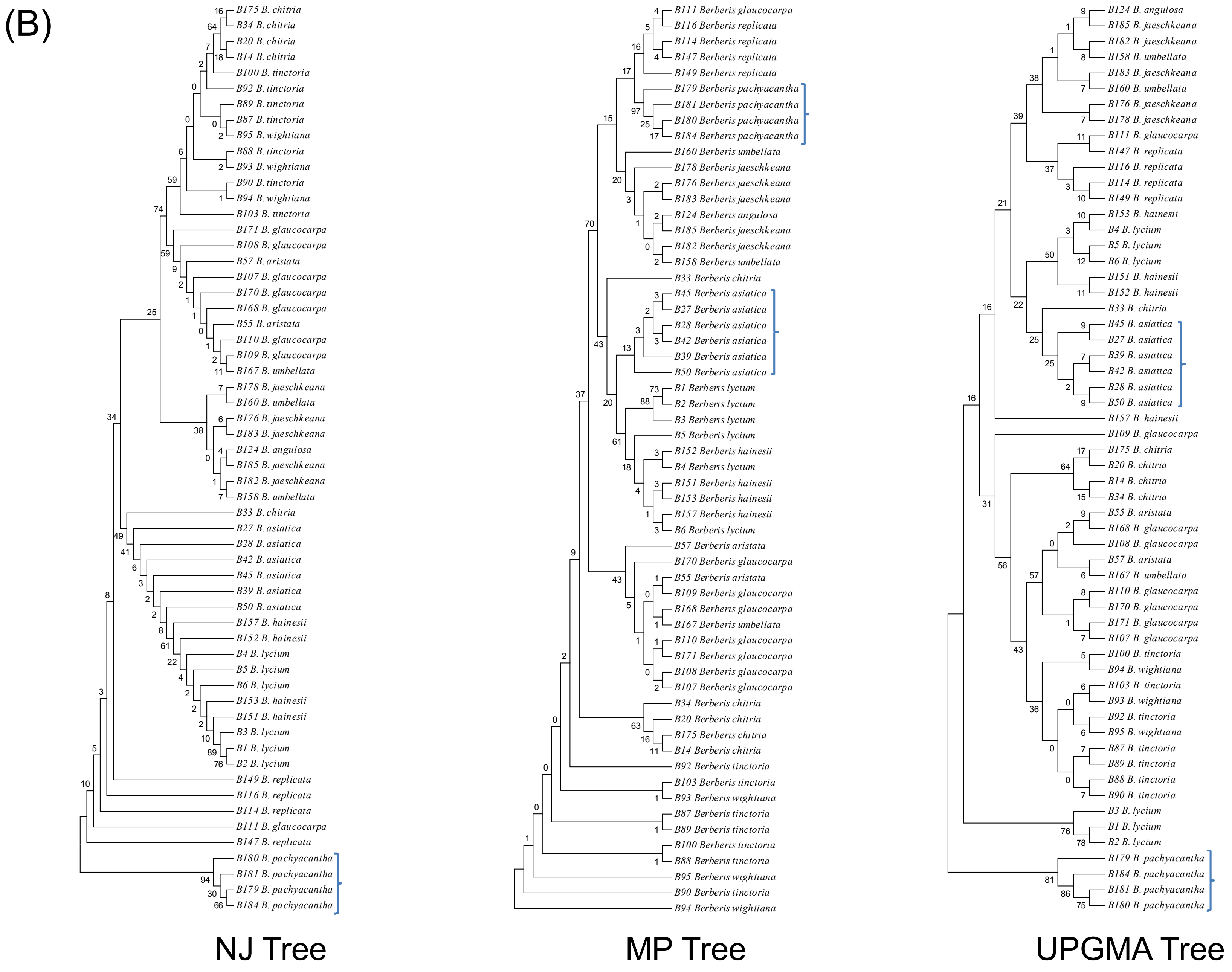
(B)
B175 B. chitria
B34 B. chitria
B20 B. chitria
B14 B. chitria
B100 B. tinctoria
B92 B. tinctoria
B89 B. tinctoria
B87 B. tinctoria
B95 B. wightiana
B88 B. tinctoria
B93 B. wightiana
B90 B. tinctoria
B94 B. wightiana
B103 B. tinctoria
B171 B. glaucocarpa
B108 B. glaucocarpa
B57 B. aristata
B107 B. glaucocarpa
B170 B. glaucocarpa
B168 B. glaucocarpa
B55 B. aristata
B110 B. glaucocarpa
B109 B. glaucocarpa
B167 B. umbellata
B178 B. jaeschkeana
B160 B. umbellata
B176 B. jaeschkeana
B183 B. jaeschkeana
B124 B. angulosa
B185 B. jaeschkeana
B182 B. jaeschkeana
B158 B. umbellata
B33 B. chitria
B27 B. asiatica
B28 B. asiatica
B42 B. asiatica
B45 B. asiatica
B39 B. asiatica
B50 B. asiatica
B157 B. hainesii
B152 B. hainesii
B4 B. lycium
B5 B. lycium
B6 B. lycium
B153 B. hainesii
B151 B. hainesii
B3 B. lycium
B1 B. lycium
B2 B. lycium
B149 B. replicata
B116 B. replicata
B114 B. replicata
B111 B. glaucocarpa
B147 B. replicata
B180 B. pachyacantha
B181 B. pachyacantha
B179 B. pachyacantha
B184 B. pachyacantha
NJ Tree
B111 Berberis glaucocarpa
B116 Berberis replicata
B114 Berberis replicata
B147 Berberis replicata
B149 Berberis replicata
B179 Berberis pachyacantha
B181 Berberis pachyacantha
B180 Berberis pachyacantha
B184 Berberis pachyacantha
B160 Berberis umbellata
B178 Berberis jaeschkeana
B176 Berberis jaeschkeana
B183 Berberis jaeschkeana
B124 Berberis angulosa
B185 Berberis jaeschkeana
B182 Berberis jaeschkeana
B158 Berberis umbellata
B33 Berberis chitria
B45 Berberis asiatica
B27 Berberis asiatica
B28 Berberis asiatica
B42 Berberis asiatica
B39 Berberis asiatica
B50 Berberis asiatica
B1 Berberis lycium
B2 Berberis lycium
B3 Berberis lycium
B5 Berberis lycium
B152 Berberis hainesii
B4 Berberis lycium
B151 Berberis hainesii
B153 Berberis hainesii
B157 Berberis hainesii
B6 Berberis lycium
B57 Berberis aristata
B170 Berberis glaucocarpa
B55 Berberis aristata
B109 Berberis glaucocarpa
B168 Berberis glaucocarpa
B167 Berberis umbellata
B110 Berberis glaucocarpa
B171 Berberis glaucocarpa
B108 Berberis glaucocarpa
B107 Berberis glaucocarpa
B34 Berberis chitria
B20 Berberis chitria
B175 Berberis chitria
B14 Berberis chitria
B92 Berberis tinctoria
B103 Berberis tinctoria
B93 Berberis wightiana
B87 Berberis tinctoria
B89 Berberis tinctoria
B100 Berberis tinctoria
B88 Berberis tinctoria
B95 Berberis wightiana
B90 Berberis tinctoria
B94 Berberis wightiana
MP Tree
B124 B. angulosa
B185 B. jaeschkeana
B182 B. jaeschkeana
B158 B. umbellata
B183 B. jaeschkeana
B160 B. umbellata
B176 B. jaeschkeana
B178 B. jaeschkeana
B111 B. glaucocarpa
B147 B. replicata
B116 B. replicata
B114 B. replicata
B149 B. replicata
B153 B. hainesii
B4 B. lycium
B5 B. lycium
B6 B. lycium
B151 B. hainesii
B152 B. hainesii
B33 B. chitria
B45 B. asiatica
B27 B. asiatica
B39 B. asiatica
B42 B. asiatica
B28 B. asiatica
B50 B. asiatica
B157 B. hainesii
B109 B. glaucocarpa
B175 B. chitria
B20 B. chitria
B14 B. chitria
B34 B. chitria
B55 B. aristata
B168 B. glaucocarpa
B108 B. glaucocarpa
B57 B. aristata
B167 B. umbellata
B110 B. glaucocarpa
B170 B. glaucocarpa
B171 B. glaucocarpa
B107 B. glaucocarpa
B100 B. tinctoria
B94 B. wightiana
B103 B. tinctoria
B93 B. wightiana
B92 B. tinctoria
B95 B. wightiana
B87 B. tinctoria
B89 B. tinctoria
B88 B. tinctoria
B90 B. tinctoria
B3 B. lycium
B1 B. lycium
B2 B. lycium
B179 B. pachyacantha
B184 B. pachyacantha
B181 B. pachyacantha
B180 B. pachyacantha
UPGMA Tree

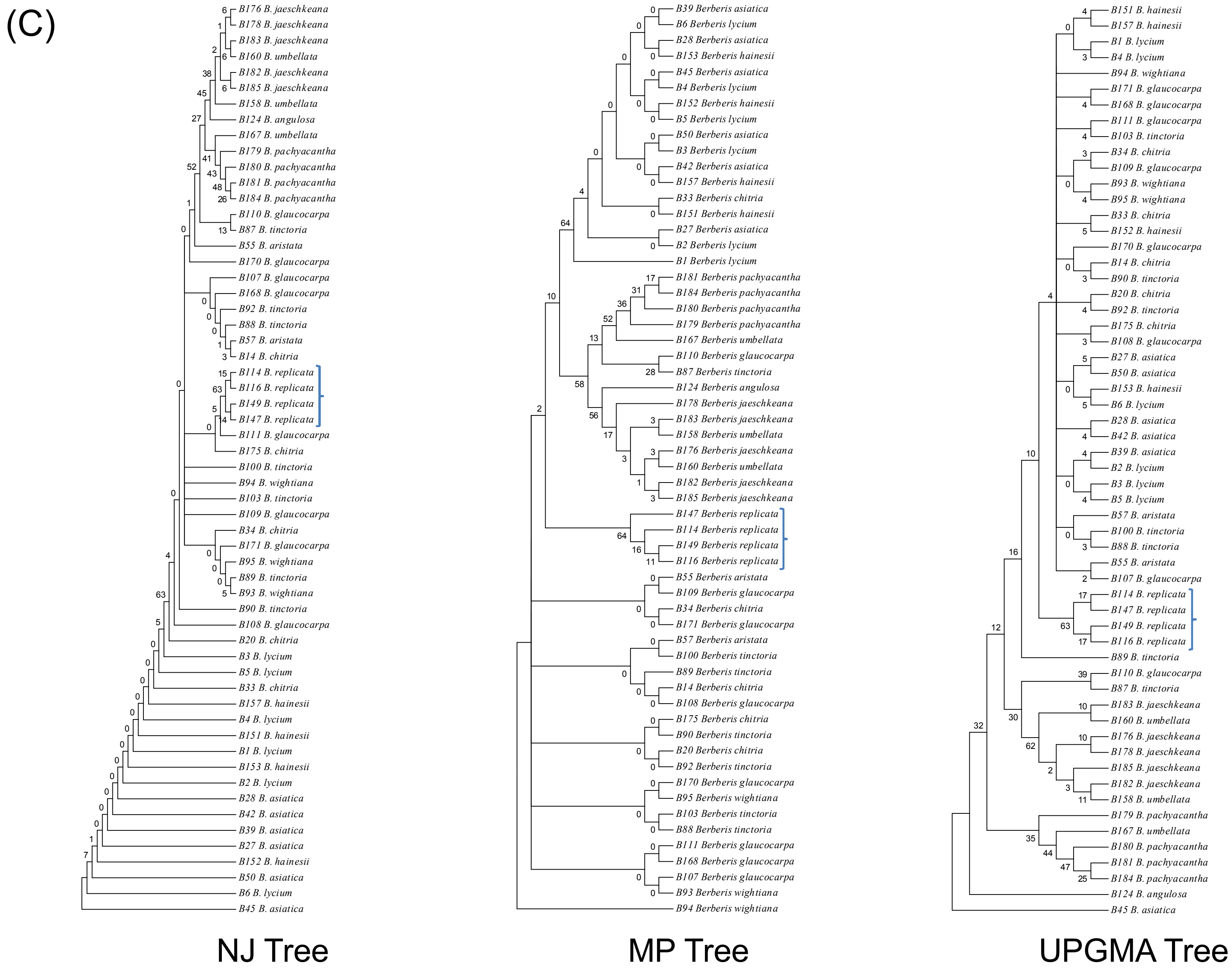
(C)
B176 B. jaeschkeana
B178 B. jaeschkeana
B183 B. jaeschkeana
B160 B. umbellata
B182 B. jaeschkeana
B185 B. jaeschkeana
B158 B. umbellata
B124 B. angulosa
B167 B. umbellata
B179 B. pachyacantha
B180 B. pachyacantha
B181 B. pachyacantha
B184 B. pachyacantha
B110 B. glaucocarpa
B87 B. tinctoria
B55 B. aristata
B170 B. glaucocarpa
B107 B. glaucocarpa
B168 B. glaucocarpa
B92 B. tinctoria
B88 B. tinctoria
B57 B. aristata
B14 B. chitria
B114 B. replicata
B116 B. replicata
B149 B. replicata
B147 B. replicata
B111 B. glaucocarpa
B175 B. chitria
B100 B. tinctoria
B94 B. wightiana
B103 B. tinctoria
B109 B. glaucocarpa
B34 B. chitria
B171 B. glaucocarpa
B95 B. wightiana
B89 B. tinctoria
B93 B. wightiana
B90 B. tinctoria
B108 B. glaucocarpa
B20 B. chitria
B3 B. lycium
B5 B. lycium
B33 B. chitria
B157 B. hainesii
B4 B. lycium
B151 B. hainesii
B1 B. lycium
B153 B. hainesii
B2 B. lycium
B28 B. asiatica
B42 B. asiatica
B39 B. asiatica
B27 B. asiatica
B152 B. hainesii
B50 B. asiatica
B6 B. lycium
B45 B. asiatica
NJ Tree
B39 Berberis asiatica
B6 Berberis lycium
B28 Berberis asiatica
B153 Berberis hainesii
B45 Berberis asiatica
B4 Berberis lycium
B152 Berberis hainesii
B5 Berberis lycium
B50 Berberis asiatica
B3 Berberis lycium
B42 Berberis asiatica
B157 Berberis hainesii
B33 Berberis chitria
B151 Berberis hainesii
B27 Berberis asiatica
B2 Berberis lycium
B1 Berberis lycium
B181 Berberis pachyacantha
B184 Berberis pachyacantha
B180 Berberis pachyacantha
B179 Berberis pachyacantha
B167 Berberis umbellata
B110 Berberis glaucocarpa
B87 Berberis tinctoria
B124 Berberis angulosa
B178 Berberis jaeschkeana
B183 Berberis jaeschkeana
B158 Berberis umbellata
B176 Berberis jaeschkeana
B160 Berberis umbellata
B182 Berberis jaeschkeana
B185 Berberis jaeschkeana
B147 Berberis replicata
B114 Berberis replicata
B149 Berberis replicata
B116 Berberis replicata
B55 Berberis aristata
B109 Berberis glaucocarpa
B34 Berberis chitria
B171 Berberis glaucocarpa
B57 Berberis aristata
B100 Berberis tinctoria
B89 Berberis tinctoria
B14 Berberis chitria
B108 Berberis glaucocarpa
B175 Berberis chitria
B90 Berberis tinctoria
B20 Berberis chitria
B92 Berberis tinctoria
B170 Berberis glaucocarpa
B95 Berberis wightiana
B103 Berberis tinctoria
B88 Berberis tinctoria
B111 Berberis glaucocarpa
B168 Berberis glaucocarpa
B107 Berberis glaucocarpa
B93 Berberis wightiana
B94 Berberis wightiana
MP Tree
B151 B. hainesii
B157 B. hainesii
B1 B. lycium
B4 B. lycium
B94 B. wightiana
B171 B. glaucocarpa
B168 B. glaucocarpa
B111 B. glaucocarpa
B103 B. tinctoria
B34 B. chitria
B109 B. glaucocarpa
B93 B. wightiana
B95 B. wightiana
B33 B. chitria
B152 B. hainesii
B170 B. glaucocarpa
B14 B. chitria
B90 B. tinctoria
B20 B. chitria
B92 B. tinctoria
B175 B. chitria
B108 B. glaucocarpa
B27 B. asiatica
B50 B. asiatica
B153 B. hainesii
B6 B. lycium
B28 B. asiatica
B42 B. asiatica
B39 B. asiatica
B2 B. lycium
B3 B. lycium
B5 B. lycium
B57 B. aristata
B100 B. tinctoria
B88 B. tinctoria
B55 B. aristata
B107 B. glaucocarpa
B114 B. replicata
B147 B. replicata
B149 B. replicata
B116 B. replicata
B89 B. tinctoria
B110 B. glaucocarpa
B87 B. tinctoria
B183 B. jaeschkeana
B160 B. umbellata
B176 B. jaeschkeana
B178 B. jaeschkeana
B185 B. jaeschkeana
B182 B. jaeschkeana
B158 B. umbellata
B179 B. pachyacantha
B167 B. umbellata
B180 B. pachyacantha
B181 B. pachyacantha
B184 B. pachyacantha
B124 B. angulosa
B45 B. asiatica
UPGMA Tree

(D)

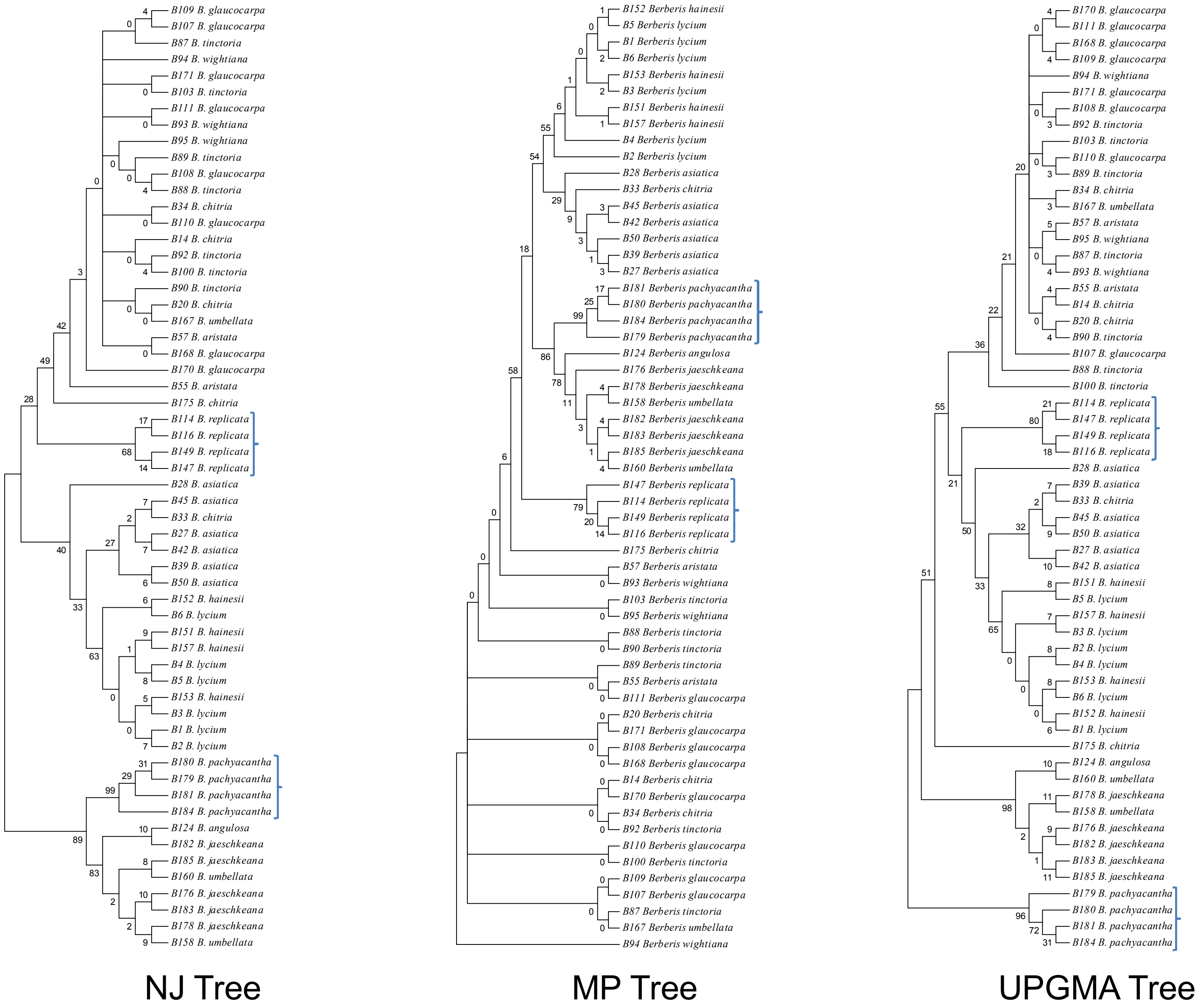
B109 B. glaucocarpa
B107 B. glaucocarpa
B87 B. tinctoria
B94 B. wightiana
B171 B. glaucocarpa
B103 B. tinctoria
B111 B. glaucocarpa
B93 B. wightiana
B95 B. wightiana
B89 B. tinctoria
B108 B. glaucocarpa
B88 B. tinctoria
B34 B. chitria
B110 B. glaucocarpa
B14 B. chitria
B92 B. tinctoria
B100 B. tinctoria
B90 B. tinctoria
B20 B. chitria
B167 B. umbellata
B57 B. aristata
B168 B. glaucocarpa
B170 B. glaucocarpa
B55 B. aristata
B175 B. chitria
B114 B. replicata
B116 B. replicata
B149 B. replicata
B147 B. replicata
B28 B. asiatica
B45 B. asiatica
B33 B. chitria
B27 B. asiatica
B42 B. asiatica
B39 B. asiatica
B50 B. asiatica
B152 B. hainesii
B6 B. lycium
B151 B. hainesii
B157 B. hainesii
B4 B. lycium
B5 B. lycium
B153 B. hainesii
B3 B. lycium
B1 B. lycium
B2 B. lycium
B180 B. pachyacantha
B179 B. pachyacantha
B181 B. pachyacantha
B184 B. pachyacantha
B124 B. angulosa
B182 B. jaeschkeana
B185 B. jaeschkeana
B160 B. umbellata
B176 B. jaeschkeana
B183 B. jaeschkeana
B178 B. jaeschkeana
B158 B. umbellata
NJ Tree
B152 Berberis hainesii
B5 Berberis lycium
B1 Berberis lycium
B6 Berberis lycium
B153 Berberis hainesii
B3 Berberis lycium
B151 Berberis hainesii
B157 Berberis hainesii
B4 Berberis lycium
B2 Berberis lycium
B28 Berberis asiatica
B33 Berberis chitria
B45 Berberis asiatica
B42 Berberis asiatica
B50 Berberis asiatica
B39 Berberis asiatica
B27 Berberis asiatica
B181 Berberis pachyacantha
B180 Berberis pachyacantha
B184 Berberis pachyacantha
B179 Berberis pachyacantha
B124 Berberis angulosa
B176 Berberis jaeschkeana
B178 Berberis jaeschkeana
B158 Berberis umbellata
B182 Berberis jaeschkeana
B183 Berberis jaeschkeana
B185 Berberis jaeschkeana
B160 Berberis umbellata
B147 Berberis replicata
B114 Berberis replicata
B149 Berberis replicata
B116 Berberis replicata
B175 Berberis chitria
B57 Berberis aristata
B93 Berberis wightiana
B103 Berberis tinctoria
B95 Berberis wightiana
B88 Berberis tinctoria
B90 Berberis tinctoria
B89 Berberis tinctoria
B55 Berberis aristata
B111 Berberis glaucocarpa
B20 Berberis chitria
B171 Berberis glaucocarpa
B108 Berberis glaucocarpa
B168 Berberis glaucocarpa
B14 Berberis chitria
B170 Berberis glaucocarpa
B34 Berberis chitria
B92 Berberis tinctoria
B110 Berberis glaucocarpa
B100 Berberis tinctoria
B109 Berberis glaucocarpa
B107 Berberis glaucocarpa
B87 Berberis tinctoria
B167 Berberis umbellata
B94 Berberis wightiana
MP Tree
B170 B. glaucocarpa
B111 B. glaucocarpa
B168 B. glaucocarpa
B109 B. glaucocarpa
B94 B. wightiana
B171 B. glaucocarpa
B108 B. glaucocarpa
B92 B. tinctoria
B103 B. tinctoria
B110 B. glaucocarpa
B89 B. tinctoria
B34 B. chitria
B167 B. umbellata
B57 B. aristata
B95 B. wightiana
B87 B. tinctoria
B93 B. wightiana
B55 B. aristata
B14 B. chitria
B20 B. chitria
B90 B. tinctoria
B107 B. glaucocarpa
B88 B. tinctoria
B100 B. tinctoria
B114 B. replicata
B147 B. replicata
B149 B. replicata
B116 B. replicata
B28 B. asiatica
B39 B. asiatica
B33 B. chitria
B45 B. asiatica
B50 B. asiatica
B27 B. asiatica
B42 B. asiatica
B151 B. hainesii
B5 B. lycium
B157 B. hainesii
B3 B. lycium
B2 B. lycium
B4 B. lycium
B153 B. hainesii
B6 B. lycium
B152 B. hainesii
B1 B. lycium
B175 B. chitria
B124 B. angulosa
B160 B. umbellata
B178 B. jaeschkeana
B158 B. umbellata
B176 B. jaeschkeana
B182 B. jaeschkeana
B183 B. jaeschkeana
B185 B. jaeschkeana
B179 B. pachyacantha
B180 B. pachyacantha
B181 B. pachyacantha
B184 B. pachyacantha
UPGMA Tree
