# Supplementary material for: Universal Plant DNA Barcode Loci May Not Work in Complex Groups: A Case Study with Indian Berberis Species
Source: PLoS One. 2010 Oct 27;5(10):e13674. doi: 10.1371/journal.pone.0013674 (PMC2965122; doi:10.1371/journal.pone.0013674)
Supplement: Figure S5 — Strict consensus NJ, MP, UPGMA trees based on ITS (A), matK (B), rbcL (C) and trnH-psbA (D) sequences of different species of Ficus. F.elastica and F.rumphi were represented by only one accession with trnH-psbA sequences. (0.10 MB PDF) [file pone.0013674.s005.pdf]

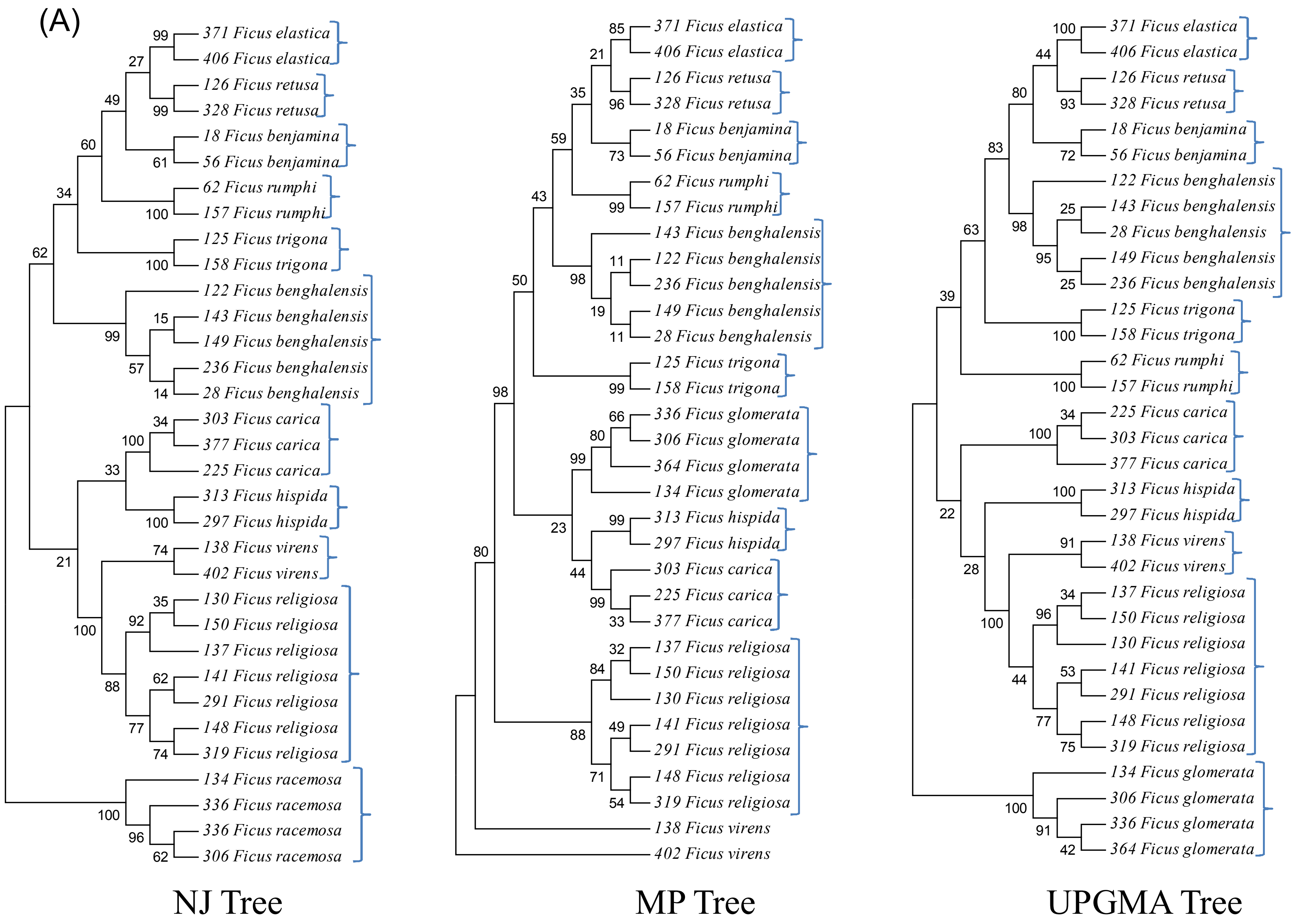

(A)
371 Ficus elastica
406 Ficus elastica
126 Ficus retusa
328 Ficus retusa
18 Ficus benjamina
56 Ficus benjamina
62 Ficus rumphi
157 Ficus rumphi
125 Ficus trigona
158 Ficus trigona
122 Ficus benghalensis
143 Ficus benghalensis
149 Ficus benghalensis
236 Ficus benghalensis
28 Ficus benghalensis
303 Ficus carica
377 Ficus carica
225 Ficus carica
313 Ficus hispida
297 Ficus hispida
138 Ficus virens
402 Ficus virens
130 Ficus religiosa
150 Ficus religiosa
137 Ficus religiosa
141 Ficus religiosa
291 Ficus religiosa
148 Ficus religiosa
319 Ficus religiosa
134 Ficus racemosa
336 Ficus racemosa
336 Ficus racemosa
306 Ficus racemosa
NJ Tree
371 Ficus elastica
406 Ficus elastica
126 Ficus retusa
328 Ficus retusa
18 Ficus benjamina
56 Ficus benjamina
62 Ficus rumphi
157 Ficus rumphi
143 Ficus benghalensis
122 Ficus benghalensis
236 Ficus benghalensis
149 Ficus benghalensis
28 Ficus benghalensis
125 Ficus trigona
158 Ficus trigona
336 Ficus glomerata
306 Ficus glomerata
364 Ficus glomerata
134 Ficus glomerata
313 Ficus hispida
297 Ficus hispida
303 Ficus carica
225 Ficus carica
377 Ficus carica
137 Ficus religiosa
150 Ficus religiosa
130 Ficus religiosa
141 Ficus religiosa
291 Ficus religiosa
148 Ficus religiosa
319 Ficus religiosa
138 Ficus virens
402 Ficus virens
MP Tree
371 Ficus elastica
406 Ficus elastica
126 Ficus retusa
328 Ficus retusa
18 Ficus benjamina
56 Ficus benjamina
122 Ficus benghalensis
143 Ficus benghalensis
28 Ficus benghalensis
149 Ficus benghalensis
236 Ficus benghalensis
125 Ficus trigona
158 Ficus trigona
62 Ficus rumphi
157 Ficus rumphi
225 Ficus carica
303 Ficus carica
377 Ficus carica
313 Ficus hispida
297 Ficus hispida
138 Ficus virens
402 Ficus virens
137 Ficus religiosa
150 Ficus religiosa
130 Ficus religiosa
141 Ficus religiosa
291 Ficus religiosa
148 Ficus religiosa
319 Ficus religiosa
134 Ficus glomerata
306 Ficus glomerata
336 Ficus glomerata
364 Ficus glomerata
UPGMA Tree

(B)

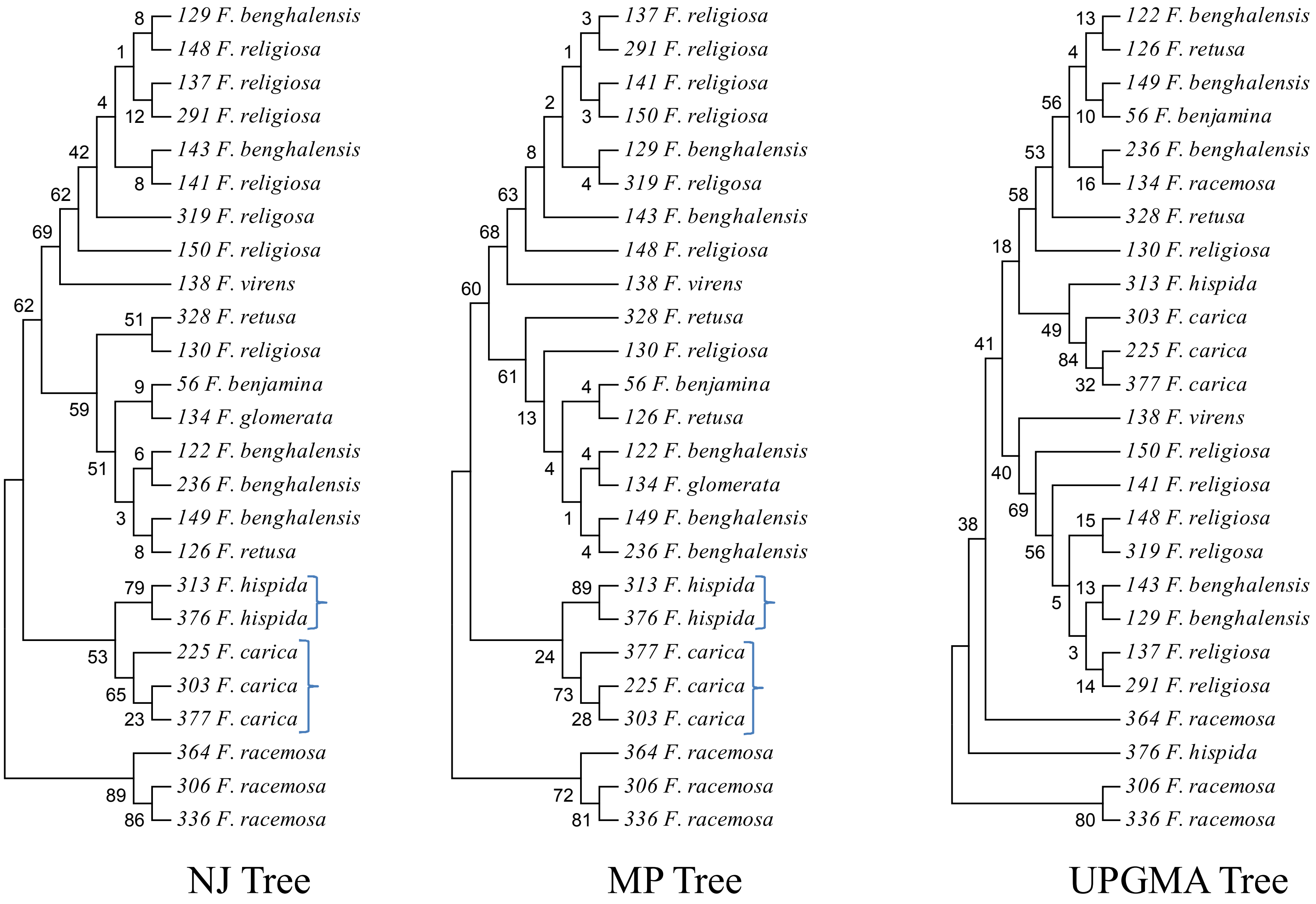
129 F. benghalensis
148 F. religiosa
137 F. religiosa
291 F. religiosa
143 F. benghalensis
141 F. religiosa
319 F. religosa
150 F. religiosa
138 F. virens
328 F. retusa
130 F. religiosa
56 F. benjamina
134 F. glomerata
122 F. benghalensis
236 F. benghalensis
149 F. benghalensis
126 F. retusa
313 F. hispida
376 F. hispida
225 F. carica
303 F. carica
377 F. carica
364 F. racemosa
306 F. racemosa
336 F. racemosa
NJ Tree
137 F. religiosa
291 F. religiosa
141 F. religiosa
150 F. religiosa
129 F. benghalensis
319 F. religosa
143 F. benghalensis
148 F. religiosa
138 F. virens
328 F. retusa
130 F. religiosa
56 F. benjamina
126 F. retusa
122 F. benghalensis
134 F. glomerata
149 F. benghalensis
236 F. benghalensis
313 F. hispida
376 F. hispida
377 F. carica
225 F. carica
303 F. carica
364 F. racemosa
306 F. racemosa
336 F. racemosa
MP Tree
122 F. benghalensis
126 F. retusa
149 F. benghalensis
56 F. benjamina
236 F. benghalensis
134 F. racemosa
328 F. retusa
130 F. religiosa
313 F. hispida
303 F. carica
225 F. carica
377 F. carica
138 F. virens
150 F. religiosa
141 F. religiosa
148 F. religiosa
319 F. religosa
143 F. benghalensis
129 F. benghalensis
137 F. religiosa
291 F. religiosa
364 F. racemosa
376 F. hispida
306 F. racemosa
336 F. racemosa
UPGMA Tree

(C)

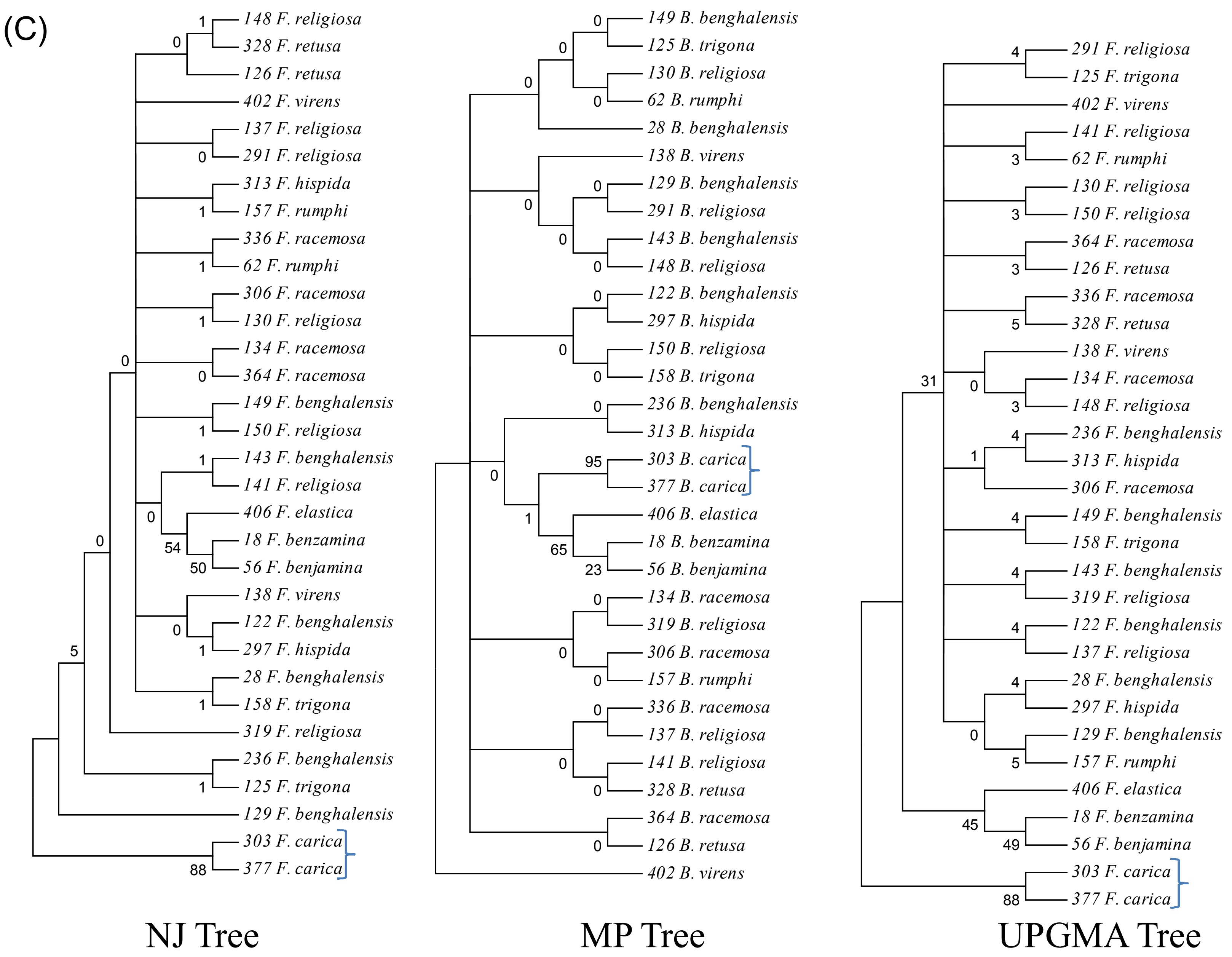
148 F. religiosa
328 F. retusa
126 F. retusa
402 F. virens
137 F. religiosa
291 F. religiosa
313 F. hispida
157 F. rumphi
336 F. racemosa
62 F. rumphi
306 F. racemosa
130 F. religiosa
134 F. racemosa
364 F. racemosa
149 F. benghalensis
150 F. religiosa
143 F. benghalensis
141 F. religiosa
406 F. elastica
18 F. benzamina
56 F. benjamina
138 F. virens
122 F. benghalensis
297 F. hispida
28 F. benghalensis
158 F. trigona
319 F. religiosa
236 F. benghalensis
125 F. trigona
129 F. benghalensis
303 F. carica
377 F. carica
NJ Tree
149 B. benghalensis
125 B. trigona
130 B. religiosa
62 B. rumphi
28 B. benghalensis
138 B. virens
129 B. benghalensis
291 B. religiosa
143 B. benghalensis
148 B. religiosa
122 B. benghalensis
297 B. hispida
150 B. religiosa
158 B. trigona
236 B. benghalensis
313 B. hispida
303 B. carica
377 B. carica
406 B. elastica
18 B. benzamina
56 B. benjamina
134 B. racemosa
319 B. religiosa
306 B. racemosa
157 B. rumphi
336 B. racemosa
137 B. religiosa
141 B. religiosa
328 B. retusa
364 B. racemosa
126 B. retusa
402 B. virens
MP Tree
291 F. religiosa
125 F. trigona
402 F. virens
141 F. religiosa
62 F. rumphi
130 F. religiosa
150 F. religiosa
364 F. racemosa
126 F. retusa
336 F. racemosa
328 F. retusa
138 F. virens
134 F. racemosa
148 F. religiosa
236 F. benghalensis
313 F. hispida
306 F. racemosa
149 F. benghalensis
158 F. trigona
143 F. benghalensis
319 F. religiosa
122 F. benghalensis
137 F. religiosa
28 F. benghalensis
297 F. hispida
129 F. benghalensis
157 F. rumphi
406 F. elastica
18 F. benzamina
56 F. benjamina
303 F. carica
377 F. carica
UPGMA Tree

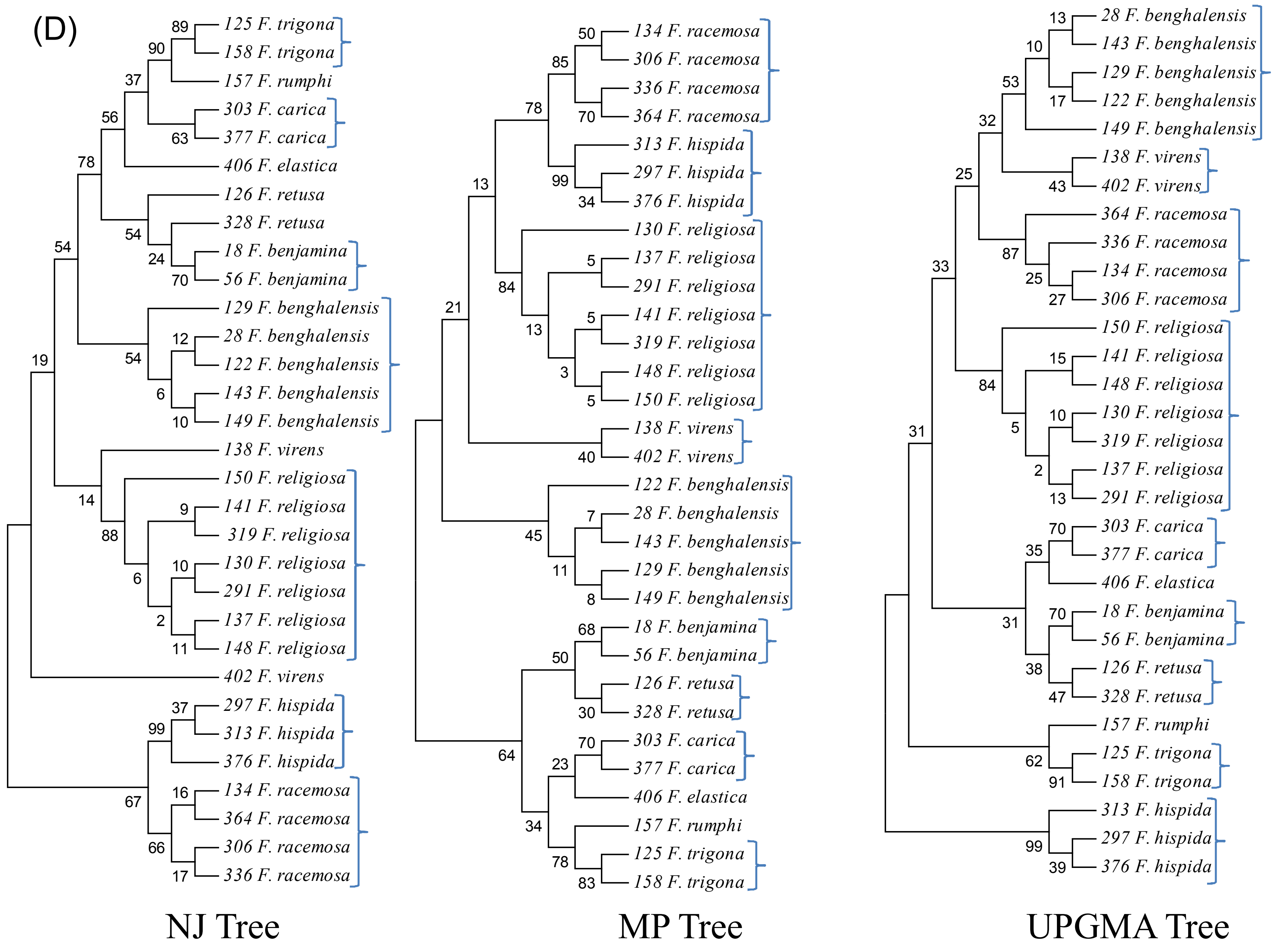

(D)
125 F. trigona
158 F. trigona
157 F. rumphi
303 F. carica
377 F. carica
406 F. elastica
126 F. retusa
328 F. retusa
18 F. benjamina
56 F. benjamina
129 F. benghalensis
28 F. benghalensis
122 F. benghalensis
143 F. benghalensis
149 F. benghalensis
138 F. virens
150 F. religiosa
141 F. religiosa
319 F. religiosa
130 F. religiosa
291 F. religiosa
137 F. religiosa
148 F. religiosa
402 F. virens
297 F. hispida
313 F. hispida
376 F. hispida
134 F. racemosa
364 F. racemosa
306 F. racemosa
336 F. racemosa
NJ Tree
134 F. racemosa
306 F. racemosa
336 F. racemosa
364 F. racemosa
313 F. hispida
297 F. hispida
376 F. hispida
130 F. religiosa
137 F. religiosa
291 F. religiosa
141 F. religiosa
319 F. religiosa
148 F. religiosa
150 F. religiosa
138 F. virens
402 F. virens
122 F. benghalensis
28 F. benghalensis
143 F. benghalensis
129 F. benghalensis
149 F. benghalensis
18 F. benjamina
56 F. benjamina
126 F. retusa
328 F. retusa
303 F. carica
377 F. carica
406 F. elastica
157 F. rumphi
125 F. trigona
158 F. trigona
MP Tree
28 F. benghalensis
143 F. benghalensis
129 F. benghalensis
122 F. benghalensis
149 F. benghalensis
138 F. virens
402 F. virens
364 F. racemosa
336 F. racemosa
134 F. racemosa
306 F. racemosa
150 F. religiosa
141 F. religiosa
148 F. religiosa
130 F. religiosa
319 F. religiosa
137 F. religiosa
291 F. religiosa
303 F. carica
377 F. carica
406 F. elastica
18 F. benjamina
56 F. benjamina
126 F. retusa
328 F. retusa
157 F. rumphi
125 F. trigona
158 F. trigona
313 F. hispida
297 F. hispida
376 F. hispida
UPGMA Tree
