# Supplementary material for: Universal Plant DNA Barcode Loci May Not Work in Complex Groups: A Case Study with Indian Berberis Species
Source: PLoS One. 2010 Oct 27;5(10):e13674. doi: 10.1371/journal.pone.0013674 (PMC2965122; doi:10.1371/journal.pone.0013674)
Supplement: Figure S6 — Strict consensus NJ, MP, UPGMA trees based on ITS (A), matK (B), rbcL (C) and trnH-psbA (D) sequences of different species of Gossypium. (0.08 MB PDF) [file pone.0013674.s006.pdf]

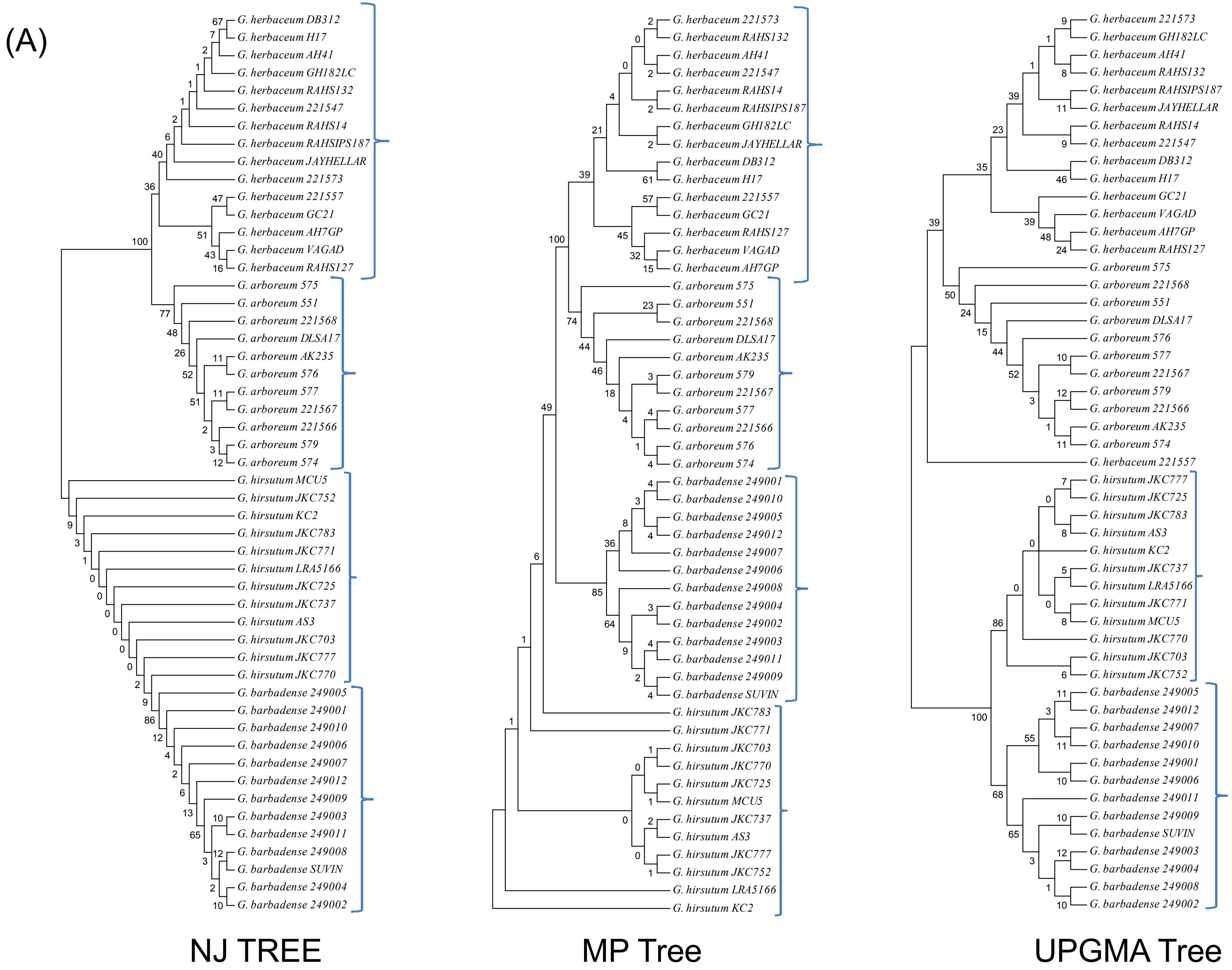
(A)
G. herbaceum DB312
G. herbaceum H17
G. herbaceum AH41
G. herbaceum GH182LC
G. herbaceum RAHS132
G. herbaceum 221547
G. herbaceum RAHS14
G. herbaceum RAHSIPS187
G. herbaceum JAYHELLAR
G. herbaceum 221573
G. herbaceum 221557
G. herbaceum GC21
G. herbaceum AH7GP
G. herbaceum VAGAD
G. herbaceum RAHS127
G. arboreum 575
G. arboreum 551
G. arboreum 221568
G. arboreum DLSA17
G. arboreum AK235
G. arboreum 576
G. arboreum 577
G. arboreum 221567
G. arboreum 221566
G. arboreum 579
G. arboreum 574
G. hirsutum MCU5
G. hirsutum JKC752
G. hirsutum KC2
G. hirsutum JKC783
G. hirsutum JKC771
G. hirsutum LRA5166
G. hirsutum JKC725
G. hirsutum JKC737
G. hirsutum AS3
G. hirsutum JKC703
G. hirsutum JKC777
G. hirsutum JKC770
G. barbadense 249005
G. barbadense 249001
G. barbadense 249010
G. barbadense 249006
G. barbadense 249007
G. barbadense 249012
G. barbadense 249009
G. barbadense 249003
G. barbadense 249011
G. barbadense 249008
G. barbadense SUVIN
G. barbadense 249004
G. barbadense 249002
NJ TREE
G. herbaceum 221573
G. herbaceum RAHS132
G. herbaceum AH41
G. herbaceum 221547
G. herbaceum RAHS14
G. herbaceum RAHSIPS187
G. herbaceum GH182LC
G. herbaceum JAYHELLAR
G. herbaceum DB312
G. herbaceum H17
G. herbaceum 221557
G. herbaceum GC21
G. herbaceum RAHS127
G. herbaceum VAGAD
G. herbaceum AH7GP
G. arboreum 575
G. arboreum 551
G. arboreum 221568
G. arboreum DLSA17
G. arboreum AK235
G. arboreum 579
G. arboreum 221567
G. arboreum 577
G. arboreum 221566
G. arboreum 576
G. arboreum 574
G. barbadense 249001
G. barbadense 249010
G. barbadense 249005
G. barbadense 249012
G. barbadense 249007
G. barbadense 249006
G. barbadense 249008
G. barbadense 249004
G. barbadense 249002
G. barbadense 249003
G. barbadense 249011
G. barbadense 249009
G. barbadense SUVIN
G. hirsutum JKC783
G. hirsutum JKC771
G. hirsutum JKC703
G. hirsutum JKC770
G. hirsutum JKC725
G. hirsutum MCU5
G. hirsutum JKC737
G. hirsutum AS3
G. hirsutum JKC777
G. hirsutum JKC752
G. hirsutum LRA5166
G. hirsutum KC2
MP Tree
G. herbaceum 221573
G. herbaceum GH182LC
G. herbaceum AH41
G. herbaceum RAHS132
G. herbaceum RAHSIPS187
G. herbaceum JAYHELLAR
G. herbaceum RAHS14
G. herbaceum 221547
G. herbaceum DB312
G. herbaceum H17
G. herbaceum GC21
G. herbaceum VAGAD
G. herbaceum AH7GP
G. herbaceum RAHS127
G. arboreum 575
G. arboreum 221568
G. arboreum 551
G. arboreum DLSA17
G. arboreum 576
G. arboreum 577
G. arboreum 221567
G. arboreum 579
G. arboreum 221566
G. arboreum AK235
G. arboreum 574
G. herbaceum 221557
G. hirsutum JKC777
G. hirsutum JKC725
G. hirsutum JKC783
G. hirsutum AS3
G. hirsutum KC2
G. hirsutum JKC737
G. hirsutum LRA5166
G. hirsutum JKC771
G. hirsutum MCU5
G. hirsutum JKC770
G. hirsutum JKC703
G. hirsutum JKC752
G. barbadense 249005
G. barbadense 249012
G. barbadense 249007
G. barbadense 249010
G. barbadense 249001
G. barbadense 249006
G. barbadense 249011
G. barbadense 249009
G. barbadense SUVIN
G. barbadense 249003
G. barbadense 249004
G. barbadense 249008
G. barbadense 249002
UPGMA Tree

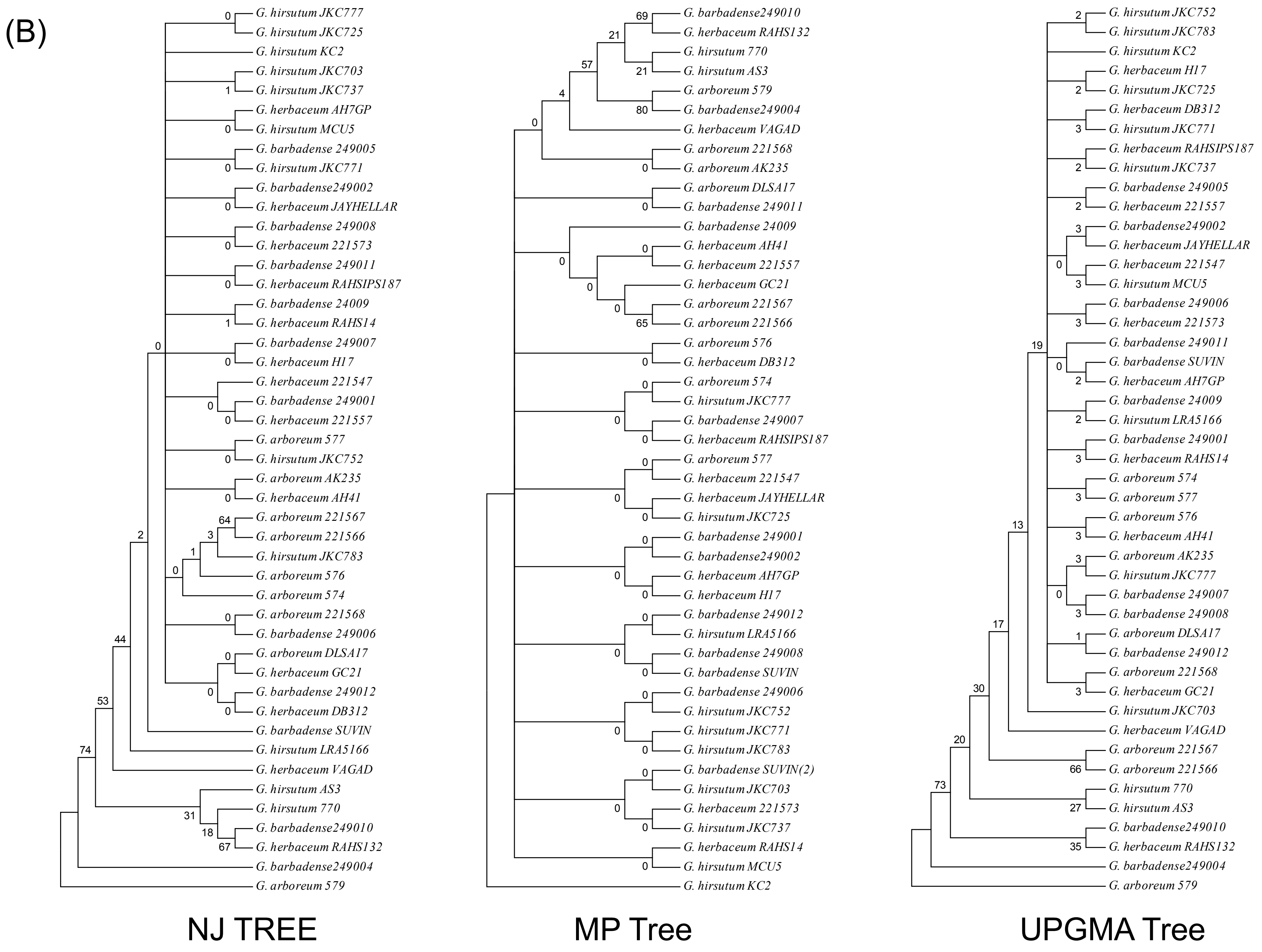

(B)
G. hirsutum JKC777
G. hirsutum JKC725
G. hirsutum KC2
G. hirsutum JKC703
G. hirsutum JKC737
G. herbaceum AH7GP
G. hirsutum MCU5
G. barbadense 249005
G. hirsutum JKC771
G. barbadense249002
G. herbaceum JAYHELLAR
G. barbadense 249008
G. herbaceum 221573
G. barbadense 249011
G. herbaceum RAHSIPS187
G. barbadense 24009
G. herbaceum RAHS14
G. barbadense 249007
G. herbaceum H17
G. herbaceum 221547
G. barbadense 249001
G. herbaceum 221557
G. arboreum 577
G. hirsutum JKC752
G. arboreum AK235
G. herbaceum AH41
G. arboreum 221567
G. arboreum 221566
G. hirsutum JKC783
G. arboreum 576
G. arboreum 574
G. arboreum 221568
G. barbadense 249006
G. arboreum DLSA17
G. herbaceum GC21
G. barbadense 249012
G. herbaceum DB312
G. barbadense SUVIN
G. hirsutum LRA5166
G. herbaceum VAGAD
G. hirsutum AS3
G. hirsutum 770
G. barbadense249010
G. herbaceum RAHS132
G. barbadense249004
G. arboreum 579
NJ TREE
G. barbadense249010
G. herbaceum RAHS132
G. hirsutum 770
G. hirsutum AS3
G. arboreum 579
G. barbadense249004
G. herbaceum VAGAD
G. arboreum 221568
G. arboreum AK235
G. arboreum DLSA17
G. barbadense 249011
G. barbadense 24009
G. herbaceum AH41
G. herbaceum 221557
G. herbaceum GC21
G. arboreum 221567
G. arboreum 221566
G. arboreum 576
G. herbaceum DB312
G. arboreum 574
G. hirsutum JKC777
G. barbadense 249007
G. herbaceum RAHSIPS187
G. arboreum 577
G. herbaceum 221547
G. herbaceum JAYHELLAR
G. hirsutum JKC725
G. barbadense 249001
G. barbadense249002
G. herbaceum AH7GP
G. herbaceum H17
G. barbadense 249012
G. hirsutum LRA5166
G. barbadense 249008
G. barbadense SUVIN
G. barbadense 249006
G. hirsutum JKC752
G. hirsutum JKC771
G. hirsutum JKC783
G. barbadense SUVIN(2)
G. hirsutum JKC703
G. herbaceum 221573
G. hirsutum JKC737
G. herbaceum RAHS14
G. hirsutum MCU5
G. hirsutum KC2
MP Tree
G. hirsutum JKC752
G. hirsutum JKC783
G. hirsutum KC2
G. herbaceum H17
G. hirsutum JKC725
G. herbaceum DB312
G. hirsutum JKC771
G. herbaceum RAHSIPS187
G. hirsutum JKC737
G. barbadense 249005
G. herbaceum 221557
G. barbadense249002
G. herbaceum JAYHELLAR
G. herbaceum 221547
G. hirsutum MCU5
G. barbadense 249006
G. herbaceum 221573
G. barbadense 249011
G. barbadense SUVIN
G. herbaceum AH7GP
G. barbadense 24009
G. hirsutum LRA5166
G. barbadense 249001
G. herbaceum RAHS14
G. arboreum 574
G. arboreum 577
G. arboreum 576
G. herbaceum AH41
G. arboreum AK235
G. hirsutum JKC777
G. barbadense 249007
G. barbadense 249008
G. arboreum DLSA17
G. barbadense 249012
G. arboreum 221568
G. herbaceum GC21
G. hirsutum JKC703
G. herbaceum VAGAD
G. arboreum 221567
G. arboreum 221566
G. hirsutum 770
G. hirsutum AS3
G. barbadense249010
G. herbaceum RAHS132
G. barbadense249004
G. arboreum 579
UPGMA Tree

(C)

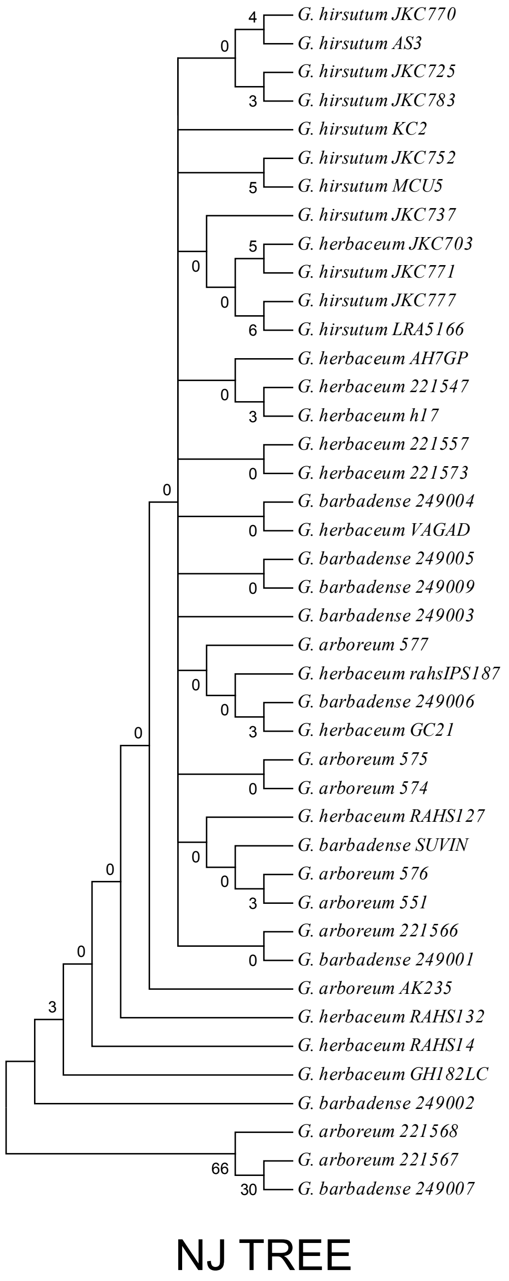
G. hirsutum JKC770
G. hirsutum AS3
G. hirsutum JKC725
G. hirsutum JKC783
G. hirsutum KC2
G. hirsutum JKC752
G. hirsutum MCU5
G. hirsutum JKC737
G. herbaceum JKC703
G. hirsutum JKC771
G. hirsutum JKC777
G. hirsutum LRA5166
G. herbaceum AH7GP
G. herbaceum 221547
G. herbaceum h17
G. herbaceum 221557
G. herbaceum 221573
G. barbadense 249004
G. herbaceum VAGAD
G. barbadense 249005
G. barbadense 249009
G. barbadense 249003
G. arboreum 577
G. herbaceum rahsIPS187
G. barbadense 249006
G. herbaceum GC21
G. arboreum 575
G. arboreum 574
G. herbaceum RAHS127
G. barbadense SUVIN
G. arboreum 576
G. arboreum 551
G. arboreum 221566
G. barbadense 249001
G. arboreum AK235
G. herbaceum RAHS132
G. herbaceum RAHS14
G. herbaceum GH182LC
G. barbadense 249002
G. arboreum 221568
G. arboreum 221567
G. barbadense 249007
NJ TREE

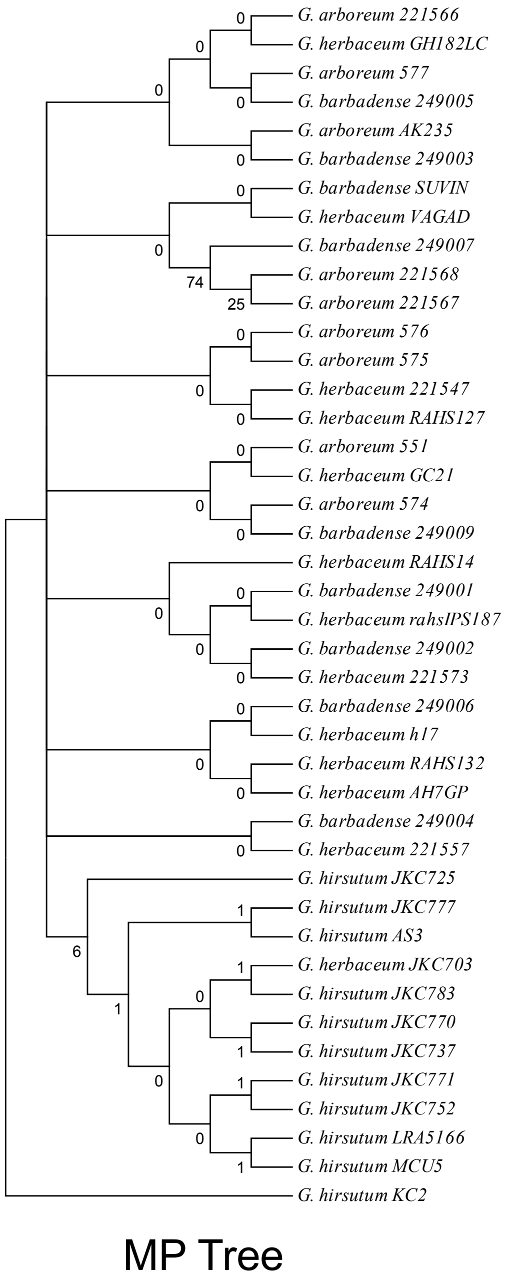
G. arboreum 221566
G. herbaceum GH182LC
G. arboreum 577
G. barbadense 249005
G. arboreum AK235
G. barbadense 249003
G. barbadense SUVIN
G. herbaceum VAGAD
G. barbadense 249007
G. arboreum 221568
G. arboreum 221567
G. arboreum 576
G. arboreum 575
G. herbaceum 221547
G. herbaceum RAHS127
G. arboreum 551
G. herbaceum GC21
G. arboreum 574
G. barbadense 249009
G. herbaceum RAHS14
G. barbadense 249001
G. herbaceum rahsIPS187
G. barbadense 249002
G. herbaceum 221573
G. barbadense 249006
G. herbaceum h17
G. herbaceum RAHS132
G. herbaceum AH7GP
G. barbadense 249004
G. herbaceum 221557
G. hirsutum JKC725
G. hirsutum JKC777
G. hirsutum AS3
G. herbaceum JKC703
G. hirsutum JKC783
G. hirsutum JKC770
G. hirsutum JKC737
G. hirsutum JKC771
G. hirsutum JKC752
G. hirsutum LRA5166
G. hirsutum MCU5
G. hirsutum KC2
MP Tree

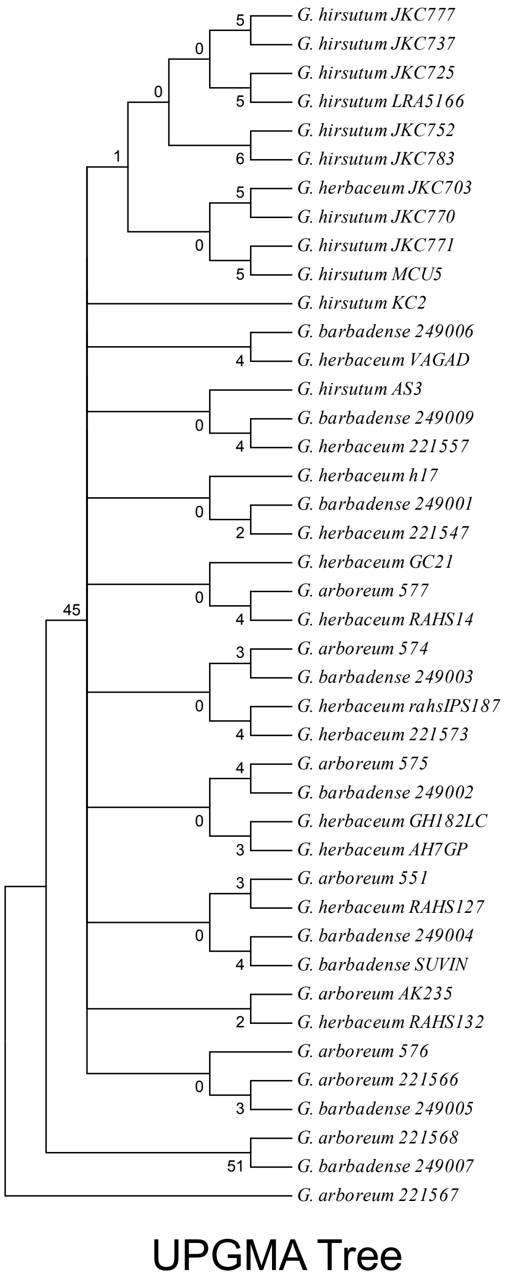
G. hirsutum JKC777
G. hirsutum JKC737
G. hirsutum JKC725
G. hirsutum LRA5166
G. hirsutum JKC752
G. hirsutum JKC783
G. herbaceum JKC703
G. hirsutum JKC770
G. hirsutum JKC771
G. hirsutum MCU5
G. hirsutum KC2
G. barbadense 249006
G. herbaceum VAGAD
G. hirsutum AS3
G. barbadense 249009
G. herbaceum 221557
G. herbaceum h17
G. barbadense 249001
G. herbaceum 221547
G. herbaceum GC21
G. arboreum 577
G. herbaceum RAHS14
G. arboreum 574
G. barbadense 249003
G. herbaceum rahsIPS187
G. herbaceum 221573
G. arboreum 575
G. barbadense 249002
G. herbaceum GH182LC
G. herbaceum AH7GP
G. arboreum 551
G. herbaceum RAHS127
G. barbadense 249004
G. barbadense SUVIN
G. arboreum AK235
G. herbaceum RAHS132
G. arboreum 576
G. arboreum 221566
G. barbadense 249005
G. arboreum 221568
G. barbadense 249007
G. arboreum 221567
UPGMA Tree

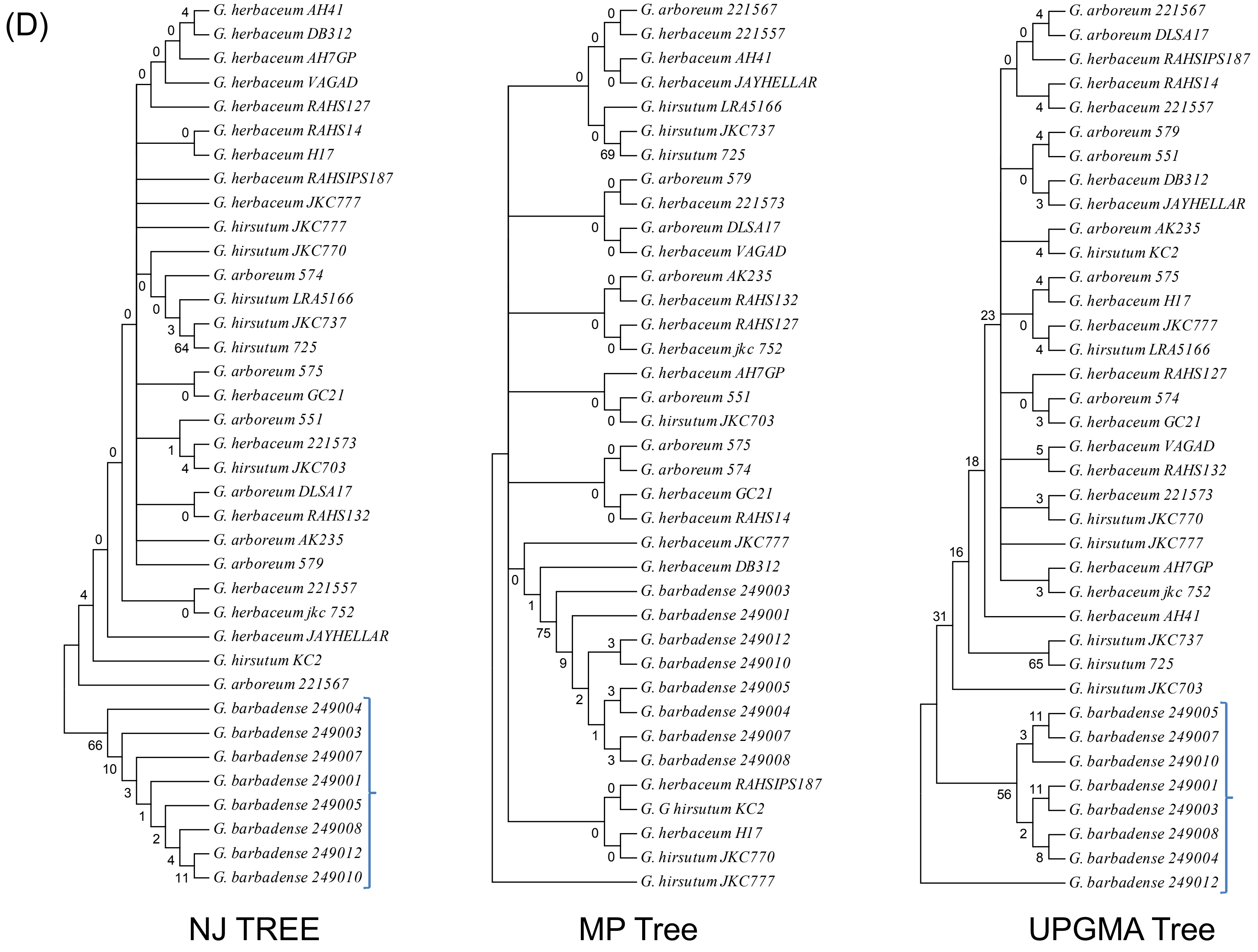
(D)
NJ TREE
MP Tree
UPGMA Tree
