# Supplementary material for: Universal Plant DNA Barcode Loci May Not Work in Complex Groups: A Case Study with Indian Berberis Species
Source: PLoS One. 2010 Oct 27;5(10):e13674. doi: 10.1371/journal.pone.0013674 (PMC2965122; doi:10.1371/journal.pone.0013674)
Supplement: Table S1 — The classification and distribution of Berberis species according toAhrendt(1961). The arrangement of species is alphabetical. The detailed GPS data for distribution of the selected species is given in Table S10. (0.02 MB PDF) [file pone.0013674.s007.pdf]

Table S1

S.No.	Species	Section	Subsection	Distribution
1	*B. angulosa*	*Angulosae*	*Euangulosae;*	Eastern Himalayas
2	*B. aristata*	*Tinctoriae*	*Chitrirae*	Western Himalayas
3	*B. asiatica*	*Asiaticae*	-	Western & Eastern Himalayas
4	*B. chitria*	*Tinctoriae*	*Chitrirae*	Western Himalayas
5	*B. glaucocarpa*	*Asiaticae*	-	Western Himalayas
6	*B. griffithiana*	*Wallichianae*	*Replicatae*	Eastern Himalayas
7	*B. hainesii*	*Asiaticae*	-	Hills of Central India
8	*B. insignis*	*Wallichianae*	*Insignis*	Eastern Himalayas
9	*B. jaescheana*	*Angulosae*	*Jaescheanae*	Western & Eastern Himalayas
10	*B. lycium*	*Asiaticae*	-	Western Himalayas
11	*B. macrosepala*	*Angulosae*	*Euangulosae*	Eastern Himalayas
12	*B. pachycantha*	*Vulgaris*	-	Western Himalayas
13	*B. replicata*	*Wallichianae*	*Replicatae*	Eastern Himalayas
14	*B. tinctoria*	*Tinctoriae*	*Eutinctoriae*	Nilgiri hills of Southern India
15	*B. umbellata*	*Tinctoriae*	*Umbellatae*	Western & Eastern Himalayas
16	*B. wightiana*	*Tinctoriae*	*Eutinctoriae*	Nilgiri hills of Southern India
