# Supplementary material for: Universal Plant DNA Barcode Loci May Not Work in Complex Groups: A Case Study with Indian Berberis Species
Source: PLoS One. 2010 Oct 27;5(10):e13674. doi: 10.1371/journal.pone.0013674 (PMC2965122; doi:10.1371/journal.pone.0013674)
Supplement: Table S2 — The classification of Ficus species according to Corner (1958). (0.05 MB PDF) [file pone.0013674.s008.pdf]

Table S2

S. No.	Species	Subgenus	Section
1	*F. benghalensis*	*Urostigma*	*Conosycea*
2	*F. benjamina*	*Urostigma*	*Conosycea*
3	*F. retusa*	*Urostigma*	*Conosycea*
4	*F. religiosa*	*Urostigma*	*Urostigma*
5	*F. rumphii*	*Urostigma*	*Urostigma*
6	*F. virens*	*Urostigma*	*Urostigma*
7	*F. elastica*	*Urostigma*	*Urostigma*
8	*F. trigona*	*Urostigma*	*Americana*
9	*F. racemosa*	*Sycomorus*	*Sycomorus*
10	*F. hispida*	*Sycomorus*	*Sycocarpus*
11	*F. carica*	*Ficus*	*Ficus*
