# Supplementary material for: Universal Plant DNA Barcode Loci May Not Work in Complex Groups: A Case Study with Indian Berberis Species
Source: PLoS One. 2010 Oct 27;5(10):e13674. doi: 10.1371/journal.pone.0013674 (PMC2965122; doi:10.1371/journal.pone.0013674)
Supplement: Table S3 — The binary matrix developed on the basis of detailed morphological parameters considered for species delineation in Berberis. The character matrix developed on the basis of detailed morphological parameters considered for species delineation in Berberis. (0.09 MB PDF) [file pone.0013674.s009.pdf]

Table S3

		stem				spines		leaves									inflorescence						fruit	
**Species**	**DNA No.**	**texture**	**shape**	**color**	**branching habit**	**number**	**size**	**nature**	**color**	**texture**	**length to width ratio**	**density**	**base**	**margin**	**apex**	**petiole**	**nature**	**number**	**petal size**	**stamen**	**ovules**	**style**	**color**	**texture**
Berberis angulosa	B121	2	2	2	2	2	2	1	2	2	1	2	4	1	1	0	1	1	2	1	2	0	1	1
Berberis angulosa	B122	2	2	2	2	2	2	1	2	2	1	2	4	1	1	0	1	1	2	1	2	0	1	1
Berberis angulosa	B124	2	2	2	2	2	2	1	2	2	1	2	4	1	1	0	1	1	2	1	2	0	1	1
Berberis aristata	B55	1	2	1	3	2	2	1	1	2	2	1	5	2	4	1	4	2	2	2	2	2	1	1
Berberis aristata	B57	1	2	1	3	2	1	1	1	2	2	1	5	2	4	1	4	2	2	2	2	2	1	1
Berberis asiatica	B45	1	2	1	3	2	2	1	1	1	1	3	4	2	4	0	4	2	2	1	1	2	2	1
Berberis asiatica	B28	1	1	1	3	2	2	1	1	1	1	3	4	2	4	0	4	2	2	1	1	2	2	1
Berberis asiatica	B27	1	1	1	3	2	2	1	1	1	1	3	4	2	4	0	4	2	2	1	1	2	2	1
Berberis asiatica	B50	1	1	1	3	2	2	1	1	1	1	3	4	2	4	0	4	2	2	1	1	2	2	1
Berberis asiatica	B42	1	1	1	3	2	2	1	1	1	1	3	4	2	4	0	4	2	2	1	1	2	2	1
Berberis chitria	B175	2	2	2	1	2	2	1	1	2	1	2	4	2	4	0	5	2	2	2	2	2	1	2
Berberis chitria	B20	2	1	2	1	2	2	1	1	2	1	2	4	2	4	0	4	2	2	2	2	2	1	2
Berberis chitria	B14	2	2	2	1	2	2	1	1	2	1	2	4	2	4	0	5	2	2	2	2	2	1	2
Berberis chitria	B33	2	1	2	1	2	2	1	1	2	1	2	4	2	1	0	5	2	2	2	2	2	1	2
Berberis chitria	B34	2	2	2	1	2	2	1	1	2	1	2	4	2	4	0	4	2	2	2	2	2	1	2
Berberis glaucocarpa	B110	1	1	1	2	1	1	2	2	2	1	2	4	1	2	0	4	2	3	2	2	2	2	1
Berberis glaucocarpa	B170	1	1	1	2	1	2	2	2	2	2	2	4	3	2	0	4	2	3	2	2	2	2	1
Berberis glaucocarpa	B111	1	1	1	2	1	1	2	2	2	1	2	4	1	2	0	4	2	3	2	2	2	2	1
Berberis glaucocarpa	B171	1	1	1	2	1	1	2	2	2	1	2	4	1	2	0	4	2	3	2	2	2	2	1
Berberis glaucocarpa	B168	1	1	1	2	1	1	2	2	2	1	2	4	1	2	0	4	2	3	2	2	1	2	1
Berberis glaucocarpa	B109	1	1	1	2	1	1	2	2	2	1	2	4	1	2	0	4	2	3	2	2	2	2	1
Berberis glaucocarpa	B107	1	1	1	2	1	1	2	2	2	2	2	4	1	2	0	4	2	3	2	2	2	2	1
Berberis hainesii	B151	1	1	1	1	2	1	1	2	2	1	1	6	2	4	1	3	1	1	2	1	0	2	1
Berberis hainesii	B153	1	1	1	1	2	2	1	2	2	1	1	6	2	4	1	3	1	1	2	1	0	2	1
Berberis hainesii	B157	1	1	1	1	2	2	1	2	2	1	1	6	2	4	1	3	1	1	2	1	0	2	1
Berberis hainesii	B152	1	1	1	1	2	2	1	2	2	1	1	6	2	4	1	3	1	1	2	1	0	2	1
Berberis jaeschkeana	B176	2	2	1	1	2	1	2	1	2	1	3	3	2	4	0	3	1	2	1	2	1	1	2
Berberis jaeschkeana	B178	2	2	1	1	2	1	2	1	2	2	3	3	2	4	0	3	1	2	1	2	1	1	2
Berberis jaeschkeana	B182	2	2	1	1	2	1	2	1	2	1	3	3	2	4	0	1	1	2	1	2	1	1	2
Berberis jaeschkeana	B183	2	2	1	1	2	2	2	1	2	1	3	3	2	4	0	1	1	2	1	2	1	1	2
Berberis jaeschkeana	B185	2	2	1	1	2	1	2	1	2	2	3	3	2	4	0	1	1	2	1	2	1	1	2
Berberis lycium	B1	1	2	1	3	2	2	2	1	2	2	2	1	1	1	0	4	2	2	2	2	1	2	1
Berberis lycium	B2	1	2	1	3	2	2	2	1	2	2	2	1	1	1	0	4	2	2	2	2	1	2	1
Berberis lycium	B3	1	2	1	3	2	2	2	1	2	2	2	1	1	1	0	4	2	2	2	2	1	2	1
Berberis lycium	B4	1	2	1	3	2	2	2	1	2	2	2	1	1	1	0	4	2	2	2	2	1	2	1
Berberis lycium	B5	1	2	1	3	2	2	2	1	2	2	2	1	1	1	0	4	2	2	2	2	1	2	1
Berberis lycium	B6	1	2	1	3	2	2	2	1	2	2	2	1	1	1	0	4	2	2	2	2	1	2	1
Berberis pachyacantha	B181	1	2	2	1	2	1	2	2	2	2	2	2	2	1	1	4	2	2	1	1	0	1	2
Berberis pachyacantha	B180	1	2	2	1	2	1	2	2	2	2	2	2	2	1	1	4	2	2	1	1	0	1	2
Berberis pachyacantha	B179	1	2	2	1	2	1	2	2	2	1	2	2	2	1	1	4	2	2	1	1	0	1	2
Berberis pachyacantha	B184	1	2	2	1	2	1	2	2	2	2	2	2	2	3	1	4	2	2	1	1	0	1	2
Berberis replicata	B114	1	1	1	1	2	2	1	1	1	2	3	3	2	4	0	3	1	2	2	1	1	2	1
Berberis replicata	B149	1	1	1	1	2	2	1	1	1	2	3	3	2	4	0	3	2	2	2	1	1	2	1
Berberis replicata	B147	1	1	1	1	2	2	1	1	1	2	3	3	2	4	0	3	1	2	2	1	1	2	1
Berberis replicata	B116	1	1	1	1	2	2	1	1	1	2	3	3	2	4	0	3	1	2	2	1	1	2	1
Berberis tinctoria	B103	1	2	1	1	2	2	1	1	1	2	1	4	1	3	1	4	2	2	2	1	1	2	1
Berberis tinctoria	B92	1	2	1	1	2	2	1	1	1	2	1	4	1	3	1	4	2	2	2	1	1	2	1
Berberis tinctoria	B87	1	2	1	1	2	2	1	1	1	2	1	4	1	3	1	4	2	2	2	1	1	2	1
Berberis tinctoria	B89	1	2	1	1	2	2	1	1	1	2	1	4	1	3	1	4	2	2	2	1	1	2	1
Berberis tinctoria	B100	1	2	1	1	2	2	1	1	1	2	1	4	1	3	1	4	2	2	2	1	1	2	1
Berberis tinctoria	B88	1	2	1	1	2	2	1	1	1	2	1	4	1	3	1	4	2	2	2	1	1	2	1
Berberis tinctoria	B90	1	2	1	1	2	2	1	1	1	2	1	4	1	3	1	4	2	2	2	1	1	2	1
Berberis umbellata	B160	1	2	2	2	2	2	1	2	2	1	3	4	2	1	0	3	1	3	1	2	1	1	2
Berberis umbellata	B158	1	2	2	2	2	2	1	2	2	1	3	4	2	1	0	3	1	3	1	2	1	1	2
Berberis umbellata	B167	1	2	2	2	2	2	1	2	2	1	3	4	2	1	0	3	1	3	1	2	1	1	2
Berberis wightiana	B93	2	2	1	1	2	2	1	1	2	2	1	5	1	1	1	4	2	2	2	1	2	2	1
Berberis wightiana	B95	2	2	1	1	2	2	1	1	2	2	1	5	1	1	1	4	2	2	2	1	2	2	1
Berberis wightiana	B94	2	2	1	1	2	2	1	1	2	1	1	5	1	1	1	4	2	2	2	1	2	2	1

Coding scheme for the above character matrix

plant part	character	character state	code
stem	texture	glabrous	1
		puberulous	2
	shape	terete or subterete	1
		angled or sulcate	2
	color	yellow	1
		dark red	2
	branching habit	sparse	1
		intermediate	2
		dense	3
spines	number	solitary	1
		3-fid	2
	size	upto 9mm	1
		> 9mm	2
leaves	nature	persistent	1
		deciduous	2
	color	pale green	1
		deep green	2
	texture	thick rigid with hypoderm	1
		thin flexible without hypoderm	2
	length width ratio	upto 2	1
		>2	2
	density	low	1
		medium	2
		high	3
	base	auenuate	1
		contracted	2
		tapering	3
		cuneate	4
		rounded	5
		truncate	6
	margin	entire	1
		spinulose	2
	apex	mucronate	1
		acute	2
		rounded	3
		spine tip	4
	petiole	absent	0
		present	1
inflorescence	nature	fascicled	1
		umbellate	2
		pseudoumbellate	3
		receme	4
		paniculate	5
	number	upto 6	1
		>6	2
	petal size	equal to inner sepals	1
		shorter than inner sepals	2
		larger than inner sepals	3
	stamen	truncate	1
		produced	2
	ovules	upto3	1
		>3	2
	style	absent	0
		upto 0.5 mm	1
		>0.5mm	2
fruit	color	red or reddish purple	1
		black	2
	texture	pruinose	1
		epruinose	2
