# Supplementary material for: Universal Plant DNA Barcode Loci May Not Work in Complex Groups: A Case Study with Indian Berberis Species
Source: PLoS One. 2010 Oct 27;5(10):e13674. doi: 10.1371/journal.pone.0013674 (PMC2965122; doi:10.1371/journal.pone.0013674)
Supplement: Table S4 — One Way ANOVA with Bonferroni's multiple comparison tests to compare inter (A) and intraspecific (B) variability for each individual locus in Berberis. (0.06 MB PDF) [file pone.0013674.s010.pdf]

Table S4

A

Overall p<0.0001					
Bartlett's test for equal variances p=0.07					
Bonferroni's Multiple Comparison Test	Mean Diff.	t	P value	95% CI of diff	Result
ITS vs *matK*	0.3589	6.578	P < 0.001	0.2095 to 0.5084	ITS>>*matK*
ITS vs *rbcL*	0.9834	18.72	P < 0.001	0.8395 to 1.127	ITS>>*rbcL*
ITS vs *trnH-psbA*	0.08987	1.68	P > 0.05	-0.05662 to 0.2364	ITS=*trnH-psbA*
matK vs *rbcL*	0.6244	11.28	P < 0.001	0.4727 to 0.7761	*matK>>rbcL*
matK vs *trnH-psbA*	-0.2691	4.78	P < 0.001	-0.4232 to -0.1149	*trnH-psbA>>matK*
rbcL vs *trnH-psbA*	-0.8935	16.45	P < 0.001	-1.042 to -0.7447	*trnH-psbA>>rbcL*

B

Overall p=0.0044					
Bartlett's test for equal variances p=0.12					
Bonferroni's Multiple Comparison Test	Mean Diff.	t	P value	95% CI of diff	Result
ITS vs *matK*	0.4098	1.649	P > 0.05	-0.2718 to 1.091	ITS=*matK*
ITS vs *rbcL*	0.9443	3.801	P < 0.01	0.2628 to 1.626	*rbcL*>>ITS
ITS vs *trnH-psbA*	0.5338	2.192	P > 0.05	-0.1342 to 1.202	ITS=*trnH-psbA*
matK vs *rbcL*	0.5346	2.048	P > 0.05	-0.1814 to 1.250	*matK=rbcL*
matK vs *trnH-psbA*	0.124	0.484	P > 0.05	-0.5790 to 0.8270	*trnH-psbA=matK*
rbcL vs *trnH-psbA*	-0.4106	1.602	P > 0.05	-1.114 to 0.2925	*trnH-psbA=rbcL*
