# Supplementary material for: Universal Plant DNA Barcode Loci May Not Work in Complex Groups: A Case Study with Indian Berberis Species
Source: PLoS One. 2010 Oct 27;5(10):e13674. doi: 10.1371/journal.pone.0013674 (PMC2965122; doi:10.1371/journal.pone.0013674)
Supplement: Table S5 — One way ANOVA with Bonferroni's multiple comparison tests to compare inter (A) and intraspecific (B) variability for each possible multilocus combinations in Berberis. (0.06 MB PDF) [file pone.0013674.s011.pdf]

Table S5

A

Overall p<0.0001					
Bartlett's test for equal variances p=0.6005					
Bonferroni's Multiple Comparison Test	Mean Diff.	t	P value	95% CI of diff	Result
ITS+*matK* vs ITS+*matK*+*rbcL*	0.001205	1.627	P > 0.05	-0.001309 to 0.003719	ITS+*matK*=ITS+*matK*+*rbcL*
ITS+*matK* vs ITS+*rbcL*	0.0004231	0.5713	P > 0.05	-0.002091 to 0.002937	ITS+*matK*=ITS+*rbcL*
ITS+*matK* vs ITS+trnH	-0.002179	2.943	P > 0.05	-0.004693 to 0.0003343	ITS+*matK*=ITS+*trnH-psbA*
ITS+*matK* vs ITS+trnH+*matK*	-0.0002564	0.3462	P > 0.05	-0.002770 to 0.002257	ITS+*matK*=ITS+*trnH-psbA*+*matK*
ITS+*matK* vs ITS+trnH+*matK*+*rbcL*	0.0006667	0.9002	P > 0.05	-0.001847 to 0.003180	ITS+*matK*=ITS+*trnH-psbA*+*matK*+*rbcL*
ITS+*matK* vs ITS+trnH+*rbcL*	-0.0001154	0.1558	P > 0.05	-0.002629 to 0.002398	ITS+*matK*=ITS+*trnH-psbA*+*rbcL*
ITS+*matK* vs *matK*+*rbcL*	0.00309	4.172	P < 0.01	0.0005760 to 0.005603	ITS+*matK*>*matK*+*rbcL*
ITS+*matK* vs trnH+*matK*	0.001192	1.61	P > 0.05	-0.001321 to 0.003706	ITS+*matK*=*trnH-psbA*+*matK*
ITS+*matK* vs trnH+*matK*+*rbcL*	0.002038	2.752	P > 0.05	-0.0004753 to 0.004552	ITS+*matK*=*trnH-psbA*+*matK*+*rbcL*
ITS+*matK* vs trnH+*rbcL*	0.001654	2.233	P > 0.05	-0.0008599 to 0.004168	ITS+*matK*=*trnH-psbA*+*rbcL*
ITS+*matK*+*rbcL* vs ITS+*rbcL*	-0.0007821	1.056	P > 0.05	-0.003296 to 0.001732	ITS+*matK*+*rbcL*=ITS+*rbcL*
ITS+*matK*+*rbcL* vs ITS+trnH	-0.003385	4.57	P < 0.001	-0.005898 to -0.0008709	ITS+*trnH-psbA*>>ITS+*matK*+*rbcL*
ITS+*matK*+*rbcL* vs ITS+trnH+*matK*	-0.001462	1.973	P > 0.05	-0.003975 to 0.001052	ITS+*matK*+*rbcL*=ITS+*trnH-psbA*+*matK*
ITS+*matK*+*rbcL* vs ITS+trnH+*matK*+*rbcL*	-0.0005385	0.7271	P > 0.05	-0.003052 to 0.001975	ITS+*matK*+*rbcL*=ITS+*trnH-psbA*+*matK*+*rbcL*
ITS+*matK*+*rbcL* vs ITS+trnH+*rbcL*	-0.001321	1.783	P > 0.05	-0.003834 to 0.001193	ITS+*matK*+*rbcL*=ITS+*trnH-psbA*+*rbcL*
ITS+*matK*+*rbcL* vs *matK*+*rbcL*	0.001885	2.545	P > 0.05	-0.0006291 to 0.004398	ITS+*matK*+*rbcL*=*matK*+*rbcL*
ITS+*matK*+*rbcL* vs trnH+*matK*	-0.00001282	0.01731	P > 0.05	-0.002527 to 0.002501	ITS+*matK*+*rbcL*=*trnH-psbA*+*matK*
ITS+*matK*+*rbcL* vs trnH+*matK*+*rbcL*	0.0008333	1.125	P > 0.05	-0.001680 to 0.003347	ITS+*matK*+*rbcL*=*trnH-psbA*+*matK*+*rbcL*
ITS+*matK*+*rbcL* vs trnH+*rbcL*	0.0004487	0.6059	P > 0.05	-0.002065 to 0.002962	ITS+*matK*+*rbcL*=*trnH-psbA*+*rbcL*
ITS+*rbcL* vs ITS+trnH	-0.002603	3.514	P < 0.05	-0.005116 to -0.00008882	ITS+*trnH-psbA*>ITS+*rbcL*
ITS+*rbcL* vs ITS+trnH+*matK*	-0.0006795	0.9175	P > 0.05	-0.003193 to 0.001834	ITS+*rbcL*=ITS+*trnH-psbA*+*matK*
ITS+*rbcL* vs ITS+trnH+*matK*+*rbcL*	0.0002436	0.3289	P > 0.05	-0.002270 to 0.002757	ITS+*rbcL*=ITS+*trnH-psbA*+*matK*+*rbcL*
ITS+*rbcL* vs ITS+trnH+*rbcL*	-0.0005385	0.7271	P > 0.05	-0.003052 to 0.001975	ITS+*rbcL*=ITS+*trnH-psbA*+*rbcL*
ITS+*rbcL* vs *matK*+*rbcL*	0.002667	3.601	P < 0.05	0.0001529 to 0.005180	ITS+*rbcL*>*matK*+*rbcL*
ITS+*rbcL* vs trnH+*matK*	0.0007692	1.039	P > 0.05	-0.001745 to 0.003283	ITS+*rbcL*=*trnH-psbA*+*matK*
ITS+*rbcL* vs trnH+*matK*+*rbcL*	0.001615	2.181	P > 0.05	-0.0008984 to 0.004129	ITS+*rbcL*=*trnH-psbA*+*matK*+*rbcL*
ITS+*rbcL* vs trnH+*rbcL*	0.001231	1.662	P > 0.05	-0.001283 to 0.003745	ITS+*rbcL*=*trnH-psbA*+*rbcL*
ITS+trnH vs ITS+trnH+*matK*	0.001923	2.597	P > 0.05	-0.0005907 to 0.004437	ITS+*trnH-psbA*=ITS+*trnH-psbA*+*matK*
ITS+trnH vs ITS+trnH+*matK*+*rbcL*	0.002846	3.843	P < 0.05	0.0003324 to 0.005360	ITS+*trnH-psbA*>ITS+*trnH-psbA*+*matK*+*rbcL*
ITS+trnH vs ITS+trnH+*rbcL*	0.002064	2.787	P > 0.05	-0.0004496 to 0.004578	ITS+*trnH-psbA*=ITS+*trnH-psbA*+*rbcL*
ITS+trnH vs *matK*+*rbcL*	0.005269	7.115	P < 0.001	0.002755 to 0.007783	ITS+*trnH-psbA*>>*matK*+*rbcL*
ITS+trnH vs trnH+*matK*	0.003372	4.553	P < 0.001	0.0008581 to 0.005886	ITS+*trnH-psbA*>>*trnH-psbA*+*matK*
ITS+trnH vs trnH+*matK*+*rbcL*	0.004218	5.695	P < 0.001	0.001704 to 0.006732	ITS+*trnH-psbA*>>*trnH-psbA*+*matK*+*rbcL*
ITS+trnH vs trnH+*rbcL*	0.003833	5.176	P < 0.001	0.001320 to 0.006347	ITS+*trnH-psbA*>>*trnH-psbA*+*rbcL*
ITS+trnH+*matK* vs ITS+trnH+*matK*+*rbcL*	0.0009231	1.246	P > 0.05	-0.001591 to 0.003437	ITS+*trnH-psbA*+*matK*=ITS+*trnH-psbA*+*matK*+*rbcL*
ITS+trnH+*matK* vs ITS+trnH+*rbcL*	0.000141	0.1904	P > 0.05	-0.002373 to 0.002655	ITS+*trnH-psbA*+*matK*=ITS+*trnH-psbA*+*rbcL*
ITS+trnH+*matK* vs *matK*+*rbcL*	0.003346	4.518	P < 0.001	0.0008324 to 0.005860	ITS+*trnH-psbA*+*matK*>>*matK*+*rbcL*
ITS+trnH+*matK* vs trnH+*matK*	0.001449	1.956	P > 0.05	-0.001065 to 0.003962	ITS+*trnH-psbA*+*matK*=*trnH-psbA*+*matK*
ITS+trnH+*matK* vs trnH+*matK*+*rbcL*	0.002295	3.099	P > 0.05	-0.0002189 to 0.004809	ITS+*trnH-psbA*+*matK*=*trnH-psbA*+*matK*+*rbcL*
ITS+trnH+*matK* vs trnH+*rbcL*	0.00191	2.579	P > 0.05	-0.0006035 to 0.004424	ITS+*trnH-psbA*+*matK*=*trnH-psbA*+*rbcL*
ITS+trnH+*matK*+*rbcL* vs ITS+trnH+*rbcL*	-0.0007821	1.056	P > 0.05	-0.003296 to 0.001732	ITS+*trnH-psbA*+*matK*+*rbcL*=ITS+*trnH-psbA*+*rbcL*
ITS+trnH+*matK*+*rbcL* vs *matK*+*rbcL*	0.002423	3.272	P > 0.05	-0.00009067 to 0.004937	ITS+*trnH-psbA*+*matK*+*rbcL*=*matK*+*rbcL*
ITS+trnH+*matK*+*rbcL* vs trnH+*matK*	0.0005256	0.7098	P > 0.05	-0.001988 to 0.003039	ITS+*trnH-psbA*+*matK*+*rbcL*=*trnH-psbA*+*matK*
ITS+trnH+*matK*+*rbcL* vs trnH+*matK*+*rbcL*	0.001372	1.852	P > 0.05	-0.001142 to 0.003886	ITS+*trnH-psbA*+*matK*+*rbcL*=*trnH-psbA*+*matK*+*rbcL*
ITS+trnH+*matK*+*rbcL* vs trnH+*rbcL*	0.0009872	1.333	P > 0.05	-0.001527 to 0.003501	ITS+*trnH-psbA*+*matK*+*rbcL*=*trnH-psbA*+*rbcL*
ITS+trnH+*rbcL* vs *matK*+*rbcL*	0.003205	4.328	P < 0.01	0.0006914 to 0.005719	ITS+*trnH-psbA*+*rbcL*>*matK*+*rbcL*
ITS+trnH+*rbcL* vs trnH+*matK*	0.001308	1.766	P > 0.05	-0.001206 to 0.003821	ITS+*trnH-psbA*+*rbcL*=*trnH-psbA*+*matK*
ITS+trnH+*rbcL* vs trnH+*matK*+*rbcL*	0.002154	2.908	P > 0.05	-0.0003599 to 0.004668	ITS+*trnH-psbA*+*rbcL*=*trnH-psbA*+*matK*+*rbcL*
ITS+trnH+*rbcL* vs trnH+*rbcL*	0.001769	2.389	P > 0.05	-0.0007445 to 0.004283	ITS+*trnH-psbA*+*rbcL*=*trnH-psbA*+*rbcL*
matK+*rbcL* vs trnH+*matK*	-0.001897	2.562	P > 0.05	-0.004411 to 0.0006163	*matK*+*rbcL*=*trnH-psbA*+*matK*
matK+*rbcL* vs trnH+*matK*+*rbcL*	-0.001051	1.42	P > 0.05	-0.003565 to 0.001462	*matK*+*rbcL*=*trnH-psbA*+*matK*+*rbcL*
matK+*rbcL* vs trnH+*rbcL*	-0.001436	1.939	P > 0.05	-0.003950 to 0.001078	*matK*+*rbcL*=*trnH-psbA*+*rbcL*
trnH+*matK* vs trnH+*matK*+*rbcL*	0.0008462	1.143	P > 0.05	-0.001668 to 0.003360	*trnH-psbA*+*matK*=*trnH-psbA*+*matK*+*rbcL*
trnH+*matK* vs trnH+*rbcL*	0.0004615	0.6232	P > 0.05	-0.002052 to 0.002975	*trnH-psbA*+*matK*=*trnH-psbA*+*rbcL*
trnH+*matK*+*rbcL* vs trnH+*rbcL*	-0.0003846	0.5193	P > 0.05	-0.002898 to 0.002129	*trnH-psbA*+*matK*+*rbcL*=*trnH-psbA*+*rbcL*

B

Overall p=0.9998					
Bartlett's test for equal variances p=0.08					
Bonferroni's Multiple Comparison Test	Mean Diff.	t	P value	95% CI of diff	Result
ITS+*matK* vs ITS+*matK*+*rbcL*	0.00025	0.3678	P > 0.05	-0.002062 to 0.002562	ITS+*matK*=ITS+*matK*+*rbcL*
ITS+*matK* vs ITS+*rbcL*	0	0	P > 0.05	-0.002312 to 0.002312	ITS+*matK*=ITS+*rbcL*
ITS+*matK* vs ITS+trnH	-0.00008333	0.1226	P > 0.05	-0.002396 to 0.002229	ITS+*matK*=ITS+*trnH-psbA*
ITS+*matK* vs ITS+trnH+*matK*	0.0001667	0.2452	P > 0.05	-0.002146 to 0.002479	ITS+*matK*=ITS+*trnH-psbA*+*matK*
ITS+*matK* vs ITS+trnH+*matK*+*rbcL*	0.00025	0.3678	P > 0.05	-0.002062 to 0.002562	ITS+*matK*=ITS+*trnH-psbA*+*matK*+*rbcL*
ITS+*matK* vs ITS+trnH+*rbcL*	0	0	P > 0.05	-0.002312 to 0.002312	ITS+*matK*=ITS+*trnH-psbA*+*rbcL*
ITS+*matK* vs *matK*+*rbcL*	0.0003333	0.4903	P > 0.05	-0.001979 to 0.002646	ITS+*matK*=*matK*+*rbcL*
ITS+*matK* vs trnH+*matK*	0.00025	0.3678	P > 0.05	-0.002062 to 0.002562	ITS+*matK*=*trnH-psbA*+*matK*
ITS+*matK* vs trnH+*matK*+*rbcL*	0.00025	0.3678	P > 0.05	-0.002062 to 0.002562	ITS+*matK*=*trnH-psbA*+*matK*+*rbcL*
ITS+*matK* vs trnH+*rbcL*	0.0003333	0.4903	P > 0.05	-0.001979 to 0.002646	ITS+*matK*=*trnH-psbA*+*rbcL*
ITS+*matK*+*rbcL* vs ITS+*rbcL*	-0.00025	0.3678	P > 0.05	-0.002562 to 0.002062	ITS+*matK*+*rbcL*=ITS+*rbcL*
ITS+*matK*+*rbcL* vs ITS+trnH	-0.0003333	0.4903	P > 0.05	-0.002646 to 0.001979	ITS+*matK*+*rbcL*=ITS+*trnH-psbA*
ITS+*matK*+*rbcL* vs ITS+trnH+*matK*	-0.00008333	0.1226	P > 0.05	-0.002396 to 0.002229	ITS+*matK*+*rbcL*=ITS+*trnH-psbA*+*matK*
ITS+*matK*+*rbcL* vs ITS+trnH+*matK*+*rbcL*	0	0	P > 0.05	-0.002312 to 0.002312	ITS+*matK*+*rbcL*=ITS+*trnH-psbA*+*matK*+*rbcL*
ITS+*matK*+*rbcL* vs ITS+trnH+*rbcL*	-0.00025	0.3678	P > 0.05	-0.002562 to 0.002062	ITS+*matK*+*rbcL*=ITS+*trnH-psbA*+*rbcL*
ITS+*matK*+*rbcL* vs *matK*+*rbcL*	0.00008333	0.1226	P > 0.05	-0.002229 to 0.002396	ITS+*matK*+*rbcL*=*matK*+*rbcL*
ITS+*matK*+*rbcL* vs trnH+*matK*	0	0	P > 0.05	-0.002312 to 0.002312	ITS+*matK*+*rbcL*=*trnH-psbA*+*matK*
ITS+*matK*+*rbcL* vs trnH+*matK*+*rbcL*	0	0	P > 0.05	-0.002312 to 0.002312	ITS+*matK*+*rbcL*=*trnH-psbA*+*matK*+*rbcL*
ITS+*matK*+*rbcL* vs trnH+*rbcL*	0.00008333	0.1226	P > 0.05	-0.002229 to 0.002396	ITS+*matK*+*rbcL*=*trnH-psbA*+*rbcL*
ITS+*rbcL* vs ITS+trnH	-0.00008333	0.1226	P > 0.05	-0.002396 to 0.002229	ITS+*rbcL*=ITS+*trnH-psbA*
ITS+*rbcL* vs ITS+trnH+*matK*	0.0001667	0.2452	P > 0.05	-0.002146 to 0.002479	ITS+*rbcL*=ITS+*trnH-psbA*+*matK*
ITS+*rbcL* vs ITS+trnH+*matK*+*rbcL*	0.00025	0.3678	P > 0.05	-0.002062 to 0.002562	ITS+*rbcL*=ITS+*trnH-psbA*+*matK*+*rbcL*
ITS+*rbcL* vs ITS+trnH+*rbcL*	0	0	P > 0.05	-0.002312 to 0.002312	ITS+*rbcL*=ITS+*trnH-psbA*+*rbcL*
ITS+*rbcL* vs *matK*+*rbcL*	0.0003333	0.4903	P > 0.05	-0.001979 to 0.002646	ITS+*rbcL*=*matK*+*rbcL*
ITS+*rbcL* vs trnH+*matK*	0.00025	0.3678	P > 0.05	-0.002062 to 0.002562	ITS+*rbcL*=*trnH-psbA*+*matK*
ITS+*rbcL* vs trnH+*matK*+*rbcL*	0.00025	0.3678	P > 0.05	-0.002062 to 0.002562	ITS+*rbcL*=*trnH-psbA*+*matK*+*rbcL*
ITS+*rbcL* vs trnH+*rbcL*	0.0003333	0.4903	P > 0.05	-0.001979 to 0.002646	ITS+*rbcL*=*trnH-psbA*+*rbcL*
ITS+trnH vs ITS+trnH+*matK*	0.00025	0.3678	P > 0.05	-0.002062 to 0.002562	ITS+*trnH-psbA*=ITS+*trnH-psbA*+*matK*
ITS+trnH vs ITS+trnH+*matK*+*rbcL*	0.0003333	0.4903	P > 0.05	-0.001979 to 0.002646	ITS+*trnH-psbA*=ITS+*trnH-psbA*+*matK*+*rbcL*
ITS+trnH vs ITS+trnH+*rbcL*	0.00008333	0.1226	P > 0.05	-0.002229 to 0.002396	ITS+*trnH-psbA*=ITS+*trnH-psbA*+*rbcL*
ITS+trnH vs *matK*+*rbcL*	0.0004167	0.6129	P > 0.05	-0.001896 to 0.002729	ITS+*trnH-psbA*=*matK*+*rbcL*
ITS+trnH vs trnH+*matK*	0.0003333	0.4903	P > 0.05	-0.001979 to 0.002646	ITS+*trnH-psbA*=*trnH-psbA*+*matK*
ITS+trnH vs trnH+*matK*+*rbcL*	0.0003333	0.4903	P > 0.05	-0.001979 to 0.002646	ITS+*trnH-psbA*=*trnH-psbA*+*matK*+*rbcL*
ITS+trnH vs trnH+*rbcL*	0.0004167	0.6129	P > 0.05	-0.001896 to 0.002729	ITS+*trnH-psbA*=*trnH-psbA*+*rbcL*
ITS+trnH+*matK* vs ITS+trnH+*matK*+*rbcL*	0.00008333	0.1226	P > 0.05	-0.002229 to 0.002396	ITS+*trnH-psbA*+*matK*=ITS+*trnH-psbA*+*matK*+*rbcL*
ITS+trnH+*matK* vs ITS+trnH+*rbcL*	-0.0001667	0.2452	P > 0.05	-0.002479 to 0.002146	ITS+*trnH-psbA*+*matK*=ITS+*trnH-psbA*+*rbcL*
ITS+trnH+*matK* vs *matK*+*rbcL*	0.0001667	0.2452	P > 0.05	-0.002146 to 0.002479	ITS+*trnH-psbA*+*matK*=*matK*+*rbcL*
ITS+trnH+*matK* vs trnH+*matK*	0.00008333	0.1226	P > 0.05	-0.002229 to 0.002396	ITS+*trnH-psbA*+*matK*=*trnH-psbA*+*matK*
ITS+trnH+*matK* vs trnH+*matK*+*rbcL*	0.00008333	0.1226	P > 0.05	-0.002229 to 0.002396	ITS+*trnH-psbA*+*matK*=*trnH-psbA*+*matK*+*rbcL*
ITS+trnH+*matK* vs trnH+*rbcL*	0.0001667	0.2452	P > 0.05	-0.002146 to 0.002479	ITS+*trnH-psbA*+*matK*=*trnH-psbA*+*rbcL*
ITS+trnH+*matK*+*rbcL* vs ITS+trnH+*rbcL*	-0.00025	0.3678	P > 0.05	-0.002562 to 0.002062	ITS+*trnH-psbA*+*matK*+*rbcL*=ITS+*trnH-psbA*+*rbcL*
ITS+trnH+*matK*+*rbcL* vs *matK*+*rbcL*	0.00008333	0.1226	P > 0.05	-0.002229 to 0.002396	ITS+*trnH-psbA*+*matK*+*rbcL*=*matK*+*rbcL*
ITS+trnH+*matK*+*rbcL* vs trnH+*matK*	0	0	P > 0.05	-0.002312 to 0.002312	ITS+*trnH-psbA*+*matK*+*rbcL*=*trnH-psbA*+*matK*
ITS+trnH+*matK*+*rbcL* vs trnH+*matK*+*rbcL*	0	0	P > 0.05	-0.002312 to 0.002312	ITS+*trnH-psbA*+*matK*+*rbcL*=*trnH-psbA*+*matK*+*rbcL*
ITS+trnH+*matK*+*rbcL* vs trnH+*rbcL*	0.00008333	0.1226	P > 0.05	-0.002229 to 0.002396	ITS+*trnH-psbA*+*matK*+*rbcL*=*trnH-psbA*+*rbcL*
ITS+trnH+*rbcL* vs *matK*+*rbcL*	0.0003333	0.4903	P > 0.05	-0.001979 to 0.002646	ITS+*trnH-psbA*+*rbcL*=*matK*+*rbcL*
ITS+trnH+*rbcL* vs trnH+*matK*	0.00025	0.3678	P > 0.05	-0.002062 to 0.002562	ITS+*trnH-psbA*+*rbcL*=*trnH-psbA*+*matK*
ITS+trnH+*rbcL* vs trnH+*matK*+*rbcL*	0.00025	0.3678	P > 0.05	-0.002062 to 0.002562	ITS+*trnH-psbA*+*rbcL*=*trnH-psbA*+*matK*+*rbcL*
ITS+trnH+*rbcL* vs trnH+*rbcL*	0.0003333	0.4903	P > 0.05	-0.001979 to 0.002646	ITS+*trnH-psbA*+*rbcL*=*trnH-psbA*+*rbcL*
matK+*rbcL* vs trnH+*matK*	-0.00008333	0.1226	P > 0.05	-0.002396 to 0.002229	*matK*+*rbcL*=*trnH-psbA*+*matK*
matK+*rbcL* vs trnH+*matK*+*rbcL*	-0.00008333	0.1226	P > 0.05	-0.002396 to 0.002229	*matK*+*rbcL*=*trnH-psbA*+*matK*+*rbcL*
matK+*rbcL* vs trnH+*rbcL*	0	0	P > 0.05	-0.002312 to 0.002312	*matK*+*rbcL*=*trnH-psbA*+*rbcL*
trnH+*matK* vs trnH+*matK*+*rbcL*	0	0	P > 0.05	-0.002312 to 0.002312	*trnH-psbA*+*matK*=*trnH-psbA*+*matK*+*rbcL*
trnH+*matK* vs trnH+*rbcL*	0.00008333	0.1226	P > 0.05	-0.002229 to 0.002396	*trnH-psbA*+*matK*=*trnH-psbA*+*rbcL*
trnH+*matK*+*rbcL* vs trnH+*rbcL*	0.00008333	0.1226	P > 0.05	-0.002229 to 0.002396	*trnH-psbA*+*matK*+*rbcL*=*trnH-psbA*+*rbcL*
