# Supplementary material for: Universal Plant DNA Barcode Loci May Not Work in Complex Groups: A Case Study with Indian Berberis Species
Source: PLoS One. 2010 Oct 27;5(10):e13674. doi: 10.1371/journal.pone.0013674 (PMC2965122; doi:10.1371/journal.pone.0013674)
Supplement: Table S6 — Results of paired t-test to compare between minimum inter and maximum intraspecific K2P distances of different loci. (0.06 MB PDF) [file pone.0013674.s012.pdf]

Table S6

	Locus	p value	t, df	Mean of differences (Interspecific-Intraspecific)
Berberis	ITS	0.0012	t=3.978, df=15	-0.00775
	matK	0.0081	t=3.167, df=12	-0.002923
	rbcL	0.0824	t=1.871, df=14	-0.0004
	trnH-psbA	0.3383	t=0.9941, df=13	-0.001357
Ficus	ITS	0.02	t=2.763, df=10	0.03191
	matK	0.1772	t=1.480, df=8	-0.003
	rbcL	0.0425	t=2.324, df=10	0.0008182
	trnH-psbA	0.0065	t=3.421, df=10	0.006364
Gossypium	ITS	0.8614	t=0.1901, df=3	-0.0005
	matK	0.0455	t=3.308, df=3	-0.01075
	rbcL	0.0957	t=2.402, df=3	-0.0025
	trnH-psbA	0.1522	t=1.910, df=3	-0.00325
