# Supplementary material for: Universal Plant DNA Barcode Loci May Not Work in Complex Groups: A Case Study with Indian Berberis Species
Source: PLoS One. 2010 Oct 27;5(10):e13674. doi: 10.1371/journal.pone.0013674 (PMC2965122; doi:10.1371/journal.pone.0013674)
Supplement: Table S7 — Minimum inter- and maximum intraspecific K2P distances of Berberis species for different loci and ability to discriminate species. These values could not be calculated in some cases (-) where there was either single accession or sequencing failure for the locus. (0.08 MB PDF) [file pone.0013674.s013.pdf]

Table S7

Species	ITS			*rbcL*			*matK*			*trnH-psbA*		
	Minimum of inter-specific K2P distance	Maximum of intra-specific K2P distance	Species resolution	Minimum of inter-specific K2P distance	Maximum of intra-specific K2P distance	Species resolution	Minimum of inter-specific K2P distance	Maximum of intra-specific K2P distance	Species resolution	Minimum of inter-specific K2P distance	Maximum of intra-specific K2P distance	Species resolution
B. angulosa	0.0	0.0	No	0.0	-	-	0.0	0.0	No	0.0	0.0	No
B. aristata	0.0	0.014	No	0.0	0.002	No	0.0	0.0	No	0.0	0.0	No
B. asiatica	0.0	0.022	No	0.0	0.002	No	0.0	0.006	No	0.0	0.0	No
B. chitria	0.0	0.014	No	0.0	0.0	No	0.0	0.008	No	0.0	0.008	No
B. glaucocarpa	0.0	0.012	No	0.0	0.0	No	0.0	0.01	No	0.0	0.008	No
B. griffithiana	0.0	0.008	No	0.0	-	-	-	-	-	-	-	-
B. hainesii	0.0	0.004	No	0.0	0.0	No	0.0	0.004	No	0.0	0.0	No
B. insignis	0.0	0.01	No	0.0	0.0	No	0.0	0.0	No	0.0	0.002	No
B. jaeschkeana	0.0	0.0	No	0.0	0.0	No	0.0	0.0	No	0.0	0	No
B. lycium	0.0	0.012	No	0.0	0.0	No	0.0	0.0	No	0.0	0.008	No
B. macrosepala	0.0	0.0	No	-	-	-	-	-	-	-	-	-
B. pachyacantha	0.008	0.0	Yes	0.0	0.0	No	0.004	0.006	No	0.015	0.003	Yes
B. replicata	0.0	0.016	No	0.0	0.0	No	0.0	0.002	No	0.0	0.0	No
B. tinctoria	0.0	0.008	No	0.0	0.0	No	0.0	0.002	No	0.0	0.0	No
B. umbellata	0.0	0.012	No	0.0	0.002	No	0.0	0.004	No	0.0	0.005	No
B. wightiana	0.0	0.0	No	0.0	0.0	No	0.0	0.0	No	0.0	0.0	No
