# Supplementary material for: Universal Plant DNA Barcode Loci May Not Work in Complex Groups: A Case Study with Indian Berberis Species
Source: PLoS One. 2010 Oct 27;5(10):e13674. doi: 10.1371/journal.pone.0013674 (PMC2965122; doi:10.1371/journal.pone.0013674)
Supplement: Table S8 — One way ANOVA with Bonferroni's multiple comparison tests to compare inter (A, Ficus and C, Gossypium) and intraspecific (B, Ficus and D, Gossypium) variability for each individual locus. (0.03 MB PDF) [file pone.0013674.s014.pdf]

Table S8

A

Overall p<0.0001					
Bartlett's test for equal variances p=0.13					
Bonferroni's Multiple Comparison Test	Mean Diff.	t	P value	95% CI of diff	Result
ITS vs matK	0.9848	12.6	P < 0.001	0.7669 to 1.203	ITS>>matK
ITS vs rbcL	1.683	23.46	P < 0.001	1.483 to 1.883	ITS>>rbcL
ITS vs trnH-psbA	0.6479	9.03	P < 0.001	0.4479 to 0.8479	ITS>>trnH-psbA
matK vs rbcL	0.6983	8.932	P < 0.001	0.4804 to 0.9163	matK>>rbcL
matK vs trnH-psbA	-0.3369	4.31	P < 0.001	-0.5549 to -0.1190	trnH-psbA>>matK
rbcL vs trnH-psbA	-1.035	14.43	P < 0.001	-1.235 to -0.8353	trnH-psbA>>rbcL

B

Overall p=0.0019					
Bartlett's test for equal variances p=ns					
Bonferroni's Multiple Comparison Test	Mean Diff.	t	P value	95% CI of diff	Result
ITS vs matK	0.00009091	0.06372	P > 0.05	-0.003922 to 0.004104	ITS=matK
ITS vs rbcL	0.004091	3.33	P < 0.05	0.0006364 to 0.007545	rbcL>ITS
ITS vs trnH-psbA	0.004091	3.238	P < 0.05	0.0005373 to 0.007645	trnH-psbA>ITS
matK vs rbcL	0.004	2.755	P > 0.05	-0.00008282 to 0.008083	matK=rbcL
matK vs trnH-psbA	0.004	2.7	P > 0.05	-0.0001670 to 0.008167	trnH-psbA=matK
rbcL vs trnH-psbA	0	0	P > 0.05	-0.003633 to 0.003633	trnH-psbA=rbcL

C

Overall p=0.0019					
Bartlett's test for equal variances p=0.15					
Bonferroni's Multiple Comparison Test	Mean Diff.	t	P value	95% CI of diff	Result
ITS vs matK	0.8846	36.28	P < 0.001	0.8078 to 0.9615	ITS >> matK
ITS vs rbcL	0.9368	38.42	P < 0.001	0.8599 to 1.014	ITS >> rbcL
ITS vs trnH-psbA	0.8771	35.97	P < 0.001	0.8002 to 0.9540	ITS >> trnH-psbA
matK vs rbcL	0.05215	2.139	P > 0.05	-0.02472 to 0.1290	matK = rbcL
matK vs trnH-psbA	-0.007543	0.3094	P > 0.05	-0.08441 to 0.06933	matK = trnH-psbA
rbcL vs trnH-psbA	-0.05969	2.448	P > 0.05	-0.1366 to 0.01718	rbcL = trnH-psbA

D

Overall p=0.286					
Bartlett's test for equal variances p=0.31					
Bonferroni's Multiple Comparison Test	Mean Diff.	t	P value	95% CI of diff	Result
ITS vs matK	-0.0005	0.541	P > 0.05	-0.003414 to 0.002414	ITS = matK
ITS vs rbcL	0.00125	1.353	P > 0.05	-0.001664 to 0.004164	ITS = rbcL
ITS vs trnH-psbA	0.00075	0.8115	P > 0.05	-0.002164 to 0.003664	ITS = trnH-psbA
matK vs rbcL	0.00175	1.894	P > 0.05	-0.001164 to 0.004664	matK = rbcL
matK vs trnH-psbA	0.00125	1.353	P > 0.05	-0.001664 to 0.004164	matK = trnH-psbA
rbcL vs trnH-psbA	-0.0005	0.541	P > 0.05	-0.003414 to 0.002414	rbcL = trnH-psbA
