# Supplementary material for: Universal Plant DNA Barcode Loci May Not Work in Complex Groups: A Case Study with Indian Berberis Species
Source: PLoS One. 2010 Oct 27;5(10):e13674. doi: 10.1371/journal.pone.0013674 (PMC2965122; doi:10.1371/journal.pone.0013674)
Supplement: Table S9 — Minimum inter- and maximum intraspecific K2P distances of species of Ficus and Gossypium for different loci and ability to discriminate species. These values could not be calculated in some cases (-) where there was either single accession or sequencing failure for the locus. (0.09 MB PDF) [file pone.0013674.s015.pdf]

| Species | ITS | | | *rbcL* | | | *matK* | | | *trnH-psbA* | | |
|---|---|---|---|---|---|---|---|---|---|---|---|---|
| | Minimum of inter-specific K2P distance | Maximum of intra-specific K2P distance | Species resolution | Minimum of inter-specific K2P distance | Maximum of intra-specific K2P distance | Species resolution | Minimum of inter-specific K2P distance | Maximum of intra-specific K2P distance | Species resolution | Minimum of inter-specific K2P distance | Maximum of intra-specific K2P distance | Species resolution |
| ***F. benghalensis*** | 0.021 | 0.006 | Yes | 0.0 | 0.0 | No | 0.0 | 0.005 | No | 0.003 | 0.0 | Yes |
| ***F. benjamina*** | 0.015 | 0.011 | Yes | 0.002 | 0.0 | Yes | 0.0 | 0.0 | No | 0.003 | 0.0 | Yes |
| ***F. carica*** | 0.048 | 0.0 | Yes | 0.003 | 0.0 | Yes | 0.003 | 0.0 | Yes | 0.003 | 0.0 | Yes |
| ***F. elastica*** | 0.013 | 0.0 | Yes | 0.002 | 0.0 | Yes | - | - | No | 0.003 | 0.0 | Yes |
| ***F. glomerata*** | 0.154 | 0.021 | Yes | 0.0 | 0.0 | No | 0.0 | 0.015 | No | 0.007 | 0.0 | Yes |
| ***F. hispida*** | 0.055 | 0.0 | Yes | 0.0 | 0.0 | No | 0.003 | 0.008 | No | 0.024 | 0.0 | Yes |
| ***F. religiosa*** | 0.008 | 0.008 | Yes | 0.0 | 0.0 | No | 0.0 | 0.008 | No | 0.007 | 0.0 | Yes |
| ***F. retusa*** | 0.013 | 0.004 | Yes | 0.0 | 0.0 | No | 0.0 | 0.002 | No | 0.003 | 0.0 | yes |
| ***F. rumphi*** | 0.039 | 0.008 | Yes | 0.0 | 0.0 | No | - | - | - | 0.007 | 0.0 | Yes |
| ***F. trigona*** | 0.037 | 0.0 | Yes | 0.0 | 0.0 | No | - | - | - | 0.007 | 0.0 | Yes |
| ***F. virens*** | 0.008 | 0.002 | Yes | 0.002 | 0.0 | Yes | 0.005 | 0.0 | Yes | 0.003 | 0.0 | Yes |
| ***G. arboreum*** | 0.005 | 0.005 | No | 0.0 | 0.005 | No | 0.0 | 0.02 | 0.0 | 0.0 | 0.0 | No |
| ***G. barbadense*** | 0.004 | 0.002 | Yes | 0.0 | 0.002 | No | 0.0 | 0.01 | 0.0 | 0.003 | 0.005 | No |
| ***G. herbaceum*** | 0.005 | 0.013 | No | 0.0 | 0.003 | No | 0.0 | 0.008 | 0.0 | 0.0 | 0.003 | No |
| ***G. hirsutum*** | 0.004 | 0.0 | Yes | 0.0 | 0.0 | No | 0.0 | 0.005 | 0.0 | 0.0 | 0.008 | No |
