# Supplementary material for: Universal Plant DNA Barcode Loci May Not Work in Complex Groups: A Case Study with Indian Berberis Species
Source: PLoS One. 2010 Oct 27;5(10):e13674. doi: 10.1371/journal.pone.0013674 (PMC2965122; doi:10.1371/journal.pone.0013674)
Supplement: Table S10 — List of Accessions and DNA numbers of different plant species along with Global Positioning System (GPS) data, and collector's name. In some cases GPS data could not be taken. (0.09 MB PDF) [file pone.0013674.s016.pdf]

Table S10

Berberis

Species	Collection No.	DNA No.	Latitude / Longitude	Region	collectors name	Herbarium
Berberis angulosa	249309	B121	N 27º 27' 77"E 92º 06' 50"	Arunachal Pradesh: W. Kameng Dist., Sangrila on Tawang Rd. , Alt. 3329 m	BD, KNN, HS, AT	NBRI (LWG)
Berberis angulosa	249310	B122	N 27º 27' 77" / E 92º 06' 50"	Arunachal Pradesh: W. Kameng Dist., near Sangrila on Tawang Rd. , Alt. 3329 m	BD, KNN, HS, AT	NBRI (LWG)
Berberis angulosa	249312	B124	N 27º 27' 77" / E 92º 06' 60"	Arunachal Pradesh: W. Kameng Dist., near Baisakhi on Tawang Rd., Alt. 3346 m	BD, KNN, HS, AT	NBRI (LWG)
Berberis angulosa	249313	B125	N 27º 27' 80" / E 92º 06' 57"	Arunachal Pradesh: W. Kameng Dist., 2 km above Sangrila, near CSD Canteen on Tawang Rd., Alt. 3310 m	BD, KNN, HS, AT	NBRI (LWG)
Berberis aristata	248162	B51	N 29º 58' 19" / E 80º 39' 27"	Uttarakhand: Pithoragarh Dist., Vicinity of Narayan ashram, Alt. 2556 m	BD, HS, AT	NBRI (LWG)
Berberis aristata	248166	B52	N 29º 58' 23" / E 80º 39' 27"	Uttarakhand: Pithoragarh Dist., Vicinity of Narayan ashram, Alt. 2593 m	BD, HS, AT	NBRI (LWG)
Berberis aristata	248169	B53	N 29º 58' 23" / E 80º 39' 27"	Uttarakhand: Pithoragarh Dist., Vicinity of Narayan ashram, Alt. 2586 m	BD, HS, AT	NBRI (LWG)
Berberis aristata	248170	B54	N 29º 58' 21" / E 80º 39' 29"	Uttarakhand: Pithoragarh Dist., Vicinity of Narayan ashram, Alt. 2604 m	BD, HS, AT	NBRI (LWG)
Berberis aristata	248172	B55	N 29º 58' 28" / E 80º 39' 60"	Uttarakhand: Pithoragarh Dist., Vicinity of Narayan ashram, Alt. 2576 m	BD, HS, AT	NBRI (LWG)
Berberis aristata	248175	B56	N 29º 58' 30" / E 80º 39' 40"	Uttarakhand: Pithoragarh Dist., Vicinity of Narayan ashram, Alt. 2610 m	BD, HS, AT	NBRI (LWG)
Berberis aristata	248176	B57	N 29º 58' 80" / E 80º 39' 31"	Uttarakhand: Pithoragarh Dist., Vicinity of Narayan ashram, Alt. 2586 m	BD, HS, AT	NBRI (LWG)
Berberis aristata	248178	B58	N 29º 58' 34" / E 80º 39' 31"	Uttarakhand: Pithoragarh Dist., Vicinity of Narayan ashram, Alt. 2587 m	BD, HS, AT	NBRI (LWG)
Berberis aristata	248163	B59	NA	Uttarakhand: Pithoragarh Dist., Vicinity of Narayan ashram, Alt. 2560 m	BD, HS, AT	NBRI (LWG)
Berberis aristata	248171	B60	N 29º 58' 24" / E 80º 09' 29"	Uttarakhand: Pithoragarh Dist., Vicinity of Narayan ashram, Alt. 2550 m	BD, HS, AT	NBRI (LWG)
Berberis asiatica	249337	B133	N 27º 12' 84" / E 92º 24' 88"	Arunachal Pradesh: W. Kameng Dist., 16 km before Bomdila, Alt. 1505 m	BD, TH, HS, AT	NBRI (LWG)
Berberis asiatica	249338	B134	N 27º 12' 84" / E 92º 24' 88"	Arunachal Pradesh: W. Kameng Dist., 16 km before Bomdila, Alt. 1505 m	BD, TH, HS, AT	NBRI (LWG)
Berberis asiatica	249340	B135	N 27º 12' 75" / E 92º 28' 18"	Arunachal Pradesh: W. Kameng Dist., near Tenga Army Base, along riverside , Alt. 1503 m	BD, TH, HS, AT	NBRI (LWG)
Berberis asiatica	248130	B16	N 29º 23' 94" / E 79º 27' 55"	Uttarakhand: Near Langham Girls Hostel., Nainital, Alt. 1995 m	BD, TH, HS, AT	NBRI (LWG)
Berberis asiatica	248132	B18	N 29º 37' 0 8" / E 79º 26' 76"	Uttarakhand: Near Jhula Devi Tmple, Ranikhet, Almora Dist., Alt. 1991 m	BD, TH, HS, AT	NBRI (LWG)
Berberis asiatica	248137	B23	N 30º 32' 83" / E 79º 57' 16"	Uttarakhand: Near Chitai, Almora Dist., Alt. 1810 m	BD, TH, HS, AT	NBRI (LWG)
Berberis asiatica	248139	B25	N 30º 56' 42" / E 79º42' 28"	Uttarakhand: Binsar road, Almora Dist., Alt. 1881 m	BD, TH, HS, AT	NBRI (LWG)
Berberis asiatica	248140	B26	N 29º 32' 87" / E 79º 32' 85"	Uttarakhand: uyalbari en route Almora, Alt. 1016 m	BD, TH, HS, AT	NBRI (LWG)
Berberis asiatica	248141	B27	N 29º 23' 79" / E 79º 30' 76"	Uttarakhand: Khairna, Almora Dist., Alt. 1460 m	BD, TH, HS, AT	NBRI (LWG)
Berberis asiatica	248142	B28	N 29º 23' 87" / E 79º 30' 76"	Uttarakhand: Niglat, Almora Dist., Alt. 1500 m	BD, TH, HS, AT	NBRI (LWG)
Berberis asiatica	248144	B30	N 29º 23' 46" / E 79º 30' 73"	Uttarakhand: Bhawali, Nainital. Dist., Alt. 1653 m	BD, TH, HS, AT	NBRI (LWG)
Berberis asiatica	248145	B31	N 29º 23' 34" / E 79º 30' 92"	Uttarakhand: Bhawali en route Bhimtal, Nainital. Dist., Alt. 1683 m	BD, TH, HS, AT	NBRI (LWG)
Berberis asiatica	250017	B32	N 29º 22' 692" / E 079º 28' 822"	Uttarakhand: Jeolikote	BD, TH, HS, AT	NBRI (LWG)
Berberis asiatica	250028	B38	N 29º 26' 838" / E 079º 28' 977"	Uttarakhand: On way to Almorah, near Kaichi Temple	BD, TH, HS, AT	NBRI (LWG)
Berberis asiatica	250029	B39	N 29º 26' 838" / E 079º 28' 977"	Uttarakhand: On way to Almorah, near Kaichi Temple	BD, TH, HS, AT	NBRI (LWG)
Berberis asiatica	250037	B40	N 29º 51' 273" / E 079º 35' 708"	Uttarakhand: Kausani	BD, TH, HS, AT	NBRI (LWG)
Berberis asiatica	248146	B42	N 29º 41' 43" / E 80º 12' 43"	Uttarakhand: Pithoragarh Dist., Devalthal, Alt. 1283 m	BD, TH, HS, AT	NBRI (LWG)
Berberis asiatica	248147	B43	N 29º 57' 55" / E 80º 09' 32"	Uttarakhand: Pithoragarh Dist., Nachini enroute Munsiyari, Alt. 1340 m	BD, TH, HS, AT	NBRI (LWG)
Berberis asiatica	248148	B44	N 30º 03' 55" / E 80º 13' 64"	Uttarakhand: Pithoragarh Dist., Patalthaur enroute Munsiyari, Alt. 2260 m	BD, TH, HS, AT	NBRI (LWG)
Berberis asiatica	248149	B45	N 30º 03' 59" / E 80º 13' 72"	Uttarakhand: Pithoragarh Dist., Patalthaur near Medicinal plants nursery, Alt. 2260 m	BD, TH, HS, AT	NBRI (LWG)
Berberis asiatica	248155	B47	N 30º 05' 09" / E 80º 15' 71"	Uttarakhand: Pithoragarh Dist., Madkote, Alt. 1517 m	BD, TH, HS, AT	NBRI (LWG)
Berberis asiatica	248156	B48	N 30º 00' 69" / E 80º 19' 34"	Uttarakhand: Pithoragarh Dist., Seraghat, Alt. 1130 m	BD, TH, HS, AT	NBRI (LWG)
Berberis asiatica	248157	B49	NA	Uttarakhand: Pithoragarh Dist. Khet on way to Narayan Ashram.	BD, TH, HS, AT	NBRI (LWG)
Berberis asiatica	248159	B50	N 29º 59' 22" / E 80º 37' 15"	Uttarakhand: Pithoragarh Dist. Uttarakhand: , Pangu enroute Narayan Ashram, Alt. 2176 m	BD, TH, HS, AT	NBRI (LWG)
Berberis asiatica	215201	B7	N 29º 32' 87" / E 079º 32' 85"	Uttarakhand: Almora, on way to Karnataka kholu	BD, TH, HS, AT	NBRI (LWG)
Berberis asiatica	248159A	B74	N 29º 59' 22" / E 80º 37' 15"	Uttarakhand: Pithoragarh Dist., pangu enroute Narayan ashram, Alt. 2176 m	BD, TH, HS, AT	NBRI (LWG)
Berberis asiatica	215202	B8	N 29º 32' 87" / E 079º 32' 85"	Uttarakhand: Almora	BD, TH, HS, AT	NBRI (LWG)
Berberis asiatica	215203	B9	N 29º 23' 15" / E 079º 30' 64"	Uttarakhand: Mehra gaon, near Bhimtal	BD, TH, HS, AT	NBRI (LWG)
Berberis chitria	228469	14	NA	Himachal pradesh: Gulaba	BD, TH, HS, AT	NBRI (LWG)
Berberis chitria	228437	93	N 29o 36.260' / E 080o 11.597'	Uttarakhand: Pithoragarh	BD, TH, HS, AT	NBRI (LWG)
Berberis chitria	250061	B105	N 29º 25' 321" / E 079º 26' 146"	Uttarakhand: Kilberry	BD, TH, HS, AT	NBRI (LWG)
Berberis chitria	250062	B106	N 29º 22' 920" / E 079º 26' 223"	Uttarakhand: On way to Ramnagar from Nainital	BD, TH, HS, AT	NBRI (LWG)
Berberis chitria	250068	B112	N 29º 24' 397" / E 079º 26' 384"	Uttarakhand: Kumaon University Campus	BD, TH, HS, AT	NBRI (LWG)
Berberis chitria	248128	B14	N 29º 23' 14" / E 79º 26' 61"	Uttarakhand: On way to Laria Kanta, Nainital, Alt. 2093 m	BD, TH, HS, AT	NBRI (LWG)
Berberis chitria	249380	B175	N 30º 57' 52" / E 78º 25' 26"	Uttarakhand: 3 km away from Mussorie on Kempty Fall road, Alt. 2358 m	BD, TH, HS, AT	NBRI (LWG)
Berberis chitria	248133	B19	N 29º 37'10" / E 79º 26' 30"	Uttarakhand: Near Jhula Devi Temple, Ranikhet, Almora Dist., Alt. 1991 m	BD, TH, HS, AT	NBRI (LWG)
Berberis chitria	248134	B20	N 29º 37' 52" / E 79º 26' 60"	Uttarakhand: Chaubatiya Forest, Ranikhet, Almora Dist., Alt. 1984 m	BD, TH, HS, AT	NBRI (LWG)
Berberis chitria	248135	B21	N 29º 37' 87" / E 79º 26' 79"	Uttarakhand: Between Chaubatiya & Pilkholi, Ranikhet, Almora Dist., Alt. 1961 m	BD, TH, HS, AT	NBRI (LWG)
Berberis chitria	248138	B24	N 30º 56' 47" / E 79º42' 34"	Uttarakhand: Binsar road, Almora Dist., Alt. 1889 m	BD, TH, HS, AT	NBRI (LWG)
Berberis chitria	250018	B33	N 29º 24' 326" / E 079º 26' 798"	Uttarakhand: Back side of China Peak	BD, TH, HS, AT	NBRI (LWG)
Berberis chitria	250019	B34	N 29º 24' 370" / E 079º 26' 824"	Uttarakhand: Back side of China Peak	BD, TH, HS, AT	NBRI (LWG)
Berberis chitria	250020	B35	N 29º 23' 568" / E 079º 26' 292"	Uttarakhand: Near Government Polytechnic	BD, TH, HS, AT	NBRI (LWG)
Berberis chitria	250038	B41	N 29º 51' 273" / E 079º 35' 708"	Uttarakhand: Kausani	BD, TH, HS, AT	NBRI (LWG)
Berberis chitria	248150	B61	N 30º 03' 59" / E 80º 13' 74"	Uttarakhand: Pithoragarh Dist., 5 km before Munsiyari, Alt. 2275 m	BD, TH, HS, AT	NBRI (LWG)
Berberis chitria	248151	B62	N 30º 02' 15" / E 80º 11' 61"	Uttarakhand: Pithoragarh Dist., between Birthi Fall & Kalamuni enroute Munsiyari, Alt. 2260 m	BD, TH, HS, AT	NBRI (LWG)
Berberis chitria	248152	B63	N 30º 02' 21" / E 80º 11' 98"	Uttarakhand: Pithoragarh Dist., Kalamuni Munsiyari, Alt. 2730 m	BD, TH, HS, AT	NBRI (LWG)
Berberis chitria	248153	B64	N 29º 02' 40" / E 80º 11' 55"	Uttarakhand: Pithoragarh Dist., Kalamuni on way to Khulia Top, Alt. 2795 m	BD, TH, HS, AT	NBRI (LWG)
Berberis chitria	248179	B73	N 29º 58' 36" / E 80º 39' 32"	Uttarakhand: Pithoragarh Dist., Vicinity of Narayan Ashram, Alt. 2592 m	BD, TH, HS, AT	NBRI (LWG)
Berberis glaucocarpa	248028	16	NA	Uttarakhand: 1/2 km away from Jankichatti, Uttarkashi Dist.	BD, HS, AT	NBRI (LWG)
Berberis glaucocarpa	248026	46	NA	Uttarakhand: 4 km before Yamnotri, Uttarkashi Dist.	BD, HS, AT	NBRI (LWG)
Berberis glaucocarpa	248019	47	N 30º 57'. 50 / E 78º 25'. 37	Uttarakhand: Between Hanumanchatti & Phoolchatti, Uttarkashi Dist.	BD, HS, AT	NBRI (LWG)
Berberis glaucocarpa	248092	101	NA	Uttarakhand: 4 km before Yamnotri, Uttarkashi Dist.	BD, HS, AT	NBRI (LWG)
Berberis glaucocarpa	250063	B107	N 29º 24' 393" / E 079º 26' 394"	Uttarakhand: China Peak	BD, HS, AT	NBRI (LWG)
Berberis glaucocarpa	250064	B108	N 29º 25' 399" / E 079º 26' 385"	Uttarakhand: China Peak	BD, HS, AT	NBRI (LWG)
Berberis glaucocarpa	250065	B109	N 29º 23' 379" / E 079º 26' 710"	Uttarakhand: Near China Peak	BD, HS, AT	NBRI (LWG)
Berberis glaucocarpa	250066	B110	N 29º 23' 397" / E 079º 26' 385"	Uttarakhand: On the top of China Peak	BD, HS, AT	NBRI (LWG)
Berberis glaucocarpa	250067	B111	N 29º 23' 379" / E 079º 26' 710"	Uttarakhand: On the top of China Peak	BD, HS, AT	NBRI (LWG)
Berberis glaucocarpa	249371	B168	N 30º 58' 20" / E 78º 26' 11"	Uttarakhand: Jankichatti, Himri forest across Kharsali Village Alt. 2534 m	BD, HS, AT	NBRI (LWG)
Berberis glaucocarpa	249372	B169	N 30º 58' 29" / E 78º 26' 20"	Uttarakhand: Jankichatti, Himri forest across Kharsali Village Alt. 2544 m	BD, HS, AT	NBRI (LWG)
Berberis glaucocarpa	249374	B170	N 30º 58' 40" / E 78º 25' 99"	Uttarakhand: Jankichatti, Alt. 2517 m	BD, HS, AT	NBRI (LWG)
Berberis glaucocarpa	249375	B171	N 30º 57' 50" / E 78º 25' 36"	Uttarakhand: Between Jankichatti & Phoolchatti, Alt. 2503 m	BD, HS, AT	NBRI (LWG)
Berberis glaucocarpa	248028A	B76	N 30º 58' 015" / E 078º 25' 088"	Uttarakhand: 1/2 Km away from Jankichatti Uttarkashi Dist.	BD, HS, AT	NBRI (LWG)
Berberis griffithiana	249335	B131	N 27° 16' 39" / E 92° 25' 62"	Arunachal Pradesh: W.Kameng Dist., 1Km away from Bomdila on Tawang road Alt. 1875m	BD, KNN, HS, AT	NBRI (LWG)
Berberis griffithiana	248186	B138	N 27º 32' 75" / E 93º 48' 74'	Arunachal Pradesh: Subansiri Dist., near Hapoli, Alt. 1672 m	BD, KNN, HS, AT	NBRI (LWG)
Berberis griffithiana	248189	B141	N 27º 35' 72" / E 93º 49' 51"	Arunachal Pradesh: Subansiri Dist., Ziro valley, above Fire Station, Alt. 1597 m	BD, KNN, HS, AT	NBRI (LWG)
Berberis griffithiana	248193	B145	N 27º 35' 72" / E 90º 49' 51'	Arunachal Pradesh: Subansiri Dist., Ziro valley, above Fire Station, Alt. 1597 m	BD, KNN, HS, AT	NBRI (LWG)
Berberis hainseii	250070	B150	N 22º 27' 472" / E 078º 23' 830"	Madhya Pradesh: Pachmarhi, On way to Dhoopgarh	TH,VS	NBRI (LWG)
Berberis hainseii	250071	B151	N 22º 27' 453" / E 078º 23' 833"	Madhya Pradesh: Pachmarhi, On way to Dhoopgarh Near Airport	TH,VS	NBRI (LWG)
Berberis hainseii	250072	B152	N 22º 27' 456" / E 078º 23' 833"	Madhya Pradesh: Pachmarhi, Jalgali	TH,VS	NBRI (LWG)
Berberis hainseii	250073	B153	N 22º 27' 263" / E 078º 23' 719"	Madhya Pradesh: Pachmarhi, Dhoopgarh	TH,VS	NBRI (LWG)
Berberis hainseii	250075	B155	N 22º 27' 299" / E 078º 24' 871"	Madhya Pradesh: Pachmarhi, Dutches fall	TH,VS	NBRI (LWG)
Berberis hainseii	250076	B156	N 22º 27' 510" / E 078º 23' 821"	Madhya Pradesh: Pachmarhi, Handi Khoh	TH,VS	NBRI (LWG)
Berberis hainseii	250077	B157	N 22º 27' 513" / E 078º 23' 839"	Madhya Pradesh: Pachmarhi, Handi Khoh	TH,VS	NBRI (LWG)
Berberis insignis	249328	B127	N 27º 16' 32" / E 92º 25' 62"	Arunachal Pradesh: W. Kameng Dist., 1km away from Bomdila on Tawang road, Alt. 1875 m	BD, KNN	NBRI (LWG)
Berberis insignis	249329	B128	N 27º 16' 32" / E 92º 25' 62"	Arunachal Pradesh: W. Kameng Dist., 1km away from Bomdila on Tawang road, Alt. 1875 m	BD, KNN	NBRI (LWG)
Berberis insignis	249330	B129	N 27º 16' 32" / E 92º 25' 62"	Arunachal Pradesh: W. Kameng Dist., 1km away from Bomdila on Tawang road, Alt. 1875 m	BD, KNN	NBRI (LWG)
Berberis jaeschkeana	250084	B176	N 32º 22' 015" / E 077º 17' 805"	Himachal pradesh: On way from Chhatru to Koksar	TH	NBRI (LWG)
Berberis jaeschkeana	250082	B178	N 32º 21' 362" / E 077º 13' 407"	Himachal pradesh: On way to Rohtang from Marhi	TH	NBRI (LWG)
Berberis jaeschkeana	250086	B182	N 32º 27' 798" / E 077º 08' 634"	Himachal pradesh: 20 mile from Koksar to Sisoo	TH	NBRI (LWG)
Berberis jaeschkeana	250087	B183	N 32º 28' 615" / E 077º 07' 105"	Himachal pradesh: Sisoo to Gondla	TH	NBRI (LWG)
Berberis jaeschkeana	250083	B185	NA	Himachal pradesh: On way to Rohtang from Marhi	TH	NBRI (LWG)
Berberis jaeschkeana	250085	B186	N 32º 24' 776' / E 077º 14' 147"	Himachal pradesh: Koksar	TH	NBRI (LWG)
Berberis jaeschkeana	228471	B79	N 32º 22' 015" / E 077º 17' 805"	Himachal pradesh: :On way from Koksar to Chhatru	TH	NBRI (LWG)
Berberis jaeschkeana	248475	B80	N 32º 30' 127" / E 077º 03' 347"	Himachal pradesh: :Chhota Dara	TH	NBRI (LWG)
Berberis jaeschkeana	228477	B81	N 32º 30' 127" / E 077º 03' 100"	Himachal pradesh: :On way from tandi to gondla	TH	NBRI (LWG)
Berberis lycium	228485	3	NA	Himachal pradesh: Raisan on Kullu - Manali road., Himachal pradesh:	BD, HS, AT	NBRI (LWG)
Berberis lycium	228463	11	N 32o 20.474' / E 077o 13.011'	Himachal pradesh: Above Marhi	BD, HS, AT	NBRI (LWG)
Berberis lycium	248100	31	N 31º18.688' / E 77º 10. 68'	Himachal pradesh: 2 km before Kothi, Kullu Dist.	BD, HS, AT	NBRI (LWG)
Berberis lycium	248103	34	N 32º 15'. 05 / E 77º 11'. 40	Himachal pradesh: Near Kothi, Kullu Dist.	BD, HS, AT	NBRI (LWG)
Berberis lycium	248104	35	N 31º 06'. 42 / E 77º 37'. 77	Himachal pradesh: Alu ground on Kullu - Manali road., Himachal pradesh:	BD, HS, AT	NBRI (LWG)
Berberis lycium	248105	36	N 32º 03. 81' / E 77º 08. 43'	Himachal pradesh: Raisan on Kullu - Manali road., Himachal pradesh:	BD, HS, AT	NBRI (LWG)
Berberis lycium	248067	70	N 30º 52.60' / E 78º 05. 09'	Uttarakhand: Purola, Uttarkashi Dist.	BD, HS, AT	NBRI (LWG)
Berberis lycium	248049	74	N 30º 59. 86' / E 78º 41. 70'	Uttarakhand: Sukhi on way to Gangotri, Uttarkashi Dist.	BD, HS, AT	NBRI (LWG)
Berberis lycium	248094	75	N 31º 42. 37' / E 77º 01. 79'	Himachal pradesh: Sambal, Mandi,	BD, HS, AT	NBRI (LWG)
Berberis lycium	248076	80	N 38º 56.41' / E 77º 50'. 95	Uttarakhand: 5 km away from Tiuni on way to Jubbal, Dehra Dun Dist.	BD, HS, AT	NBRI (LWG)
Berberis lycium	248085	84	N 31º 05. 91' / E 77º 22. 78'	Himachal pradesh: Near Theog, Shimla Dist.	BD, HS, AT	NBRI (LWG)
Berberis lycium	248123	87	N 30º 20.8' / E 78º 00. 82'	Uttarakhand: B.S.I. Garden, Dehra Dun	BD, HS, AT	NBRI (LWG)
Berberis lycium	248124	88	N 30º 20'.8 / E 78º 00'. 82	Uttarakhand: B.S.I. Garden, Dehra Dun	BD, HS, AT	NBRI (LWG)
Berberis lycium	250001	B1	N 32º 59' 328" / E 074º 55' 596"	Jammu & Kashmir: Trikuta Hills	BD, HS, AT	NBRI (LWG)
Berberis lycium	250002	B2	N 32º 60' 141" / E 075º 14' 568"	Jammu & Kashmir: 20 Km before Kud	BD, HS, AT	NBRI (LWG)
Berberis lycium	250003	B3	N 33º 03' 828" / E 075º 17' 694"	Jammu & Kashmir: 5 Km before Kud	BD, HS, AT	NBRI (LWG)
Berberis lycium	250004	B4	N 33º 04' 452" / E 075º 17' 628"	Jammu & Kashmir: Kud	BD, HS, AT	NBRI (LWG)
Berberis lycium	250005	B5	N 33º 05' 376" / E 075º 19' 741"	Jammu & Kashmir: Patni Top	BD, HS, AT	NBRI (LWG)
Berberis lycium	250006	B6	N 33º 05' 349" / E 077º 19' 646"	Jammu & Kashmir: Patni top to Natha Top, Uttarakhand:	BD, HS, AT	NBRI (LWG)
Berberis macrosepala	262609	B190	NA	Sikkim: On left side of Chhangu Lake	TH	NBRI (LWG)
Berberis macrosepala	262612	B192	N 27°22.802' / E088°49.289'	Sikkim: On left side of Chhangu Lake	TH	NBRI (LWG)
Berberis macrosepala	262615	B194	N 27°22.572' / E088°49.370'	Sikkim: On left side of Chhangu Lake	TH	NBRI (LWG)
Berberis macrosepala	262616	B195	N 27°22.561' / E088°45.336'	Sikkim: On left side of Chhangu Lake	TH	NBRI (LWG)
Berberis pachyacantha	249804	B177	N 32º 15' 235" / E 077º 11' 014"	Himachal pradesh: Manali, Near Club House	TH, HS, AT	NBRI (LWG)
Berberis pachyacantha	249803	B179	N 32º 15' 328" / E 077º 10' 254"	Himachal pradesh: Manali, Behind Manu temlple	TH, HS, AT	NBRI (LWG)
Berberis pachyacantha	249801	B180	N 32º 13' 590" / E 077º 11' 328"	Himachal pradesh: Manali, Dhungri Van Vihar	TH, HS, AT	NBRI (LWG)
Berberis pachyacantha	249802	B181	N 32º 13' 608" / E 077º 11' 414"	Himachal pradesh: Manali, Near Hadimba Temple	TH, HS, AT	NBRI (LWG)
Berberis pachyacantha	249805	B184	N 32º 28' 768" / E 077º 07' 098"	Himachal pradesh: Manali, Palchan	TH, HS, AT	NBRI (LWG)
Berberis replicata	249301	B113	N 27º 27' 02" / E 92º 06' 65"	Arunachal Pradesh: W. Kameng Dist., Senge on Tawang Rd., Alt. 2731 m	BD, KNN, HS, AT	NBRI (LWG)
Berberis replicata	249302	B114	N 27º 26' 97" / E 92º 07' 07"	Arunachal Pradesh: W. Kameng Dist., Senge on Tawang Rd., Alt. 2864 m	BD, KNN, HS, AT	NBRI (LWG)

Berberis replicata	249303	B115	N 27º 26' 97" / E 92º 07' 07"	Arunachal Pradesh: W. Kameng Dist., Senge on Tawang Rd., Alt. 2864 m	BD, KNN, HS, AT	NBRI (LWG)
Berberis replicata	249304	B116	N 27º 26' 97" / E 92º 07' 07"	Arunachal Pradesh: W. Kameng Dist., Senge on Tawang Rd., Alt. 2864 m	BD, KNN, HS, AT	NBRI (LWG)
Berberis replicata	249305	B117	N 27º 26' 97" / E 92º 07' 07"	Arunachal Pradesh: W. Kameng Dist., near Senge on Tawang Rd., Alt. 2864 m	BD, KNN, HS, AT	NBRI (LWG)
Berberis replicata	249306	B118	N 27º 26' 97" / E 92º 07' 07"	Arunachal Pradesh: W. Kameng Dist., Senge on Tawang Rd., Alt. 2864 m	BD, KNN, HS, AT	NBRI (LWG)
Berberis replicata	249336	B132	N 27° 16' 39" / E 92° 25' 67"	Arunachal Pradesh: W.Kameng Dist, 1Km away from Bomdila on Tawang road Alt. 1875m	BD, KNN, HS, AT	NBRI (LWG)
Berberis replicata	248198	B147	N 27º 26' 90" / E 92º 06' 70"	Arunachal Pradesh: W. Kameng Dist., near Senge on Tawang Rd., Alt. 2594 m	BD, KNN, HS, AT	NBRI (LWG)
Berberis replicata	248200	B149	N 27º 27' 02" / E 92º 06' 65"	Arunachal Pradesh: W. Kameng Dist., Senge on Tawang Rd., Alt. 2731 m	BD, KNN, HS, AT	NBRI (LWG)
Berberis tinctoria	250055	B100	N 11º 24' 941" / E 076º43' 664"	Tamil Nadu: Ooty to Dodabetta	TH	NBRI (LWG)
Berberis tinctoria	250056	B101	N 11º 24' 888" / E 076º43' 414"	Tamil Nadu: Ooty to Dodabetta	TH	NBRI (LWG)
Berberis tinctoria	250057	B102	N 11º 24' 141" / E 076º44' 093"	Tamil Nadu: Ooty to Dodabetta	TH	NBRI (LWG)
Berberis tinctoria	250058	B103	N 11º 24' 946" / E 076º43' 760"	Tamil Nadu: Ooty to Dodabetta	TH	NBRI (LWG)
Berberis tinctoria	250059	B104	N 11º 24' 281" / E 076º44' 158"	Tamil Nadu: Dodabetta	TH	NBRI (LWG)
Berberis tinctoria	250040	B84	N 11º 22' 257" / E 076º45' 704"	Tamil Nadu: Ambadvarsha (On way from Conoor to Ooty)	TH	NBRI (LWG)
Berberis tinctoria	250041	B85	N 11º 22' 262" / E 076º45' 718"	Tamil Nadu: Aravakkadu (On way from Conoor to Ooty)	TH	NBRI (LWG)
Berberis tinctoria	250060	B86	N 11º 24' 941" / E 076º43'648"	Tamil Nadu: Dodabetta	TH	NBRI (LWG)
Berberis tinctoria	250042	B87	N 11º 23' 700" / E 076º43' 036"	Tamil Nadu: 5 km before Ooty	TH	NBRI (LWG)
Berberis tinctoria	250043	B88	N 11º 42' 551" / E 076º54' 328"	Tamil Nadu: 3 Km ahead of Conoor to Ooty	TH	NBRI (LWG)
Berberis tinctoria	250044	B89	N 11º 22' 679" / E 076º44' 024"	Tamil Nadu: 2 km before Ooty	TH	NBRI (LWG)
Berberis tinctoria	250045	B90	N 11º 22' 679" / E076º44' 024"	Tamil Nadu: Ooty	TH	NBRI (LWG)
Berberis tinctoria	250046	B91	N 11º 22' 283" / E 076º45' 819"	Tamil Nadu: 1Km ahead of Aravakkadu (On way from Conoor to Ooty)	TH	NBRI (LWG)
Berberis tinctoria	250047	B92	N 11º 22' 389" / E 076º45' 619"	Tamil Nadu: 2 Km ahead of Aravakkadu (On way from Conoor to Ooty)	TH	NBRI (LWG)
Berberis tinctoria	250051	B96	N 11º 00' 551" / E 076º56' 337"	Tamil Nadu: 1 Km ahead of Conoor to Ooty	TH	NBRI (LWG)
Berberis tinctoria	250052	B97	N 11º 23' 565" / E 076º43' 328"	Tamil Nadu: 8 Km ahead of Conoor to Ooty	TH	NBRI (LWG)
Berberis tinctoria	250053	B98	N 11º 22' 254" / E 076º45' 609"	Tamil Nadu: 4 Km ahead of Conoor to Ooty	TH	NBRI (LWG)
Berberis tinctoria	250054	B99	N 11º 24' 636" / E 076º42' 542"	Tamil Nadu: Ooty	TH	NBRI (LWG)
Berberis umbellata	249362	B158	N 30º 58' 20" / E 78º 26' 11"	Uttarakhand: Jankichatti, Himri forest across Kharsali Village Alt. 2534 m	BD, HS, AT	NBRI (LWG)
Berberis umbellata	249363	B159	N 30º 58' 37" / E 78º 26' 11"	Uttarakhand: Jankichatti, Himri forest across Kharsali Village Alt. 2544 m	BD, HS, AT	NBRI (LWG)
Berberis umbellata	249364	B160	N 30º 58' 20" / E 78º 26' 29"	Uttarakhand: Jankichatti, Himri forest across Kharsali Village Alt. 2542 m	BD, HS, AT	NBRI (LWG)
Berberis umbellata	249365	B161	N 30º 58' 24" / E 78º 26' 17"	Uttarakhand: Jankichatti, Himri forest across Kharsali Village Alt. 2534 m	BD, HS, AT	NBRI (LWG)
Berberis umbellata	249367	B163	N 30º 58' 37" / E 78º 30' 28"	Uttarakhand: Jankichatti, Himri forest across Kharsali Village Alt. 2535 m	BD, HS, AT	NBRI (LWG)
Berberis umbellata	249370	B166	N 30º 58' 48" / E 78º 26' 34"	Uttarakhand: Jankichatti, on way to Kharsali Village Alt. 2540 m	BD, HS, AT	NBRI (LWG)
Berberis umbellata	249373	B167	N 30º 58' 41" / E 78º 26' 27"	Uttarakhand: Jankichatti, Himri forest across Kharsali Village Alt. 2536 m	BD, HS, AT	NBRI (LWG)
Berberis wightiana	250048	B93	N 11º 36' 386" / E 076º85' 689"	Tamil Nadu: On way from Wellington to Ooty	TH	NBRI (LWG)
Berberis wightiana	250049	B94	N 11º 40' 262" / E 076º 65' 819"	Tamil Nadu: On way from Wellington to Ooty	TH	NBRI (LWG)
Berberis wightiana	250050	B95	N 11º 24' 683" / E 076º42' 538"	Tamil Nadu: Ooty	TH	NBRI (LWG)

Ficus

Species	Collection No.	DNA No.	Latitide / Longitude	Region	collectors name	Herbarium
Ficus benghalensis	249697	122	NA	Uttar Pradesh: Lucknow	LBC, UMS	NBRI (LWG)
Ficus benghalensis	249079	28	NA	Uttar Pradesh: Lucknow	LBC, UMS	NBRI (LWG)
Ficus benghalensis	249704	129	NA	Uttar Pradesh: Lucknow	LBC, UMS	NBRI (LWG)
Ficus benghalensis	249718	143	NA	Uttar Pradesh: Lucknow	LBC, UMS	NBRI (LWG)
Ficus benghalensis	249724	149	NA	Uttar Pradesh: Lucknow	LBC, UMS	NBRI (LWG)
Ficus benghalensis	250111	236	NA	Uttar Pradesh: Lucknow	LBC, UMS	NBRI (LWG)
Ficus benjamina	249069	18	NA	Uttar Pradesh: Lucknow	LBC, UMS	NBRI (LWG)
Ficus benjamina	249631	56	NA	Uttar Pradesh: Lucknow	LBC, UMS	NBRI (LWG)
Ficus carica	249800	225	NA	Uttar Pradesh: Lucknow	LBC, UMS	NBRI (LWG)
Ficus carica	249809	303	NA	Uttar Pradesh: Lucknow	LBC, UMS	NBRI (LWG)
Ficus carica	250252	377	NA	Uttar Pradesh: Lucknow	LBC, UMS	NBRI (LWG)
Ficus elastica	250246	371	NA	Uttar Pradesh: Lucknow	LBC, UMS	NBRI (LWG)
Ficus elastica	250281	406	NA	Uttar Pradesh: Lucknow	LBC, UMS	NBRI (LWG)
Ficus glomerata	249709	134	NA	Uttar Pradesh: Lucknow	LBC, UMS	NBRI (LWG)
Ficus glomerata	250181	306	NA	Uttar Pradesh: Lucknow	LBC, UMS	NBRI (LWG)
Ficus glomerata	250239	364	NA	Uttar Pradesh: Maharajganj	LBC, UMS	NBRI (LWG)
Ficus gomerata	250211	336	NA	Uttar Pradesh: Lucknow	LBC, UMS	NBRI (LWG)
Ficus hispida	250172	297	NA	Uttar Pradesh: Lucknow	LBC, UMS	NBRI (LWG)
Ficus hispida	250188	313	NA	Uttar Pradesh: Sitapur	LBC, UMS	NBRI (LWG)
Ficus hispida	250251	376	NA	Uttar Pradesh: Lucknow	LBC, UMS	NBRI (LWG)
Ficus religiosa	249705	130	NA	Uttar Pradesh: Lucknow	LBC, UMS	NBRI (LWG)
Ficus religiosa	249712	137	NA	Uttar Pradesh: Lucknow	LBC, UMS	NBRI (LWG)
Ficus religiosa	249716	141	NA	Uttar Pradesh: Lucknow	LBC, UMS	NBRI (LWG)
Ficus religiosa	249723	148	NA	Uttar Pradesh: Lucknow	LBC, UMS	NBRI (LWG)
Ficus religiosa	249725	150	NA	Uttar Pradesh: Lucknow	LBC, UMS	NBRI (LWG)
Ficus religiosa	250166	291	NA	Uttar Pradesh: Lucknow	LBC, UMS	NBRI (LWG)
Ficus religiosa	250194	319	NA	Uttar Pradesh: Lucknow	LBC, UMS	NBRI (LWG)
Ficus retusa	249701	126	NA	Uttar Pradesh: Lucknow	LBC, UMS	NBRI (LWG)
Ficus retusa	250203	328	NA	Uttar Pradesh: Lucknow	LBC, UMS	NBRI (LWG)
Ficus rumphi	249637	62	NA	Uttar Pradesh: Lucknow	LBC, UMS	NBRI (LWG)
Ficus rumphi	249732	157	NA	Uttar Pradesh: Lucknow	LBC, UMS	NBRI (LWG)
Ficus trigona	249700	125	NA	Uttar Pradesh: Lucknow	LBC, UMS	NBRI (LWG)
Ficus trigona	249733	158	NA	Uttar Pradesh: Lucknow	LBC, UMS	NBRI (LWG)
Ficus virens	249713	138	NA	Uttar Pradesh: Lucknow	LBC, UMS	NBRI (LWG)
Ficus virens	250277	402	NA	Uttar Pradesh: Lucknow	LBC, UMS	NBRI (LWG)

Gossypium

Species	Collection No.	DNA No.	Latitide / Longitude	Region	collectors name	Herbarium
Gossypium arboreum	250326	551	NA	Karnataka: Dharwad	LBC, JKA	NBRI (LWG)
Gossypium arboreum	250323	574	NA	Karnataka: Dharwad	LBC, JKA	NBRI (LWG)
Gossypium arboreum	250319	575	NA	Karnataka: Dharwad	LBC, JKA	NBRI (LWG)
Gossypium arboreum	250327	576	NA	Karnataka: Dharwad	LBC, JKA	NBRI (LWG)
Gossypium arboreum	250320	577	NA	Karnataka: Dharwad	LBC, JKA	NBRI (LWG)
Gossypium arboreum	250324	579	NA	Karnataka: Dharwad	LBC, JKA	NBRI (LWG)
Gossypium arboreum	221566	221566	NA	Gujarat	LBC, JKA	NBRI (LWG)
Gossypium arboreum	221567	221567	NA	Gujarat	LBC, JKA	NBRI (LWG)
Gossypium arboreum	221568	221568	NA	Gujarat	LBC, JKA	NBRI (LWG)
Gossypium arboreum	250308	AK235	NA	Karnataka: Dharwad	LBC, JKA	NBRI (LWG)
Gossypium arboreum	250301	DLSA17	NA	Karnataka: Dharwad	LBC, JKA	NBRI (LWG)
Gossypium barbadense	249001	249001	NA	Assam: Guwahati	LBC, JKA	NBRI (LWG)
Gossypium barbadense	249002	249002	NA	Assam: Bhagdabari	LBC, JKA	NBRI (LWG)
Gossypium barbadense	249003	249003	NA	Assam: Dakuapara	LBC, JKA	NBRI (LWG)
Gossypium barbadense	249004	249004	NA	Assam: Dakuapara	LBC, JKA	NBRI (LWG)
Gossypium barbadense	249005	249005	NA	Assam: Kamrup	LBC, JKA	NBRI (LWG)
Gossypium barbadense	249006	249006	NA	Assam: Andherijuli	LBC, JKA	NBRI (LWG)
Gossypium barbadense	249007	249007	NA	Assam:Andherijuli	LBC, JKA	NBRI (LWG)
Gossypium barbadense	249008	249008	NA	Assam: Pakharapara	LBC, JKA	NBRI (LWG)
Gossypium barbadense	249009	249009	NA	Assam: Lankeswar	LBC, JKA	NBRI (LWG)
Gossypium barbadense	249010	249010	NA	Assam: Lankeswar	LBC, JKA	NBRI (LWG)
Gossypium barbadense	249011	249011	NA	Assam: Sonaighuli	LBC, JKA	NBRI (LWG)
Gossypium barbadense	249012	249012	NA	Assam: Lankeswar	LBC, JKA	NBRI (LWG)
Gossypium barbadense	250345	SUVIN	NA	Tamil Nadu: TAU	LBC, JKA	NBRI (LWG)
Gossypium herbaceum	221547	221547	NA	Karnataka: Dharwad	LBC, JKA	NBRI (LWG)
Gossypium herbaceum	221557	221557	NA	Gujarat	LBC, JKA	NBRI (LWG)
Gossypium herbaceum	221573	221573	NA	Karnataka: Dharwad	LBC, JKA	NBRI (LWG)
Gossypium herbaceum	250307	AH41	NA	Karnataka: Dharwad	LBC, JKA	NBRI (LWG)
Gossypium herbaceum	259329	AH7GP	NA	Karnataka: Dharwad	LBC, JKA	NBRI (LWG)
Gossypium herbaceum	250318	DB312	NA	Karnataka: Dharwad	LBC, JKA	NBRI (LWG)
Gossypium herbaceum	250314	GC21	NA	Karnataka: Dharwad	LBC, JKA	NBRI (LWG)
Gossypium herbaceum	250322	GH-18-2LC	NA	Karnataka: Dharwad	LBC, JKA	NBRI (LWG)
Gossypium herbaceum	250331	H17	NA	Karnataka: Dharwad	LBC, JKA	NBRI (LWG)
Gossypium herbaceum	250325	JAYHELLAR	NA	Karnataka: Dharwad	LBC, JKA	NBRI (LWG)
Gossypium herbaceum	250330	RAHS127	NA	Karnataka: Dharwad	LBC, JKA	NBRI (LWG)
Gossypium herbaceum	250312	RAHS132	NA	Karnataka: Dharwad	LBC, JKA	NBRI (LWG)
Gossypium herbaceum	250332	RAHS14	NA	Karnataka: Dharwad	LBC, JKA	NBRI (LWG)
Gossypium herbaceum	250313	RAHSIPS187	NA	Karnataka: Dharwad	LBC, JKA	NBRI (LWG)
Gossypium herbaceum	250311	VAGAD	NA	Karnataka: Dharwad	LBC, JKA	NBRI (LWG)
Gossypium hirsutum	250347	AS-3	NA	Karnataka: Dharwad	LBC, JKA	NBRI (LWG)
Gossypium hirsutum	250304	JKC703	NA	Andhra Pradesh	LBC, JKA	NBRI (LWG)

Gossypium hirsutum	250305	JK-725	NA	Andhra Pradesh	LBC, JKA	NBRI (LWG)
Gossypium hirsutum	250342	JKC737	NA	Andhra Pradesh	LBC, JKA	NBRI (LWG)
Gossypium hirsutum	250315	JKC752	NA	Andhra Pradesh	LBC, JKA	NBRI (LWG)
Gossypium hirsutum	250334	JKC770	NA	Andhra Pradesh	LBC, JKA	NBRI (LWG)
Gossypium hirsutum	250346	JKC-771	NA	Andhra Pradesh	LBC, JKA	NBRI (LWG)
Gossypium hirsutum	250335	JKC777	NA	Andhra Pradesh	LBC, JKA	NBRI (LWG)
Gossypium hirsutum	250321	JK-783	NA	Andhra Pradesh	LBC, JKA	NBRI (LWG)
Gossypium hirsutum	250343	KC2	NA	Karnataka: Dharwad	LBC, JKA	NBRI (LWG)
Gossypium hirsutum	250341	LRA5166	NA	Andhra Pradesh	LBC, JKA	NBRI (LWG)
Gossypium hirsutum	250336	MCU-5	NA	Karnataka: Dharwad	LBC, JKA	NBRI (LWG)

Abbreviations for collectors names	
BD	Bhaskar Datt
TH	Tariq Hussain
KNN	K. N. Nair
HS	Harsh Singh
AT	Antariksh Tyagi
VS	Virendra shukla
LBC	Lal B. Chaudhary
UMS	Uma M Singh
JKA	JK Agri Genetics Limited
