# Supplementary material for: Universal Plant DNA Barcode Loci May Not Work in Complex Groups: A Case Study with Indian Berberis Species
Source: PLoS One. 2010 Oct 27;5(10):e13674. doi: 10.1371/journal.pone.0013674 (PMC2965122; doi:10.1371/journal.pone.0013674)
Supplement: Table S11 — Primer sequences used in this study (listed 5′- to 3′). (0.01 MB PDF) [file pone.0013674.s017.pdf]

Table S11

Locus	Direction	Sequence	Reference
ITS5a	Forward	CCTTATCATTTAGAGGAAGGA	(14)
ITS4	Reverse	TCCTCCGCTTATTGATATGC	(14)
matK 2.1a	Forward	ATCCATCTGGAAATCTTAGTTC	http://www.kew.org/barcoding/protocols.html
matK-NBRI	Forward	TCCCCATCCATCTGGAA	In this study
matK3.2r	Reverse	ATCTATCGATAATATCAGAAT	http://www.kew.org/barcoding/protocols.html
*rbcL*1F	Forward	ATGTCACCACAAACAGAAAC	(14)
*rbcL*724r	Reverse	TCGCATGTACCTGCAGTAGC	(14)
trnH-psbA	*trnH*	CGCGCATGGTGGATTCACAATCC	(14)
	psbA	GTTATGCATGAACGTAATGCT	(14)
